# 3,3′-Bipyridine and *N*-Alkyl Glycinates as Building Blocks for Cu-Based Bio-Inspired MOFs

**DOI:** 10.3390/polym18212698

**Published:** 2026-10-31

**Authors:** Darko Vušak, Mia Jurković, Katarina Ležaić, David Kučera-Čavara, Dijana Žilić, Željka Soldin, Dominique Agustin, Josipa Sarjanović, Jana Pisk, Biserka Prugovečki

**Affiliations:** 1 University of Zagreb Faculty of Science, Department of Chemistry, Horvatovac 102a, 10000 Zagreb, Croatia; darko.vusak@chem.pmf.unizg.hr (D.V.); m.jurkovic@utwente.nl (M.J.); k.lezaic@uu.nl (K.L.); zeljka.soldin@chem.pmf.unizg.hr (Ž.S.); josipa.sarjanovic@chem.pmf.unizg.hr (J.S.); jana.pisk@chem.pmf.unizg.hr (J.P.); 2 Department of Membrane Science and Technology, MESA+ Institute of Nanotechnology, Faculty of Science and Technology, University of Twente, P.O. Box 217, 7500 AE Enschede, The Netherlands; 3 Institute for Sustainable and Circular Chemistry, Faculty of Science, Utrecht University, Universiteitsweg 99, 3584CG Utrecht, The Netherlands; 4 Laboratory for Magnetic Resonances, Division of Physical Chemistry, Ruđer Bošković Institute, Bijeničkacesta 54, 10000 Zagreb, Croatia; dakuceracavara@irb.hr (D.K.-Č.); dzilic@irb.hr (D.Ž.); 5 LCC-CNRS ( Laboratoire de Chimie de Coordination), 205 Route de Narbonne, BP44099, CEDEX 4, 31077 Toulouse, France; dominique.agustin@iut-tlse3.fr; 6 Department of Chemistry, IUT Toulouse, University of Toulouse, 5 Allée du Martinet, 81100 Castres, France

**Keywords:** 3,3′-bipyridine, catalytic studies, bio-inspired metal–organic frameworks, *N*-alkylated glycine, X-ray structural analysis

## Abstract

Bio-inspired metal–organic frameworks have attracted considerable attention due to their structural versatility and potential applications in catalysis, sensing, and drug delivery. We report the synthesis, structural elucidation, thermal behaviour, and catalytic properties of the first copper(II)-based coordination polymers constructed from 3,3′-bipyridine and *N*-alkylated glycine derivatives. SCXRD showed that all compounds {[Cu(μ-MeGly)(NO_3_)(μ-3,3′-bpy)]·DMF·0.5H_2_O}*_n_* (**1a**), {[Cu(μ-MeGly)(NO_3_)(μ-3,3′-bpy)]·solvate}*_n_* (**1b**), {[Cu(μ-EtGly)(NO_3_)(μ-3,3′-bpy)]·DMF·0.5H_2_O}*_n_* (**2a**), and {[Cu(μ-EtGly)(NO_3_)(μ-3,3′-bpy)]·solvate}*_n_* (**2b**), (3,3′-bpy = 3,3′-bipyridine; MeGly = *N*-methylglycine; EtGly = *N*-ethylglycine) form three-dimensional coordination networks in which Cu(II) centres are interconnected through donor *N*-alkylated glycinate and 3,3′-bipyridine linkers. Bulk-phase characterisation confirmed the reproducible formation of **1a** and **2b**, while instability and solvent loss limited the isolation and characterisation of **2a** and **1b**. The bulk materials of **1a** and **2b** were further characterised by PXRD and **1a** by FTIR, ESR, SEM/EDS, TGA, simultaneous TGA-FTIR and CHN analysis. Catalytic studies of compound **1a** demonstrated moderate-to-high activity in the oxidation of cyclooctene, linalool, limonene, and cyclohexane. The catalytic results suggest that **1a** acts as a precatalyst, with the catalytically active species potentially forming under the reaction conditions; thus, the observed activity cannot necessarily be attributed to the intact MOF structure. These findings establish *N*-alkylated glycine derivatives as promising bio-inspired building blocks for the design of Cu-based MOFs and provide valuable insight into structure–property relationships and the potential role in catalysis.

## 1. Introduction

Metal–organic frameworks (MOFs) [1] are typically highly porous and crystalline hybrid organic–inorganic materials. Over the past three decades, this class of materials has been the subject of intense research and has profoundly influenced materials science. Owing to their structural versatility and rich chemical functionality, MOFs are inherently multifunctional, enabling applications in catalysis, gas storage, gas- and liquid-phase separations, air purification, water harvesting, electrochemical sensing, biofuel desulphurisation, the development of optical and luminescent materials, targeted drug delivery, enzyme-mimicking systems, etc. [2,3,4,5,6,7,8,9,10,11,12].

Conventional MOFs often have several drawbacks that limit their suitability for biomedical or environmentally oriented applications. Many lack sufficient biocompatibility, raise environmental safety concerns, or require costly, resource-intensive synthesis.

Recent research has increasingly focused on incorporating biological molecules—such as amino acids, peptides, sugars, and nucleic bases—into MOF architectures, yielding bio-inspired metal–organic frameworks [10,13]. Together with metal ions such as zinc, iron, copper, and zirconium, these biological molecules provide a good combination of biocompatibility and structural stability within the framework. Bio-inspired MOFs constructed from chiral biomolecules are particularly promising for enantioselective catalysis and chiral separations [14,15,16,17,18] and for controlled drug delivery [19].

Amino acids are attractive ligands because they are readily available, inexpensive, and well suited to producing larger quantities of MOFs for targeted applications. Their flexible conformations, multiple metal-binding modes, and capacity for diverse non-covalent interactions enable the design of frameworks with precisely tuned structures, pore sizes, and physicochemical properties. Amino acids can bind metals through both their carboxylate and amino groups, most often forming five-membered *O,N*-chelate rings, but they can also create extended structures through bi- or tridentate bridging [20,21]. Depending on the coordination mode, they form 1D chains or, less commonly, 2D and 3D metal–organic frameworks. Several metals, including Ni^2+^, Mn^2+^, Co^2+^, and Cu^2+^, form such extended networks with amino acids such as aspartic acid, glycine, phenylalanine, and glutamine [22,23,24,25]. 3D frameworks comprising metals and amino acids are rare but can arise when amino acids with additional side-chain functional groups bind metal ions, forming extended structures [26].

Dimensionality can also be enhanced by combining amino acids with additional organic ligands, such as heterocyclic bases like 4,4′-bipyridine and various dipyridine derivatives. By varying the heterocyclic bases (their molecular size and geometry), it becomes possible to systematically modulate pore dimensions, network topology, and the functional properties of the resulting materials. This class of metal–organic frameworks remains underexplored, with most published studies focusing on their synthesis and structural characterisation [27,28,29,30,31,32,33], while investigations of their properties remain limited. Only a few such MOFs have been studied for their magnetic properties [34,35,36,37], catalytic activity [15], chiral alcohol adsorption [17], luminescence [38,39], dielectric properties [40], and CO_2_ adsorption [41].

In particular, the catalytic application of metal–organic frameworks (MOFs) in selective oxidation reactions has attracted considerable attention in recent years, owing to their tunable structures, high surface areas, and accessible metal active sites. Recent advances in pristine MOFs with coordinatively unsaturated metal centres, MOF-supported materials, and MOF-derived catalysts for the thermocatalytic selective oxidation of organic compounds have been comprehensively reviewed and discussed [42]. Among copper-based MOFs, the selective oxidation of cyclooctene using tert-butyl hydroperoxide (TBHP) as the oxidant has been investigated in several Cu-containing frameworks. In particular, Cu_3_(BTC)_2_ (BTC = 1,3,5-benzenetricarboxylate) exhibited excellent catalytic performance, achieving complete conversion of cyclooctene after 120 h at 348 K in liquid toluene, with selectivity towards the cyclooctene epoxide gradually increasing to values exceeding 80% [43]. Copper MOFs have also shown remarkable activity in allylic oxidation reactions. The framework [Cu(bpy)(H_2_O)_2_(BF_4_)_2_(bpy)]*_n_* (bpy = 4,4′-bipyridine) exhibited promising catalytic performance in oxidising cyclohexene, using molecular oxygen as the sole oxidant. Under near-ambient, solvent-free conditions, the catalyst achieved hydroperoxide selectivities of up to 90%, highlighting its potential as an environmentally benign oxidation catalyst [44]. Similarly, the aerobic oxidation of cyclohexene under mild, solvent-free conditions has been investigated using the copper-based framework {[Cu_2_(OH)(BTC)(H_2_O)]·2H_2_O]}*_n_*, and the cobalt- and nickel-containing MOFs {[M_2_(DOBDC)(H_2_O)_2_]·8H_2_O}*_n_* (M = Co, Ni; DOBDC = 2,5-dihydroxyterephthalic acid), demonstrating the versatility of transition-metal MOFs as oxidation catalysts [45].

Metal–organic frameworks containing *N*-alkylated amino acids have not yet been structurally characterised in the literature, nor have copper(II) MOFs incorporating 3,3′-bipyridine together with an additional aminoacidato ligand [46]. 3,3′-bipyridine is poorly characterised structurally, especially in the area of MOFs, where only 11 crystal structures of 3D MOFs containing 3,3′-bipyridine are published in the CSD, and only in 2 crystal structures was an additional organic ligand used for coordination. In this context, the aim of the present work was to prepare copper(II) metal–organic frameworks incorporating *N*-alkylated glycinates (*N*-methyl- or *N*-ethylglycinate) and the heterocyclic base 3,3′-bipyridine. We examined the influence of synthetic methods, reactants, and solvents on the resulting products and their stability. X-ray structural analysis was conducted to elucidate how the alkyl chain length of the *N*-alkylglycine, the crystallisation solvent, and other experimental parameters affect the crystal packing of the resulting frameworks. Given the absence of structurally characterised MOFs of this type, this study is expected to make a significant contribution to the crystal engineering of bio-inspired metal–organic frameworks. Furthermore, given the growing interest in copper-based MOFs as oxidation (pre)catalysts, we investigated the catalytic properties of **1a**. This investigation is expected to broaden current understanding of structure–property relationships in amino acid-functionalised MOFs and further enrich the field of MOF-based catalysis.

## 2. Materials and Methods

### 2.1. General Information

The chemicals used in this study included copper(II) nitrate trihydrate, Cu(NO_3_)_2_·3H_2_O (Thermo Fisher Scientific, Waltham, MA, USA), copper(II) hydroxide, Cu(OH)_2_ (Alfa Aesar, Karlsruhe, Germany), *N*-methylglycine, HMeGly (Alfa Aesar, Karlsruhe, Germany), *N*-ethylglycine, HEtGly (Alfa Aesar, Karlsruhe, Germany), 3,3′-bipyridine, 3,3′-bpy (Tokyo Chemical Industry, Zwijndrecht, Belgium), methanol, CH_3_OH (CARLO ERBA Reagents, Milano, Italy), propan-1-ol, 1-PrOH (Thermo Fisher Scientific, Waltham, MA, USA), *N,N*-dimethylformamide, DMF (CARLO ERBA Reagents, Milano, Italy), ethylene glycol, EG (CARLO ERBA Reagents, Milano, Italy), sodium hydroxide, NaOH (Thermo Fisher Scientific, Waltham, MA, USA), potassium nitrate, KNO_3_ (Laphoma, Skopje, North Macedonia), potassium chloride, KCl (Zorka, Šabac, Serbia), ammonium chloride, NH_4_Cl (Kemika, Zagreb, Croatia), sodium chloride, NaCl (Alkaloid, Skopje, North Macedonia), cobalt(II) chloride hexahydrate, CoCl_2_·6H_2_O (Kemika, Zagreb, Croatia), magnesium nitrate hexahydrate, Mg(NO_3_)_2_·6H_2_O (Merck, Darmstadt, Germany), potassium carbonate, K_2_CO_3_ (Fisher Chemical, Waltham, MA, USA), magnesium chloride hexahydrate, MgCl_2_·6H_2_O (Kemika, Zagreb, Croatia), potassium acetate, CH_3_COOK (Kemika, Zagreb, Croatia), phosphorus(V) oxide, P_4_O_10_ (Acros Organics, Geel, Belgium), linalool, C_10_H_18_O (Tokyo Chemical Industry, Zwijndrecht, Belgium), limonene, C_10_H_16_ (Tokyo Chemical Industry, Zwijndrecht, Belgium), cyclooctene, C_8_H_14_ (Tokyo Chemical Industry, Zwijndrecht, Belgium), cyclohexane, C_6_H_12_ (GramMol, Zagreb, Croatia), acetophenone, C_8_H_8_O (Merck, Darmstadt, Germany), biphenyl, C_12_H_10_ (Sigma-Aldrich, Darmstadt, Germany), naphthalene, C_10_H_8_ (Sigma-Aldrich, Darmstadt, Germany), acetonitrile, C_2_H_3_N (T.T.T.), tert-butyl hydroperoxide, C_4_H_10_O_2_ (Sigma-Aldrich, Darmstadt, Germany), diethyl ether, C_4_H_10_O (T.T.T., Sveta Nedjelja, Croatia), and ethylbenzene, C_8_H_10_ (Sigma-Aldrich, Darmstadt, Germany).

Mechanochemical syntheses were carried out in a Retsch MM200 ball mill (Retsch GmbH, Haan, Germany) using Teflon jars (*V* = 14 mL) with a single stainless-steel ball (diameter 7 mm) at a vibration frequency of 25 Hz.

Single-crystal X-ray diffraction (SCXRD) data for compounds **1a**, **1b**, **2a** and **2b** were collected at 170 K on a Rigaku XtaLAB Synergy-S diffractometer (Rigaku Oxford Diffraction, Tokyo, Japan) equipped with a HyPix 6000HE detector using Cu*K*_α_ radiation (*λ* = 1.54056 Å). All datasets were processed using the CrysAlisPro software package (version 1.171.44.150a) [47].

Structure solution was performed using SHELXT (version 2018/2) [48], and refinements were carried out with SHELXL (version 2018/3) [49], both within the WinGX suite (version 2026.1) [50]. Structural visualisations were generated using Mercury (version 2026.1.1) [51]. Calculations of a volume fraction occupied by solvent molecules were performed using Solvate analyser incorporated into Mercury, with a probe radius of 1.00 Å and grid spacing of 0.30 Å. Geometrical analyses were performed using PLATON (version v1.17) [52].

All non-hydrogen atoms were refined anisotropically, except for the water-molecule oxygen atom in **1a** and **2a** and the atoms of the DMF molecule in **2a**. Most hydrogen atoms were placed at calculated positions based on the idealised geometries of their parent groups. Hydrogen atoms of water molecules were located in the difference Fourier map and refined with DFIX restraints on O–H distances (0.85(1) Å). In cases of disorder or insufficient data quality, selected water-hydrogen positions were fixed. The nitrate ion in **1a** was refined as a two-component disorder, with the nitrogen atom and one oxygen atom common to both disordered parts, and the other two oxygen atoms modelled over two positions. Occupancies of the disordered oxygen atoms were constrained to a total of 1 and refined to 0.56065 and 0.43935. Residual electron density in compounds **1b** and **2b**, corresponding to highly disordered crystallisation solvent molecules, was subtracted using the SQUEEZE procedure in the PLATON program suite [53]. One void in the unit cell was found for both **1b** and **2b**, with volumes of 50,039 Å^3^ and 49,843 Å^3^, respectively, and 7421 and 18,759 electrons were removed by the SQUEEZE procedure, respectively. The large difference in the number of omitted electrons is caused by the weaker diffraction and lower-quality data for **1b**.

Room-temperature powder X-ray diffraction (PXRD) measurements for **1a**, **2b**, and variable relative humidity experiments for **1a** (see Section 2.3) were performed on a Malvern Panalytical Aeris diffractometer (Malvern Panalytical B.V., Almelo, The Netherlands) in reflection geometry using Cu*K*_α_ radiation (*λ* = 1.54056 Å) and equipped with a PixCel1D detector. Data were collected over the 2*θ* range of 5–30°, with a step size of 0.022° and a counting time of 15.0 s per step. The diffraction pattern of **2b** was collected with and without Kapton foil. Variable-temperature PXRD experiments for **1a** were conducted on a Malvern PANalytical Empyrean diffractometer (Malvern Panalytical B.V., Almelo, The Netherlands) equipped with a PIXcel3D Medipix3 detector and Cu*K*_α_ radiation. Diffraction data were collected in reflection mode over a 2*θ* range of 3–30° with a step size of 0.0066° and a counting time of 9.5 s per step. The sample was measured in an Anton Paar TTK 600 chamber (Anton Paar GmbH, Graz, Austria) under an air atmosphere. The temperature varied from 30 to 190 °C, with diffraction patterns recorded at 10 °C intervals. Prior to each measurement, the selected temperature was stabilized for 10 min. After completion of the heating cycle and data collection at 190 °C, the sample was cooled to 30 °C.

The PXRD patterns were processed and evaluated using the HighscorePlus software package (version 5.2) [54] and DataViewer (version 1.9a) [55].

The FTIR spectrum of compound **1a** was recorded over the range 4000–400 cm^−1^ at a resolution of 4 cm^−1^ using a Thermo Scientific Nicolet iS50 FTIR spectrometer (Thermo Fisher Scientific, Waltham, MA, USA) with a diamond ATR accessory.

Thermogravimetric analysis (TGA/DSC) of compound **1a** was conducted using a simultaneous TGA/DSC analyzer, Mettler-Toledo TGA/DSC3+ (Mettler-Toledo, Columbus, OH, USA). The sample was placed in alumina pans (70 μL) and heated in the nitrogen (50 mL min^−1^) or oxygen flow (100 mL min^−1^) from room temperature to 600 °C at 10 °C min^−1^.

The TGA-FTIR analysis of compound **1a** was performed using a simultaneous TGA/DSC Mettler-Toledo TGA/DSC3+ coupled with a Thermo Scientific Nicolet iS50 FTIR spectrometer equipped with a 10 cm pathlength nickel-plated aluminum gas cell. The gas cell and the glass-lined stainless-steel transfer line were maintained at 250 °C. Approximately 20 mg of the sample was placed in alumina pans (70 μL) and heated in flowing nitrogen (50 mL min^−1^) from room temperature to 400 °C at 10 °C min^−1^. FTIR spectra were obtained over the range 4000–400 cm^−1^ with a resolution of 8 cm^−1^.

Continuous-wave (CW) electron spin resonance (ESR) X-band measurements of compound **1a** were performed on a Bruker Magnettech ESR5000 spectrometer (Bruker, Billerica, MA, USA) at room temperature (298 K) and under a cooled nitrogen stream (100 K). All measurements were conducted at a microwave frequency of 9.3–9.5 GHz, a magnetic-field modulation amplitude of 0.5 mT, and a modulation frequency of 100 kHz.

CHN analysis of **1a** was performed on Perkin Elmer 2400 Series II CHNSO Analyzer (PerkinElmer, Waltham, MA, USA).

The microstructural characteristics of sample **1a** were analysed using a JEOL JSM-7000F field-emission scanning electron microscope (JEOL Ltd., Tokyo, Japan). The samples were mounted on appropriate sample holders using conductive carbon tape and analysed as a relatively uniform nanoparticle structure in the micrometre range without further preparation. The imaging was performed using a secondary electron detector (SEI) under the following conditions: accelerating voltage 15 kV at the working distance of 10 mm. The micrographs were acquired at various magnifications and locations, with one image taken at 10,000× chosen as representative. Elemental composition of the sample was obtained using Oxford INCA Energy 350 EDS spectrometer (Oxford Instruments Ltd., High Wycombe, Buckinghamshire, UK) coupled with the SEM microscope. EDS spectra were collected from representative surface regions at 15 kV accelerating voltage and standard probe current settings. The spectra were taken at various locations of the sample in order to obtain representative elemental composition.

Catalytic activity of **1a** was investigated in the oxidation of cyclooctene, linalool, limonene and cyclohexane. Reactions were carried out for 5 h, with aliquots withdrawn at predetermined time intervals (0, 50, 90, 180 and 300), and each reaction was repeated 3 times to ensure reproducibility of the results. Prior to analysis, samples were diluted with diethyl ether. Oxidation products from cyclooctene, linalool and limonene were analysed using an Agilent 7820A GC equipped with a DB-Wax capillary column (IUT Paul Sabatier, Toulouse, France), whereas those from cyclohexane were analysed using an Agilent 8860 GC fitted with an HP-5 capillary column (Faculty of Science, Zagreb, Croatia). All reactions were performed with 0.1 mmol (46 mg) of (pre)catalyst **1a**, 20 mmol of substrate and 40 mmol of oxidant. Tert-butyl hydroperoxide in aqueous solution was used as the oxidant. Reactions were performed both in the absence and in the presence of acetonitrile (3 mL) as a cosolvent to evaluate its influence on catalytic performance. Quantification was achieved using internal standards: acetophenone for cyclooctene and cyclohexane, naphthalene for limonene and ethylbenzene for linalool. Calibration curves were done for each substrate and desired product. Chromatograms showing the retention times of each substrate and product are presented in Figures S1–S3.

Control experiments with individual components and their mixture: Additional control experiments for the oxidation of cyclohexane were performed using Cu(NO_3_)_2_, methyl glycinate (MeGly), 3,3′-bipyridine (3,3′-bpy), and a mixture of these components in the corresponding Cu:MeGly molar ratio. In each experiment, 0.1 mmol of the individual component or component mixture was used with 20 mmol of cyclohexane and 40 mmol of oxidant.

### 2.2. Synthetic Procedures

Four new copper(II) compounds containing an *N*-alkylated glycinate ligand and 3,3′-bipyridine were prepared: {[Cu(μ-MeGly)(NO_3_)(μ-3,3′-bpy)]·DMF·0.5H_2_O}*_n_* (**1a**), {[Cu(μ-MeGly)(NO_3_)(μ-3,3′-bpy)]·solvate}*_n_* (**1b**), {[Cu(μ-EtGly)(NO_3_)(μ-3,3′-bpy)]·DMF·0.5H_2_O}*_n_* (**2a**), and {[Cu(μ-EtGly)(NO_3_)(μ-3,3′-bpy)]·solvate}*_n_* (**2b**). All coordination polymers were obtained by solution-phase synthesis at 100 °C for 20 min. Copper(II) nitrate trihydrate, copper(II) hydroxide, the corresponding *N*-alkylated glycinate, and 3,3′-bipyridine were dissolved in mixtures of three solvents (H_2_O, CH_3_OH or 1-PrOH, and DMF or EG) in a molar ratio of 1:1:2:2. After heating, the reaction mixtures were left to stand at room temperature in air, whereupon the dark-blue solutions gradually yielded single crystals suitable for SCXRD analysis. Compounds **1a** and **2b** were also prepared via liquid-assisted grinding. Copper(II) nitrate trihydrate, copper(II) hydroxide, *N*-methylglycine, and 3,3′-bipyridine were placed in Teflon vessels and 39 μL of DMF was added. This volume of DMF corresponds to 0.5 mmol, matching the reaction stoichiometry for the preparation of **1a**.

Cu(NO_3_)_2_·3H_2_O(s) + Cu(OH)_2_(s) + 2 HMeGly(s) + 2 3,3′-bpy(l) + 2 DMF(l) → 2 {[Cu(μ-MeGly)(NO_3_)(μ-3,3′-bpy)]·DMF·0.5H_2_O}*_n_*(s) + 4 H_2_O(l)

A similar procedure was used for the mechanochemical synthesis with *N*-ethylglycine, yielding **2b**. Grinding was carried out for 15 min using a Retsch MM200 vibrational mill. A dark-blue powder was obtained in both synthetic procedures and was immediately analysed by powder X-ray diffraction.

Solution-phase synthesis of {[Cu(μ-MeGly)(NO_3_)(μ-3,3′-bpy)]·DMF·0.5H_2_O}*_n_* (**1a**) and {[Cu(μ-MeGly)(NO_3_)(μ-3,3′-bpy)]·solvate}*_n_* (**1b**). Copper(II) hydroxide (24.4 mg, 0.250 mmol), copper(II) nitrate trihydrate (60.8 mg, 0.252 mmol), *N*-methylglycine (46.5 mg, 0.521 mmol) and 3,3′-bipyridine (78.1 mg, 0.500 mmol) were placed in a beaker. 10 mL of water, 10 mL of DMF and 5 mL of methanol were added, and the reaction mixture was heated at 100 °C for 20 min. The reaction mixture was filtered, and the filtrate was placed in two crystallising dishes—one narrow-walled (approx. 2 cm in diameter) and another with a wide opening (approx. 5 cm in diameter). After several weeks, dark blue needles of **1a** formed in the narrow-walled crystallising dish. Crystals of **1a** are stable outside solution. Yield is 66.8% (151 mg). In the wider crystallising dish, after a few days of evaporation, blue crystals of **1b** formed on the rim of the solution. Crystals of **1b** decompose outside solution (and in the absence of solvent vapours). After several days or weeks, **1a** forms at the bottom of the solution. If a mixture of DMF and water (both 10 mL) was used as solvents with the same procedure, the product is less predictable in the wider crystallising dish, sometimes producing **1a** and on rare occasions **1b** as the first product, which later reconstructs into **1a**.

Mechanochemical synthesis of {[Cu(μ-MeGly)(NO_3_)(μ-3,3′-bpy)]·DMF·0.5H_2_O}*_n_* (**1a**). Copper(II) hydroxide (24.2 mg, 0.248 mmol), copper(II) nitrate trihydrate (60.4 mg, 0.250 mmol), *N*-methylglycine (45.3 mg, 0.508 mmol), 3,3′-bipyridine (78.9 mg, 0.505 mmol) and DMF (39 μL, 0.50 mmol) were placed in a Teflon milling jar (*V* = 14 mL) containing a single stainless-steel ball (diameter 7 mm). Milling was carried out for 15 min at a frequency of 25 Hz at room temperature. The yield after cleaning the jar is 85.4% (193 mg).

IR (ATR, cm^−1^): 3329 (w), 3252 (w), 3134 (w), 3072(w), 2939 (w), 1662 (m), 1604 (s), 1508 (w), 1471 (w), 1432 (m), 1333 (s), 1256 (m), 1229 (m), 1208 (m), 1158 (m), 1099 (m), 1055 (m), 1026 (m), 986 (m), 969 (m). 919 (w), 864 (w), 836 (m), 708 (m), 663 (m), 633 (m), 517 (m), 551 (s), 507 (m),458 (m), 417 (m).

Elemental analysis calculated for C_32_H_44_N_10_O_13_Cu_2_; *w*(C) = 42.52%, *w*(H) = 4.91%, *w*(N) = 15.50%. Found: *w*(C) = 42.40%, *w*(H) = 4.91%, *w*(N) = 15.21%.

Solution-phase synthesis of {[Cu(μ-EtGly)(NO_3_)(μ-3,3′-bpy)]·DMF·0.5H_2_O}*_n_* (**2a**) and {[Cu(μ-EtGly)(NO_3_)(μ-3,3′-bpy)]·solvate}*_n_* (**2b**). Copper(II) hydroxide (24.4 mg, 0.250 mmol), copper(II) nitrate trihydrate (60.3 mg, 0.250 mmol), *N*-ethylglycine (51.5 mg, 0.499 mmol) and 3,3′-bipyridine (78.1 mg, 0.500 mmol) were placed in a beaker. 10 mL of water, 10 mL of DMF and 5 mL of propan-1-ol were added, and the reaction mixture was heated at 100 °C for 20 min. The reaction mixture was filtered, and the filtrate was left to evaporate. After several days, a very small amount of **2a** formed in a mixture with **2b**. Crystals of **2a** are stable outside solution, whereas those of **2b** decompose. If a mixture of 10 mL of water, 5 mL of methanol and 5 mL of ethylene glycol, or a mixture of 10 mL of water, 5 mL of propan-1-ol and 5 mL of ethylene glycol, or a mixture of 10 mL of water, 10 mL of DMF and 5 mL of methanol was used as solvent with the same procedure, pure **2b** was obtained. Variations in the shape and size of the crystallising dish did not yield different results.

Mechanochemical synthesis of {[Cu(μ-EtGly)(NO_3_)(μ-3,3′-bpy)]·solvate}*_n_* (**2b**). Copper(II) hydroxide (24.4 mg, 0.250 mmol), copper(II) nitrate trihydrate (60.4 mg, 0.250 mmol), *N*-ethylglycine (51.5 mg, 0.499 mmol), 3,3′-bipyridine (78.9 mg, 0.505 mmol) and DMF (39 μL, 0.50 mmol) were placed in a Teflon milling jar (*V* = 14 mL) containing a single stainless-steel ball (diameter 7 mm). Milling was carried out for 15 min at a frequency of 25 Hz at room temperature.

Considering that only **1a** was produced in a pure form and is stable outside solution, most analyses were performed on this compound. All solution-based synthetic procedures yielded high-quality crystals, enabling us to collect high-quality X-ray single-crystal diffraction data for all obtained compounds. **2b** was also prepared with high reproducibility, but it loses solvent molecules immediately after drying.

### 2.3. Control of Relative Humidity–Stability Testing of Compound ***1a***

The stability of compound **1a** was examined by placing the samples in chambers with varying relative humidity. The samples were transferred into Eppendorf tubes, which were then placed in 20 mL glass vials. The vials contained saturated solutions of various salts (KNO_3_, KCl, NH_4_Cl, NaCl, CoCl_2_, Mg(NO_3_)_2_, K_2_CO_3_, MgCl_2_, CH_3_COOK, NaOH), water, or phosphorus(V) oxide, which established precisely defined humidity conditions at 20 °C, as presented in Table S1 [56]. After 14 days, the samples were analysed by powder X-ray diffraction.

## 3. Results

### 3.1. Synthesis and Crystallisation

**Figure 1:**
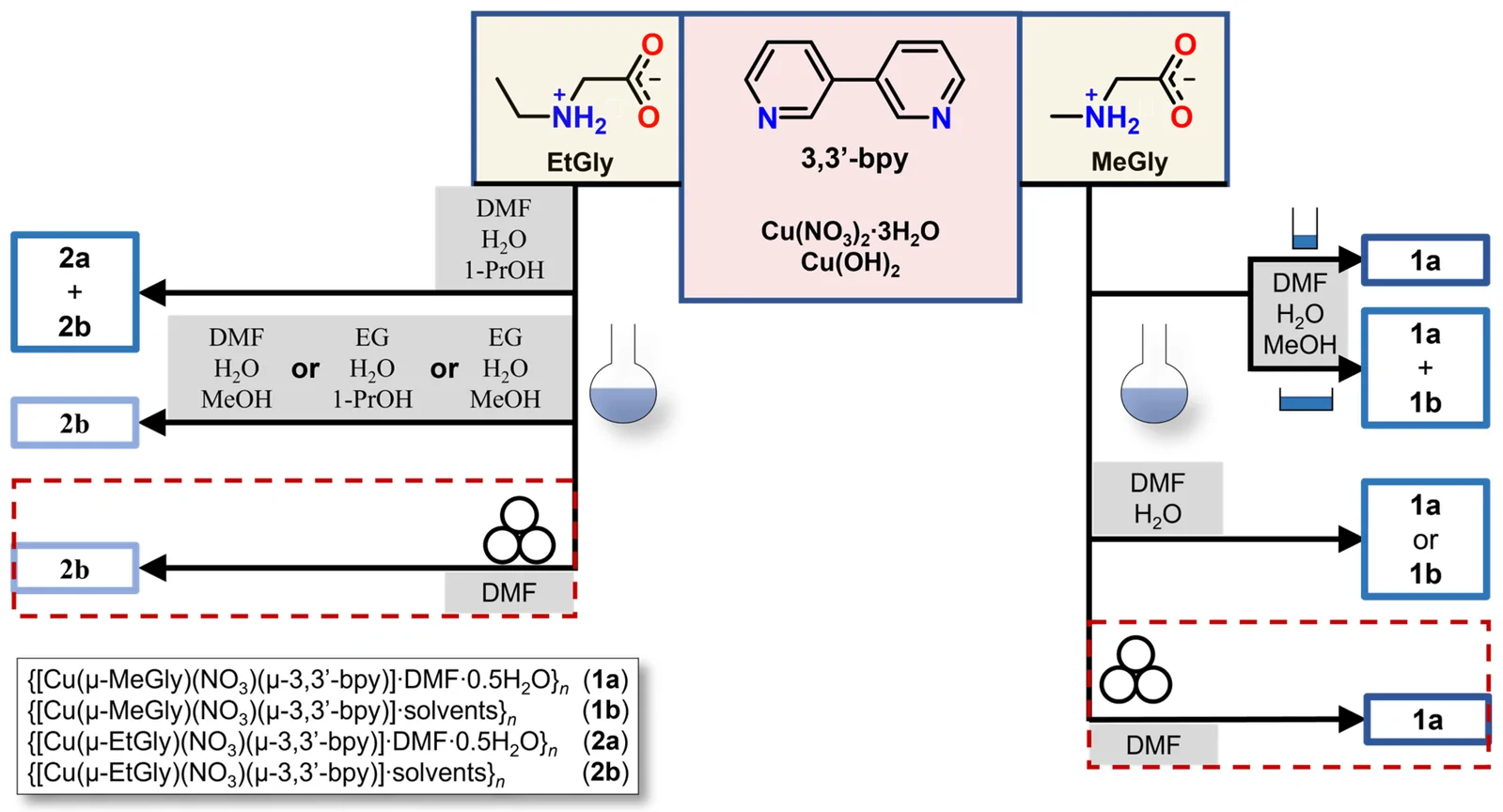
Scheme of synthetic procedures.

Figure 1 presents a schematic overview of the synthetic procedures. Two coordination compounds, **1a** and **1b**, were prepared by dissolving copper(II) nitrate trihydrate, copper(II) hydroxide, 3,3′-bipyridine, and *N*-methylglycine in a mixed solvent system consisting of water, methanol, and *N,N*-dimethylformamide (DMF). Compound **1b** crystallised quickly along the rim of the evaporating solution, whereas compound **1a** was obtained by slow crystallisation through gradual solvent evaporation at room temperature. After several repetitions of the synthesis, it was observed that **1b** forms more often on the rim of a wider crystallising dish, whereas in a crystallising dish with narrow walls it is possible to produce completely pure **1a**. Pure **1b** was obtained on rare occasions using a DMF/H_2_O solvent mixture in a beaker, but after a few days, **1a** started to form within the same solution. **1b** was extremely unstable upon isolation, undergoing rapid solvent loss. Compound **1a** was also synthesised mechanochemically (Figure S4). Five reactants were used in the mechanochemical procedure: three solids—copper(II) nitrate trihydrate, copper(II) hydroxide, and *N*-methylglycine—and two liquids, 3,3′-bipyridine and DMF. This approach yielded a fully pure product. The *η*-parameter, defined as the ratio of the volume of liquid to the total mass of solid reactants [57], was 0.82 μL mg^−1^. Since both liquid reactants are incorporated into the crystal structure of compound **1a**, the synthesis yielded a dry product.

The definition of the *η*-parameter in the presented synthesis does not accurately reflect the synthesis parameters. In porous materials, such as **1a**, a certain amount of liquid is adsorbed and becomes part of the solid (crystal), thereby continuously changing the reaction parameters, such as the vapour pressure and the wetness of the reaction mixture. With this in mind, we propose an additional metric as an estimated parameter—the volume of excess (unreacted) liquid divided by the mass of solids in the final product (*η*_ex_). In this way, we can calculate the initial ratio of liquids and solids (*η*_0_) and the final ratio (*η*_ex_), after the reaction and adsorption, which, in general, can be expressed as the volume of the liquid remaining in the reaction mixture divided by the mass of the solid products (Equation (1)). In the special case where part of the initial liquid remains unreacted, the remaining volume is calculated as the total added volume minus the volume of all liquids that reacted or were adsorbed by the solid (Equation (2)).

(1) $$\eta_{\mathrm{ex}}=\frac{V_{\mathrm{remaining liquid}}}{m_{\mathrm{final solid}}}$$

(2) $$\eta_{\mathrm{ex}}=\frac{V_{\mathrm{total}}-V_{\mathrm{reacted}}}{m_{\mathrm{final solid}}}$$

In the synthesis of **1a**, the initial liquids are DMF and 3,3′-bipyridine, and the solids are *N*-methylglycine, copper(II) hydroxide and copper(II) nitrate trihydrate with *η*_0_ = 0.822 μL mg^−1^. After the reaction, DMF and 3,3′-bipyridine are completely incorporated into the solid. Water is also a product of the reaction (partly absorbed in the final solid product), so we can calculate the excess water assuming 100% conversion (Table 1). The final product consists of **1a** and excess water, yielding a new value of an estimated parameter *η*_ex_ = 0.080 μL mg^−1^. We propose this additional, supplementary metric because there is a gap in describing reactions that involve adsorption of liquid reactants during the reaction. The proposed metrics may be useful when the amount of liquid changes considerably due to the reaction and when the composition of the final product is known.

In subsequent synthetic procedures, *N*-ethylglycine (HEtGly) was used alongside the previously employed reactants. Using the solvent mixture water–propan-1-ol–DMF, compound **2a** crystallised in a mixture with **2b**, whereas in mixtures water–methanol–DMF, water–methanol–EG, and water–propan-1-ol–EG resulted in the crystallisation of **2b**. Interestingly, HEtGly, which differs from HMeGly by a single CH_2_ group, prefers to form the cubic framework, **2b**, under various synthetic conditions, including in a narrow-walled crystallising dish. Additionally, **2b** was synthesised by liquid-assisted grinding, but it decomposes immediately upon removal from the milling jar, indicated by the additional diffraction maxima (Figure S5). After 2 months of aging in the air at room temperature, **2b** decomposes into a previously unobserved crystalline phases (Figure S6). It was established that compounds **1a** and **2a** are stable outside their mother liquor, whereas **1b** and **2b** proved unstable upon isolation and drying, losing solvent molecules. Overall, the synthesis results demonstrate that compounds **1a** and **2a** exhibit sufficient stability to be isolated in solid form, whereas their analogues **1b** and **2b** show pronounced sensitivity to solvent removal.

### 3.2. Crystal Structures and Topological Analysis

**Figure 2:**
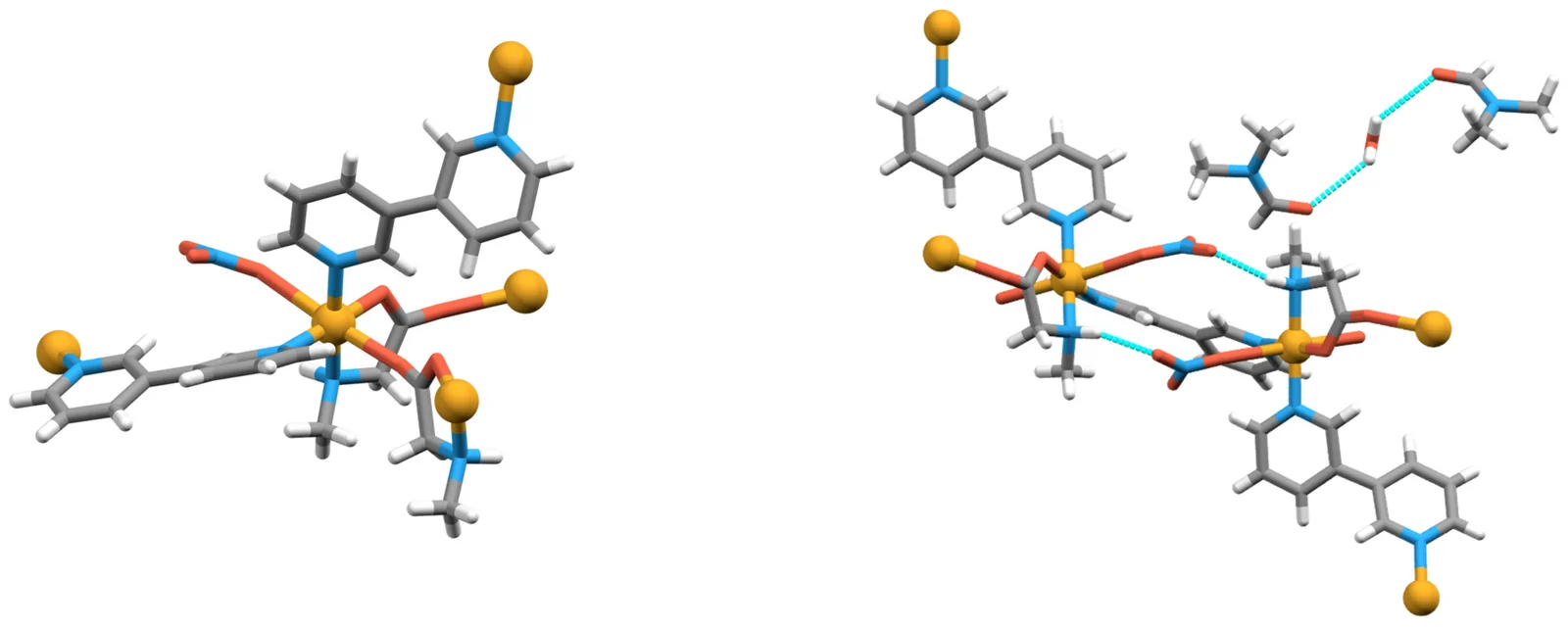
Secondary building unit in compound **1a** (**left**) and hydrogen bonds in **1a** (**right**).

The isostructural compounds **1a** and **2a** crystallize in the orthorhombic crystal system, adopting the non-centrosymmetric space group *Ccc*2. Further crystallographic details are provided in the Supplementary Information (Table S2). The three-dimensional neutral metal–organic framework is formed by polymerizing octahedral secondary building units comprising copper(II) ions, two bridging 3,3′-bipyridine ligands, two negatively charged *N*-alkylated glycinate ligands, and one coordinated nitrate anion. *N*-alkylated glycinate binds to copper(II) atoms via the amino group and an oxygen atom of the carboxylate group, forming a five-membered chelate ring. The carboxylate group acts as a bridging ligand, with a second oxygen atom binding to a neighboring copper(II) atom (Figure 2).

In compounds **1a** and **2a**, the equatorial Cu–O distances are 1.947(6) and 1.945(19) Å, while the Cu–O bond lengths in axial positions range from 2.403(4) to 2.752(14) Å, consistent with the Jahn–Teller effect. The equatorial Cu–N bond lengths span 2.003(6) to 2.022(4) Å. The 3,3′-bipyridine bridging ligands align above one another in a zig-zag pattern. Two symmetry-independent 3,3′-bipyridines bridge the copper(II) ions and adopt two conformations: synperiplanar (torsion angle C206–C205–C205′–C206′ = −23.1(12) and 24.5(6)°) and anticlinal (torsion angle C106–C105–C105′–C106′ = 140.0(6) and 140.2(15)°). Atom numbering schemes for **1a** and **2a** are provided in Figure S7 of the Supplementary Information. Due to methylation of nitrogen, only one N–H group is available as a hydrogen-bond donor. Two adjacent secondary building units are linked by two N–H∙∙∙O_nitrate_ intraframework hydrogen bonds in a ring motif *R*_2_^2^(12) (Figure 2 and Figure S8).

**Figure 3:**
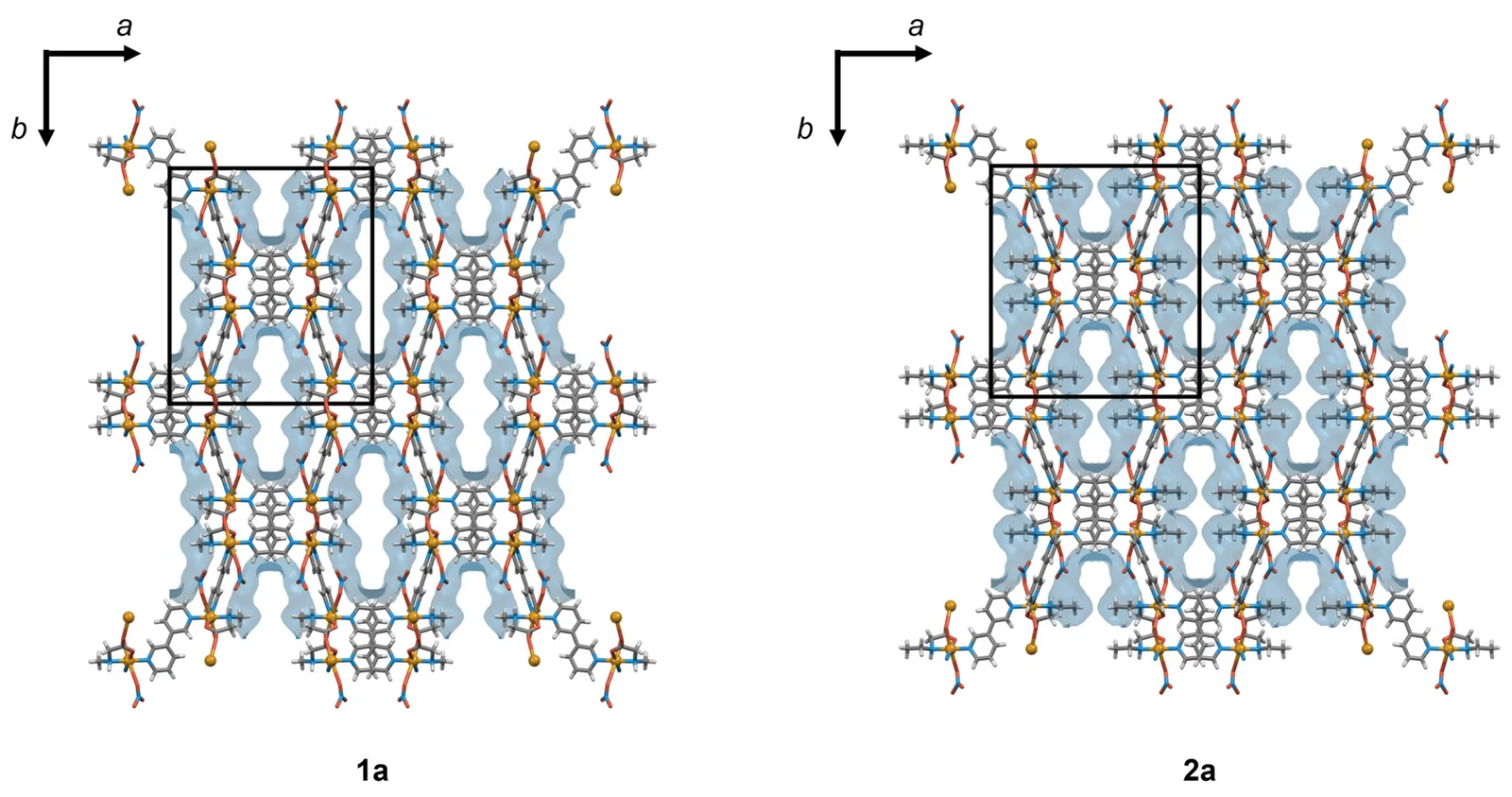
Crystal packing of compounds **1a** and **2a**. The one-dimensional infinite channels filled with solvent molecules are shown in blue.

Crystallisation solvent molecules are located within one-dimensional infinite channels in the crystal structures. Water and DMF molecules form O_water_–H∙∙∙O_DMF_ hydrogen bonds, but there are no strong intermolecular interactions with the coordination framework. In both **1a** and **2a**, the channels contain DMF and water molecules and occupy 32.5% and 31.4% of the unit cell volume, respectively (Figure 3). A topological analysis of the structures of **1a** and **2a**, based on a simplified underlying net constructed from the Cu(II) metal nodes, reveals the well-known *irl* topology.

**Figure 4:**
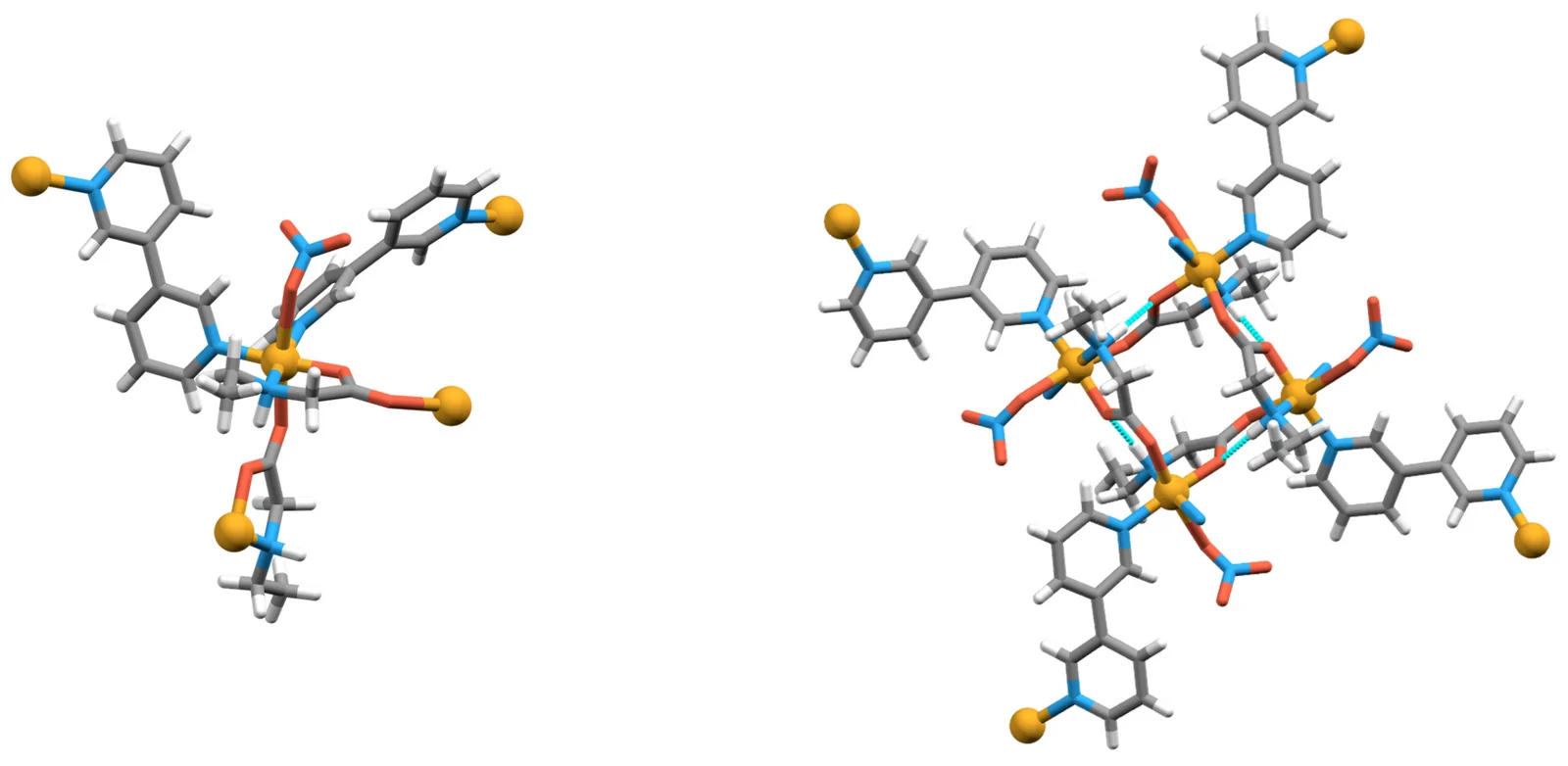
Secondary building unit in **2b** (**left**) and four-nuclear copper cluster in **2b** (**right**).

Compounds **1b** and **2b** are isostructural and crystallise in the cubic crystal system, within the relatively uncommon centrosymmetric space group *Ia*$\bar{3}$*d*. The Cambridge Structural Database (version Nov 2025 + 2 updates) contains 10,061 cubic structures, of which only 161 entries belong to this space group, highlighting the structural distinctiveness of these systems. In both **1b** and **2b**, the SBUs contain two bridging 3,3′-bipyridine molecules, two *N*-alkylated glycinates, and one nitrate ion (Figure 4).

The observed bond lengths—elongated axial Cu–O distances (2.279(2)–2.708(5) Å) and shorter equatorial Cu–O (1.931(3) and 1.950(2) Å) and Cu–N (2.000(4)–2.030(2) Å) bonds—are consistent with a tetragonally distorted octahedron, a hallmark of the Jahn–Teller effect in d^9^ Cu^2+^ systems. The 3,3′-bipyridine ligands serve as bridging linkers, here adopting only an anticlinal conformation (torsion angles C106–C105–C115–C116 = 139.8(4)°and −137.5(2)°), which plays an essential role in directing long-range connectivity and is the main difference in connectivity compared with orthorhombic **1a** and **2a**. Their spatial arrangement promotes the formation of a neutral, three-dimensional metal–organic framework, assembled from recurrent linkages of identical secondary building units. This network, which propagates through both *N*-alkylglycinate and 3,3′-bipyridine ligands, is characteristic of frameworks in which mixed donor sets (*N*,*O*) cooperate to stabilise extended architectures. Atom numbering schemes for **1b** and **2b** are provided in Figure S9 in the Supplementary Information. **1b** and **2b** form intraframework N–H∙∙∙O_carboxylate_ hydrogen bonds within four-nuclear copper atom clusters in a ring motif *R*_4_^4^(16) (Figure 4 and Figure S10).

**Figure 5:**
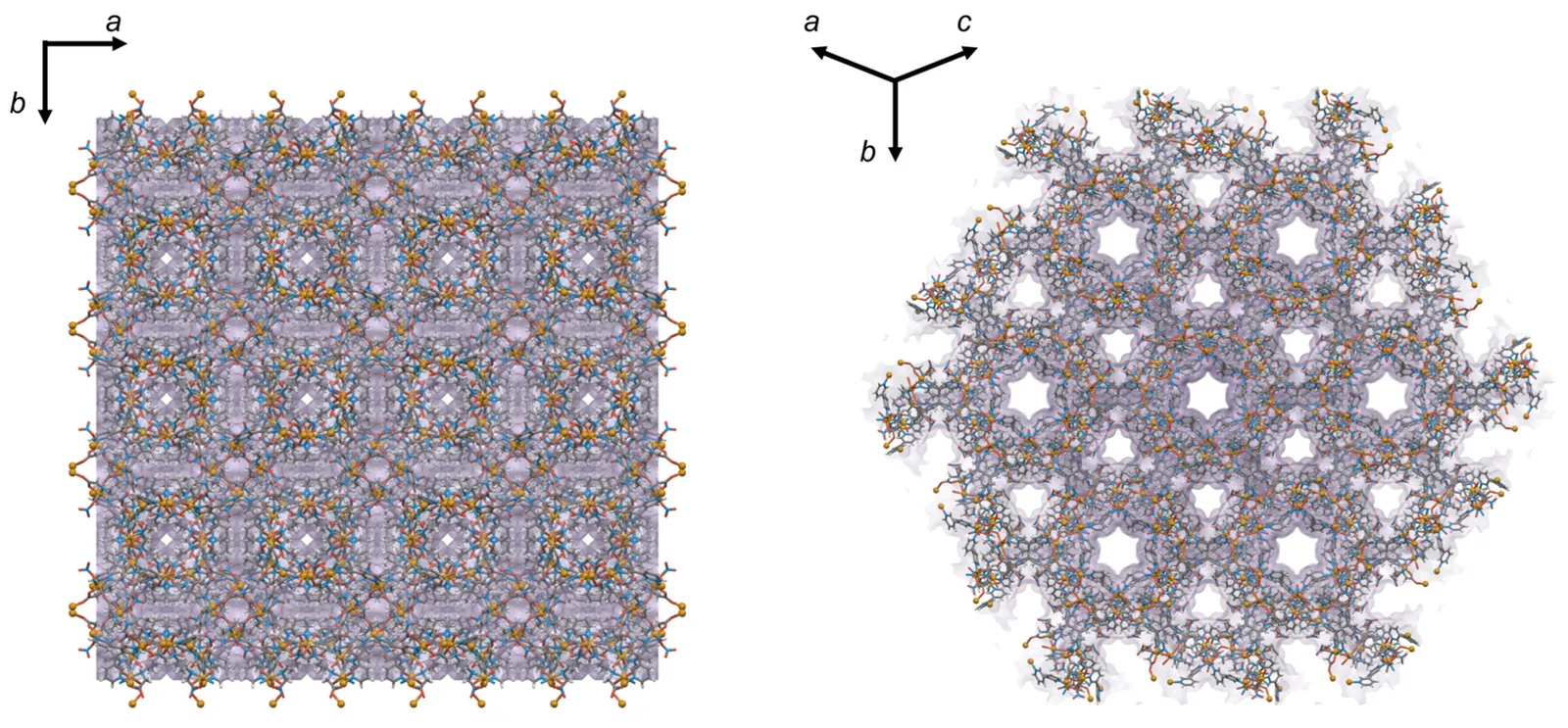
Crystal packing of compound **2b** viewed along the crystallographic *c*-axis (**left**) and along the [111] direction (**right**). Three-dimensional infinite channels filled with solvent molecules are shown in purple.

The coordination polymer contains three-dimensional, infinite channels that host highly disordered solvent molecules that cannot be modelled crystallographically due to dispersed electron density within the pores. The solvent-filled pore volume within the unit cell of compounds **1b** and **2b** amounts to 62.0% and 60.6% of the unit cell volume, respectively. The solvent molecules form 3D channels within the pores of the 3D metal–organic framework shown in Figure 5. A topological analysis of the structures of compounds **1b** and **2b**, based on a simplified underlying net constructed from the Cu(II) metal nodes, reveals the well-known *gie* topology.

### 3.3. Infrared Analysis of Compound ***1a***

The recorded IR spectrum (Figure S11) is consistent with the crystal structure of compound **1a**. The very broad absorption band in the spectral range 3329–3134 cm^−1^ corresponds to O–H stretching vibrations of lattice water, overlapping with the N–H stretching vibration of the coordinated *N*-methylglycinate ligand. Weak bands around 3000 cm^−1^ are attributed to aromatic C–H stretching vibrations of the 3,3′-bipyridine ligand. Weak absorption connected with the aliphatic C–H stretching vibrations of the methyl groups in the *N*-methylglycinate ligand and the DMF solvent molecule is observed at 2939 cm^−1^. The characteristic C=O stretching band of the DMF solvate molecule is observed at 1662 cm^−1^. Absorption bands corresponding to the antisymmetric *ν*_as_(COO^−^) and symmetric *ν*_s_(COO^−^) stretching vibrations of the *N*-methylglycinate ligand are observed at 1604 cm^−1^ and 1432 cm^−1^, respectively [58]. The latter overlaps with the broad, strong absorption band of the monodentate nitrate ligand centred at 1333 cm^−1^; therefore, a reliable interpretation of the Δ*ν*(COO^−^) value in terms of the coordination mode of the carboxylate group [59] is not possible. The absorption band at 1026 cm^−1^ and the aforementioned broad band at 1333 cm^−1^ are assigned to the coordinated nitrate ion [60].

The thermal stability of compound **1a** was studied under nitrogen by TGA/DSC (Figure S12). The compound is thermally stable up to 49 °C, and the corresponding TG curve reveals a five-step decomposition, with poorly resolved second, third, and fourth weight-loss steps. The first step, between 49 °C and 94 °C, is accompanied by a 2.17% weight loss, corresponding to the elimination of lattice water (calc. 1.99%). In the temperature range from 94 °C to 194 °C, 16.00% of the initial mass is lost, corresponding to the release of lattice DMF molecules (calc. 16.18%). Continuous thermal degradation of the compound **1a** occurs after DMF loss, and a total mass loss of 82.85% was observed at 600 °C. The DSC curve of the compound exhibits three broad signals: one endothermic peak at 186 °C and two exothermic peaks at 198 °C and 294 °C. The endothermic signal corresponds to the elimination of DMF molecules from the crystal structure. The coupled TGA-IR technique was used to monitor evolved gases in real time. The total infrared response is displayed by the Gram–Schmidt profile of compound **1a** (Figure S13a), while the TG–FTIR data are presented as a three-dimensional plot (Figure S13b). IR spectra recorded at selected temperatures were compared with reference gas-phase spectra to identify the evolved species. The spectra collected during the second mass-loss step (10–16 min) confirmed the elimination of lattice DMF. The evolved DMF was identified by comparison with the OMNIC gas-phase library spectra (OMNIC 9 software, Waltham, Massachusetts, USA), primarily based on the characteristic carbonyl stretching band at around 1700 cm^−1^. At higher temperatures (after 15.5 min), the characteristic absorption band of CO_2_ at approx. 2350 cm^−1^ is dominant in the IR spectra, consistent with the thermal decomposition of the *N*-methylglycinate ligands.

The thermogravimetric analysis of compound **1a** was also performed in an oxygen atmosphere for the mechanochemically prepared sample (Figure S14). We measured two samples: one as synthesised, directly from the milling jar (possibly moist), and the other after drying. The first, moist sample was measured without settling of the scale, so we could determine the amount of the residual moisture after milling. The sample loses water immediately (moisture and crystallisation water) from room temperature up to 150 °C, with a recorded loss of mass of 8.98% (calc. 9.23%). In the range 150–360 °C, there are several consecutive steps corresponding to the loss of DMF molecules and the decomposition of organic ligands and nitrate ions. The residual mass of CuO is 16.90% (theoretical 16.30%). The dried sample shows similar behaviour, with loss a of water of 1.89% (calc. 1.99%) in the temperature range 30–110 °C. The loss of DMF and decomposition occur in the temperature range of 140–360 °C. The residual mass of CuO is 17.79% (calc 17.61%).

### 3.4. Stability of Compound ***1a*** Under Different Temperature and Relative Humidity Conditions

**Figure 6:**
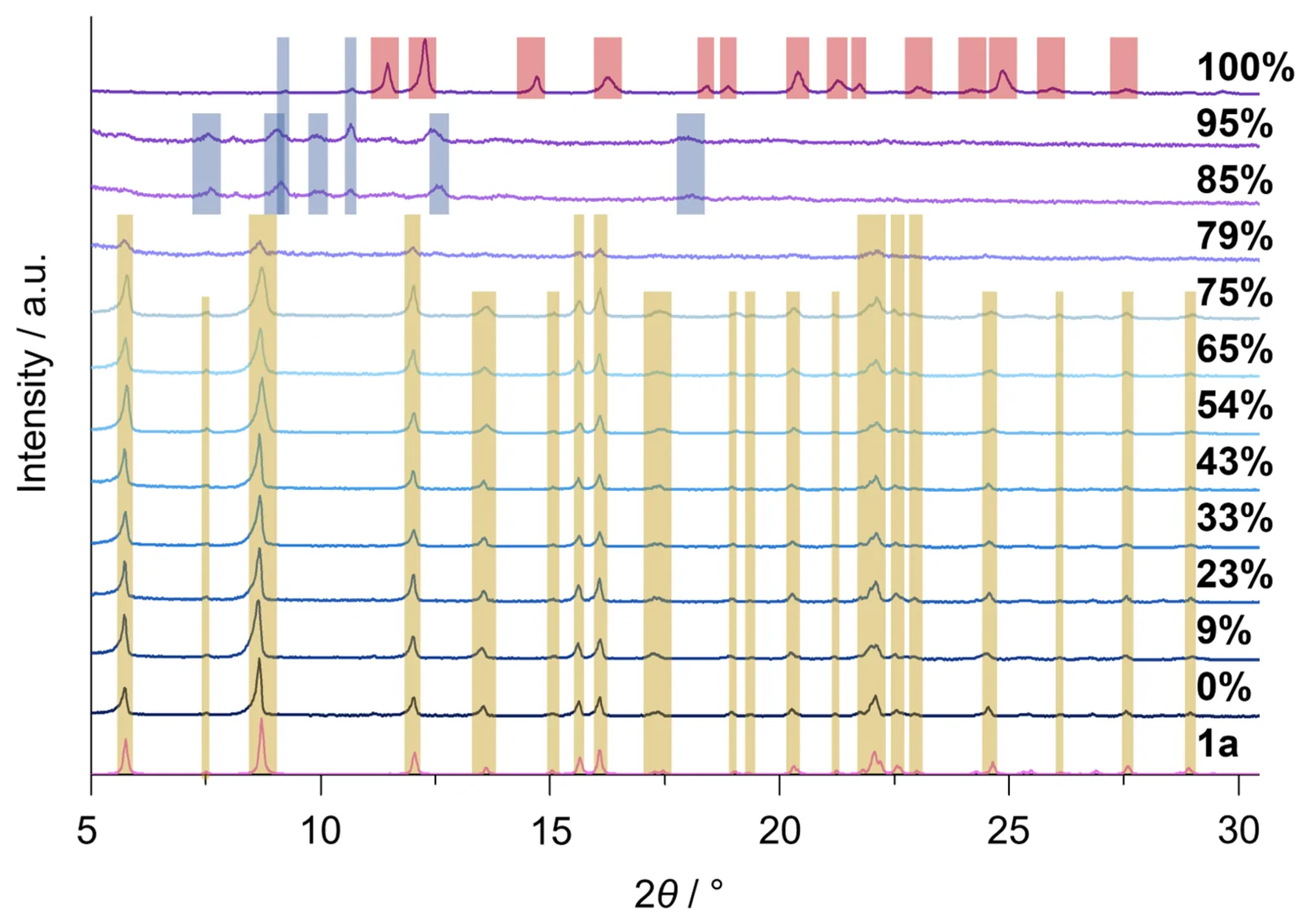
PXRD patterns of a sample of **1a** at different relative humidities. Yellow lines represent diffraction maxima of **1a**, while blue and red represent previously undescribed phases.

A comparison of the experimental PXRD patterns at different relative humidities with the calculated diffraction pattern of compound **1a** is shown in Figure 6. Exposure of compound **1a** to varying relative humidity at a constant temperature of 20 °C, followed by powder X-ray diffraction (PXRD) analysis, revealed that the compound remains structurally stable over the range of 0–75% relative humidity. At 79%, diffraction maxima associated with the structure of **1a** are still observable; however, their intensities are considerably reduced compared with those at lower humidity levels, indicating the onset of partial structural changes. At higher humidity levels, particularly between 85% and 95%, the diffraction pattern no longer shows features typical of compound **1a**. Instead, one or more previously unobserved crystalline phases appear, characterised by broader, less intense diffraction maxima that do not overlap with those of the original structure. Peaks shift to higher 2*θ* values in previously unobserved crystalline phases, indicating smaller unit cell parameters. Low intensities of diffraction maxima possibility indicate that at higher relative humidity **1a** partially dissolves in condensed water. At 100% relative humidity, a previously unobserved crystalline phase forms, displaying clear diffraction peaks, indicating a more significant structural change under saturated moisture conditions, probably due to dissolution and recrystallisation. The new phases do not correspond to any known phase (such as [Cu(Sar)_2_] coordination compounds published in CSD).

**Figure 7:**
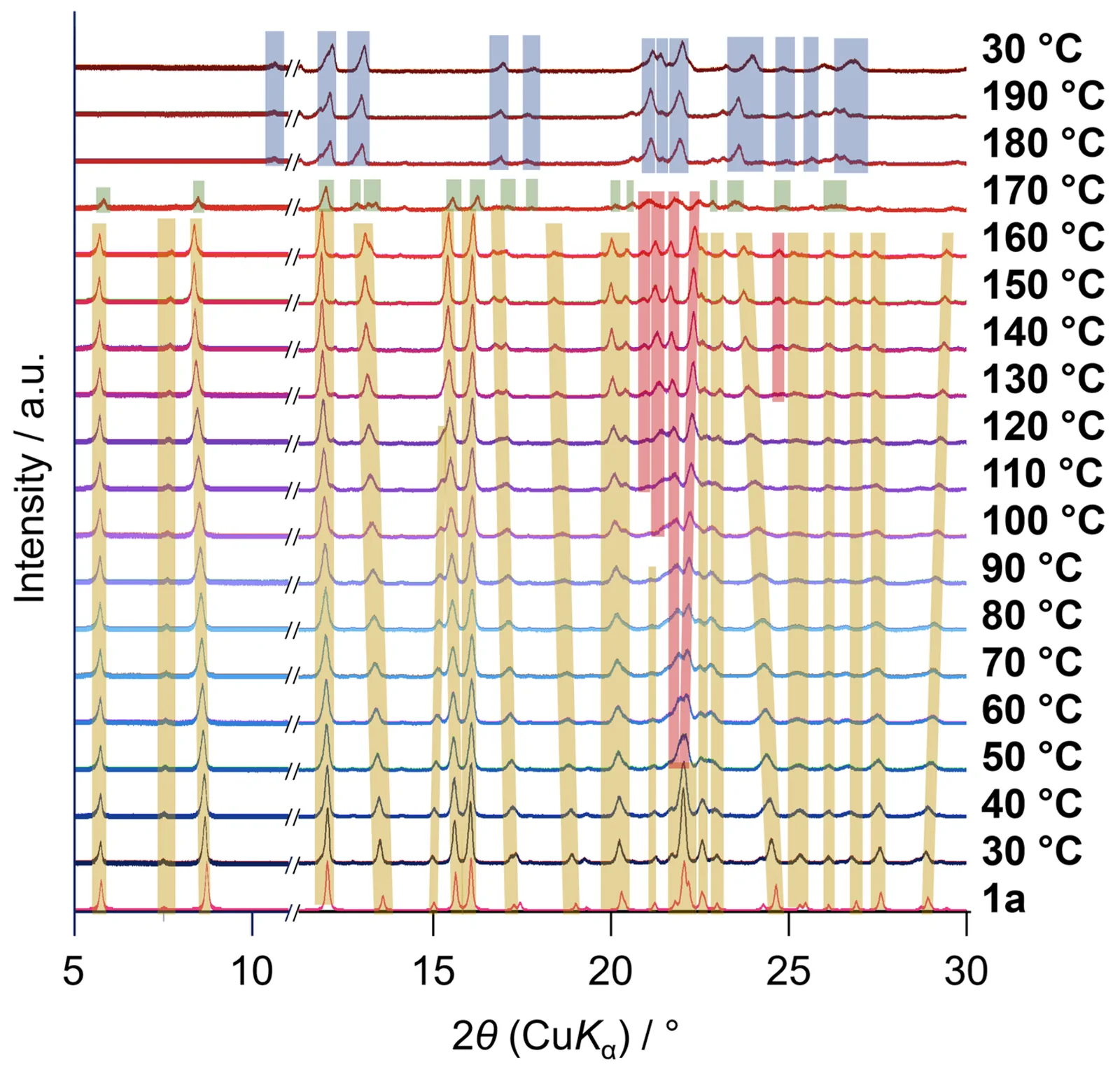
PXRD patterns of a sample of **1a** at different temperatures. The diffraction pattern on top, at 30 °C, is the pattern of the sample after the experiment and after cooling to 30 °C. Yellow lines represent diffraction maxima of **1a**, while blue, green and red represent previously undescribed phases. Parts of the PXRD pattern calculated from the crystal structure, divided by the broken lines, are not on the same intensity scale.

Temperature-dependent PXRD is presented in Figure 7 and can be correlated with thermogravimetric analysis. Small changes in the PXRD patterns begin at 50 °C and continue until 160 °C, corresponding to solvent desorption. Most peaks remain in similar positions, indicating that the framework is preserved within this temperature range. At 170 °C, there is a significant decrease in diffraction maxima and a shift in most peaks towards higher 2*θ* angles. Due to the loss of solvent molecules, it is possible a more compact structure emerges. At approximately the same temperature, TGA showed DMF desorption. At 180 °C and 190 °C, and after cooling back to 30 °C, there are no significant changes in the PXRD pattern.

### 3.5. ESR Experiments

**Figure 8:**
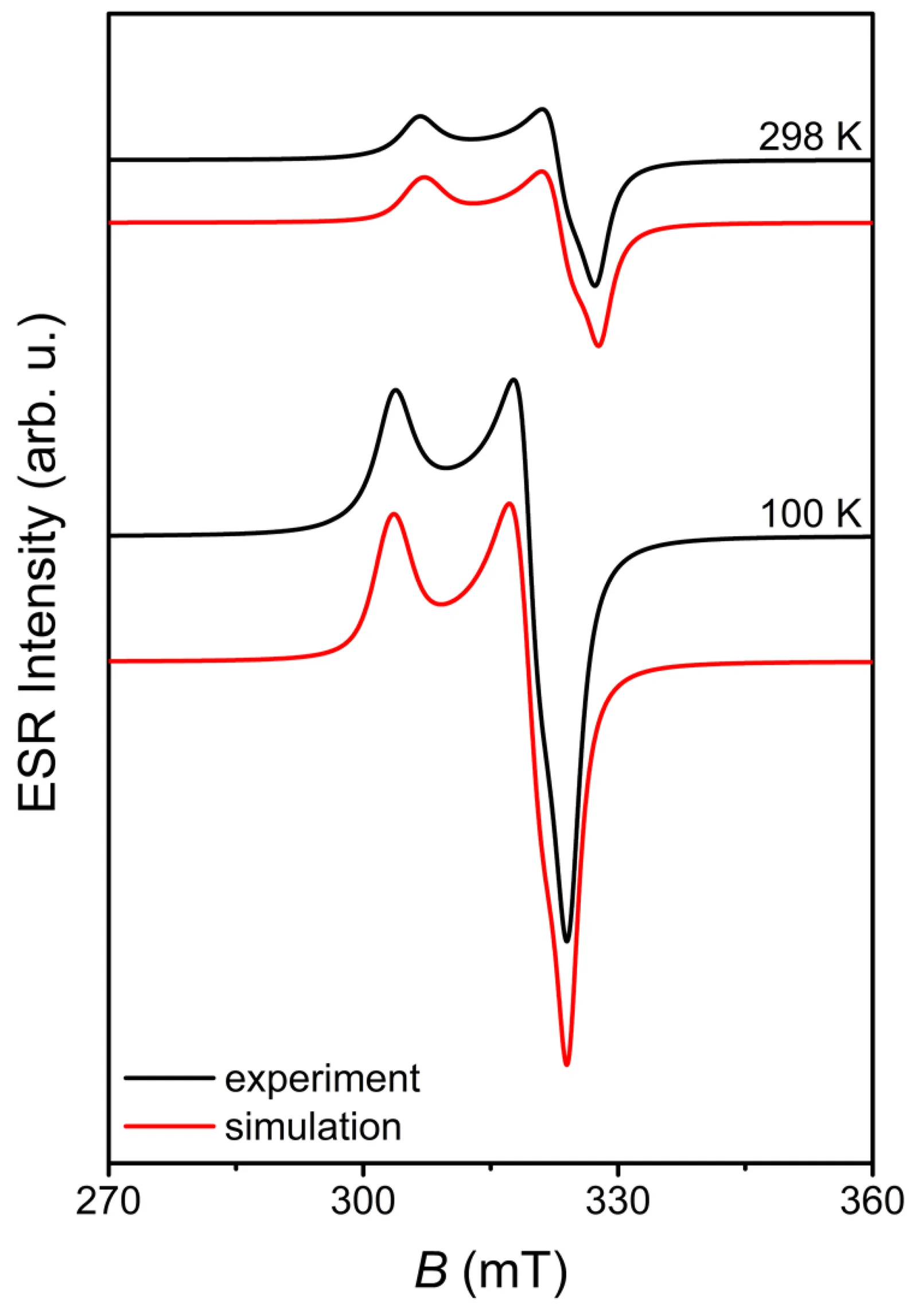
Experimental (black lines) and simulated (red lines) ESR spectra for compound **1a**.

ESR experimental spectra of the investigated sample of **1a** measured at room temperature (298 K) and at liquid nitrogen temperature (100 K) are shown in Figure 8. Corresponding simulations are also presented, carried out with EasySpin 6.0.12 software [61].

For the investigated compound **1a**, a spin state *S* = ½ was assigned to the Cu(II) ion [62]. The ESR spectra were simulated using only a Zeeman term in the spin-Hamiltonian:

***H*** = *μ*_B_ · ***B*** · ***g*** · ***S***, (3)

where *μ*_B_ is the Bohr magneton constant, ***B*** is the external magnetic field, ***g*** is the ***g***-tensor, and ***S*** is the electron spin operator for the unpaired copper electron [63]. For the investigated sample, the Lorentzian line with the peak-to-peak linewidth (*lw*_pp_) was used in the simulations to model isotropic broadening. Anisotropic broadening was modelled using ***g***-strain to account for distributions in the ***g***-tensor parameters, and *H*-strain to represent field-independent broadening contributions such as unresolved hyperfine splittings. Table 2 summarises the parameters obtained from these simulations.

The ESR spectra of the investigated compound **1a** exhibit a characteristic *g*-tensor anisotropy with the sequence *g*_z_ > *g*_y_ ≈ *g*_x_ > 2.0023, confirming a $d_{x^{2}-y^{2}}$ ground state. These results are consistent with a Jahn-Teller elongated octahedral coordination geometry around the copper(II) ions, in which the similar values of *g*_x_ and *g*_y_ approximately reflect distorted axial symmetry in the equatorial plane [64]. The ***g***-tensor for complex **1a** remained constant upon cooling from 298 K to 100 K, suggesting the temperature stability of the local Cu(II) environment. Although the molecular structure of the **1a** does not change significantly, decreasing temperature leads to a slight narrowing of the linewidths, reflecting reduced thermal motion, as typically observed for copper(II) complexes in frozen or solid states [65].

### 3.6. Catalytic Performance of ***1a***

**Figure 9:**
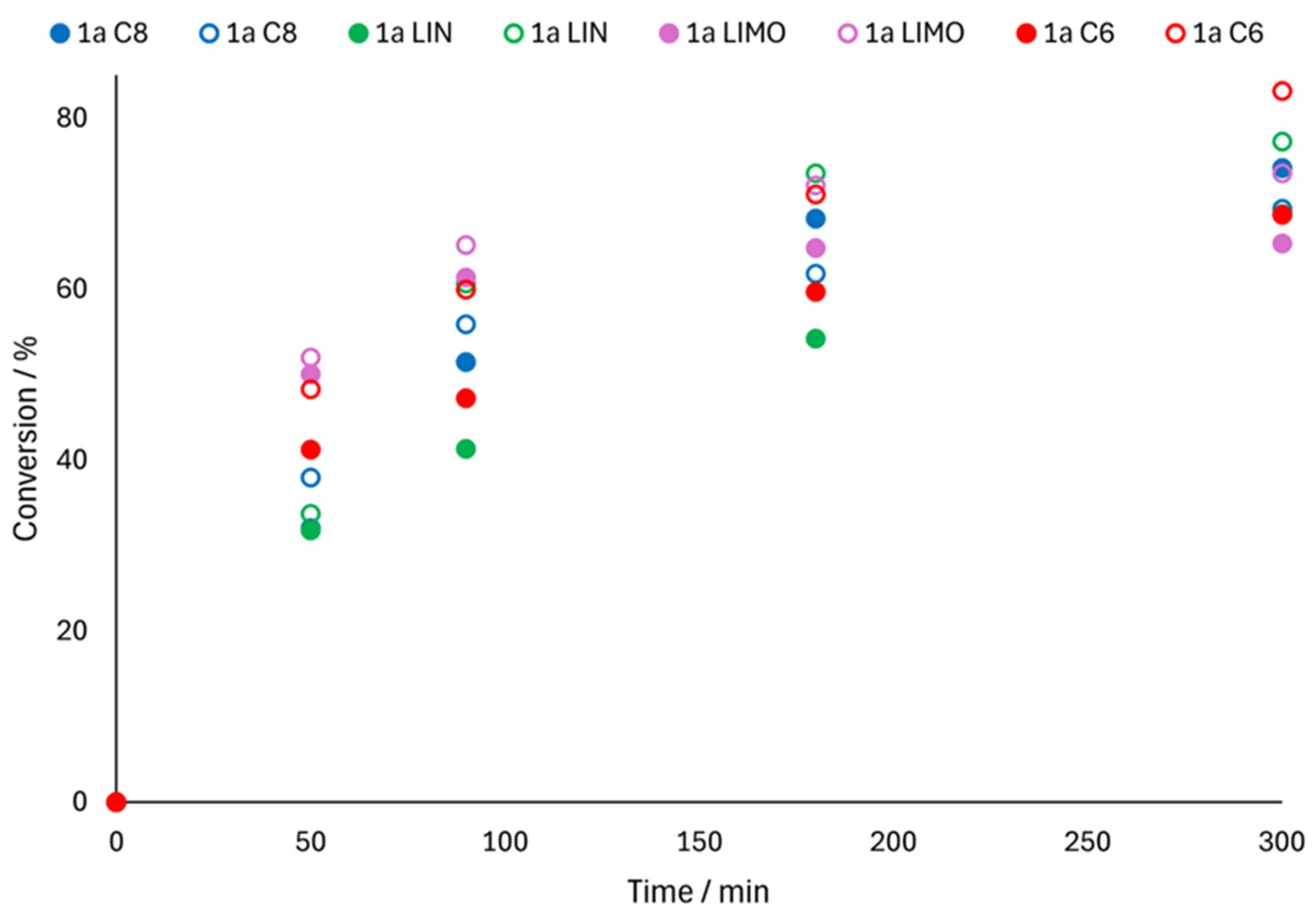
Kinetic profiles for cyclooctene (C8), linalool (LIN), limonene (LIMO) and cyclohexane (C6) conversion. A full dot represents the reaction without the addition of acetonitrile, while the empty dot represents the reaction with the addition of acetonitrile.

The catalytic performance of compound **1a** was evaluated across four representative substrates, namely cyclooctene, linalool, limonene, and cyclohexane, to probe its activity across different types of oxidation reactions (epoxidation, allylic oxidation, and C–H activation). All reactions were carried out over 5 h using TBHP as the oxidant, both in an aqueous medium and in the presence of acetonitrile as a co-solvent, allowing assessment of solvent effects on catalytic behaviour. As the reaction medium was biphasic, comprising distinct organic and aqueous phases, the organic phase was selectively analysed. Previous studies have demonstrated that the majority of the desired products are preferentially distributed in the organic phase. Accordingly, gas chromatography (GC) was employed to characterise the product distribution in the organic phase. The (pre)catalyst is completely soluble in the reaction media, and upon completion of the reaction it could not be filtered and further reused. Overall, the (pre)catalyst shows consistently moderate-to-high substrate conversions across all four substrates, typically in the range of 65–83% (Figure 9, Figures S15 and S16, Table 3 and Table S5). The addition of acetonitrile has a noticeable but substrate-dependent effect on conversion: in some cases (e.g., linalool, limonene and cyclohexane), conversion increases, whereas in others (e.g., cyclooctene) it remains similar or slightly decreases. This suggests that acetonitrile influences the catalytic system primarily by modifying the reaction medium, potentially improving substrate solubility and accessibility to active sites. It has to be considered that the nature of the copper center might favor radical reaction and thus other species than the ones typically observed in classical epoxidation [66]. Compound **1a** shows consistently high catalytic activity across all investigated substrates, although its selectivity and response to reaction conditions vary, Figure S17, Table 3. In cyclooctene oxidation, the (pre)catalyst achieves moderate-to-high conversion (69–74%) with relatively low epoxide selectivity (20–26%), indicating that although (pre)catalyst activity is high, the oxygen-transfer process is poorly controlled. Nevertheless, the system exhibits high TON values (138–145) and good TOF_50min_, particularly in the presence of acetonitrile, indicating rapid catalytic turnover.

A similar trend is observed in linalool oxidation, where conversions remain high (69–77%), but product selectivity towards furanoid and pyranoid derivatives is low and poorly differentiated. This suggests that the (pre)catalyst promotes non-specific oxidation pathways rather than selective intramolecular cyclisation [67]. Despite this, TON values (136–152) and TOF_50min_ remain relatively high, confirming the system’s strong catalytic efficiency. The addition of acetonitrile slightly improves conversion but does not significantly enhance selectivity, further supporting the idea that ligand structure, rather than solvent effects, governs product distribution.

In limonene oxidation, the **1a** (pre)catalyst exhibits distinct behaviour compared with other substrates. While conversion is somewhat lower under TBHP alone (≈65%), it increases in the presence of acetonitrile (74%). Notably, this system favours allylic oxidation, providing higher selectivity for carvone (7–8%) than typically observed with more structured catalysts. This shift seems to indicate some radical-mediated pathways involving allylic C–H activation. TON and TOF_50min_ values are also significantly enhanced in the presence of acetonitrile (TON up to 149; TOF_50min_ up to 125), suggesting that the solvent may facilitate catalytic turnover in this case. In contrast with what was shown in other works using copper catalysts [68], the selectivity towards carvone and epoxide is similar but carveol and limonene hydroperoxide could not be detected in our case.

The oxidation of cyclohexane further highlights the high activity of this (pre)catalyst in challenging C–H activation reactions. Conversions are moderate to high (≈69–83%), with a relatively balanced distribution between cyclohexanol and cyclohexanone (so-called KA oil [69,70]). However, the (pre)catalyst achieves high TON (133–157) and particularly high TOF_50min_ values (up to 115 h^−1^), demonstrating efficient radical oxidation of an inert substrate. The presence of acetonitrile slightly enhances conversion and TON, although its effect on TOF_50min_ is less pronounced. Overall, the **1a** (pre)catalyst is characterised by high catalytic efficiency across all substrates, as evidenced by consistently elevated TON and TOF values. However, this efficiency is accompanied by relatively low selectivity, with broad product distributions and a tendency toward allylic and non-specific oxidation pathways. These results suggest that the (pre)catalyst operates predominantly via radical mechanisms, with limited ability to control substrate orientation or stabilise specific transition states, leading to efficient but less selective oxidation processes.

The blank control reactions showed only minor substrate conversion for most substrates, with cyclohexane representing the sole notable exception, exhibiting ~30% conversion, without detectable product selectivity (Figure S18). Due to the relatively high conversion in the blank reaction, this reaction was deeply examined. Given this relatively high background conversion, cyclohexane oxidation was investigated further to assess the contribution of individual (pre)catalyst components. Additional control experiments were performed in the presence of the substrate and oxidant replacing **1a** by (i) Cu(NO_3_)_2_, (ii) *N*-methylglycinate (MeGly), (iii) 3,3′-bipyridine (3,3′-bpy), or (iv) a mixture of Cu(NO_3_)_2_, MeGly, and 3,3′-bpy in the corresponding Cu:MeGly:3,3′-bpy molar ratio of 1:1:1. In all cases, results were comparable to those obtained in absence of any (pre)catalyst components, with conversion remaining at approximately 30% and no selectivity observed towards the studied compounds (ESI, Table S6). The corresponding catalytic profiles are provided in the ESI (Figure S19). The calibration curves for all substrates are provided in the ESI (Figure S20).

In contrast, (pre)catalyst **1a** afforded substantially higher conversion of cyclohexane, reaching 69% in the absence of MeCN and increasing to 83% upon addition of MeCN. These results clearly demonstrate that the observed substrate conversion is not attributable to the reaction conditions alone but is dependent on the presence of (pre)catalyst **1a**.

### 3.7. SEM Images of ***1a***

SEM imaging at 10,000× reveals a densely packed microstructure of irregular submicron particles with rod-like morphology. The domains are interconnected and partially agglomerated, producing a rough, highly textured surface. The particle size distribution (hundreds of nanometers to ~1 µm) indicates a heterogeneous but structurally continuous rod-like morphology relevant for surface-dependent polymer performance. Representative SEM images and EDS data are given in Figure S21, revealing the constituents of compound **1a** (Cu, N, O). The elemental composition is given in the inset table.

## 4. Conclusions

This work contributes to the rational design of bio-inspired MOFs and supports their further development as functional materials with tunable structures and catalytic properties. Four new Cu(II)-based bio-inspired MOFs, constructed from 3,3′-bipyridine and *N*-alkyl glycinates, were synthesised by solution-based methods. Compounds **1a** and **2b** were also obtained via mechanochemical synthesis, demonstrating a low-solvent preparation approach. We proposed a supplementary parameter for mechanochemical synthesis of porous compounds to provide a more accurate description of the dynamic nature of synthetic parameters. All compounds were structurally characterised by single-crystal X-ray diffraction, confirming the formation of three-dimensional coordination frameworks and demonstrating that *N*-alkylated amino acids act as versatile ligands. Powder X-ray diffraction confirmed that compound **1a** retains its structural integrity, including stability up to 75% RH, and the gradual transformation at elevated temperatures observed between 50 and 160 °C with the more pronounced phase change at 170 °C. SEM imaging reveals a densely packed microstructure of irregular submicron particles with rod-like morphology. Electron paramagnetic resonance analysis revealed a characteristic anisotropic g-tensor consistent with a Cu(II) centre in a Jahn–Teller-distorted coordination environment, confirming the stability of the local coordination geometry over the investigated temperature range. The results suggest that the MOF acts as a precatalyst under the applied reaction conditions, with the catalytically active species potentially forming through its transformation during the reaction. Thus, the catalytic activity should not necessarily be attributed to the pristine MOF structure, but rather to the active species generated under the reaction conditions.

## Figures and Tables

**Table 1:** The change in *η*-parameter during the reaction and adsorption of liquid reactants. Starting amounts are in relation to the experimental values used in synthesis. Masses and volumes used in the table are the following: 24.4 mg for Cu(OH)_2_, 44.5 mg for MeGly, 60.4 mg for Cu(NO_3_)_2_∙3H_2_O, 39 μL for DMF and 67 μL for 3,3′-bpy.

State of the Reaction	Liquid Composition	*V* (Liquid)/μL	Solids Composition	*m* (Solids)/mg	*η*/μL mg^−1^
initial	3,3′-bpy DMF	106 ^1^	Cu(OH)_2_ Cu(NO_3_)_2_∙3H_2_O MeGly	129	0.822
final	H_2_O	18 ^1^	**1a**	226	0.080

^1^ Volumes were calculated according to density at 25 °C: *ρ*(H_2_O, 25 °C) = 0.99704 g cm^−3^, *ρ*(DMF, 25 °C) = 0.944 g cm^−3^, *ρ*(3,3′-bpy, 25 °C) = 1.16 g cm^−3^.

**Table 2:** The spin-Hamiltonian parameters of simulated ESR spectra for compound **1a**. The uncertainties of all obtained simulation parameters correspond to the last digit, except for the *H*-strain values, where the uncertainty is 10 MHz.

Sample	[*g*_x_ *g*_y_ *g*_z_]	*g*-Strain	*H*-Strain (MHz)	*lw*_pp_ (mT)	*T* (K)
**1a**	[2.060 2.090 2.200]	[0.001 0.008 0.015]	[10 40 70]	2.0	298
			[10 60 70]	1.8	100

**Table 3:** Catalytic performance of **1a** catalyst in oxidation of different substrates. The reactions were carried out at 80 °C for 5 h using TBHP as an oxidant in water, in the absence and presence of acetonitrile as co-solvent. *n*(**1a**)/*n*(substrate)/*n*(oxidant) = 0.1 mmol/20 mmol/40 mmol.

Substrate	Condition	Conversion ^1^ (%)	Selectivity ^2^ (%)	* TON ^3^	* TOF_50_min ^4^ (h^−1^)
**Cyclooctene**	TBHP	74 ± 2	Epoxide: 26 ± 1	145 ± 4	77 ± 2
	TBHP + MeCN	69 ± 2	Epoxide: 20 ± 2	138 ± 4	92 ± 1
**Linalool**	TBHP	69 ± 1	Furanoid: 2 ± 1; Pyranoid: 1 ± 0	136 ± 4	77 ± 2
	TBHP + MeCN	77 ± 2	Furanoid: 4 ± 0; Pyranoid: 3 ± 1	152 ± 6	82 ± 1
**Limonene**	TBHP	65 ± 2	Epoxide: 8 ± 1; Carvone: 8 ± 1	130 ± 4	120 ± 2
	TBHP + MeCN	74 ± 2	Epoxide: 4 ± 1; Carvone: 7 ± 2	149 ± 5	125 ± 2
**Cyclohexane**	TBHP	69 ± 2	Cyclohexanone: 4 ± 1; Cyclohexanol: 6 ± 0	133 ± 5	98 ± 2
	TBHP + MeCN	83 ± 2	Cyclohexanone: 7 ± 1; Cyclohexanol: 8 ± 1	157 ± 9	115 ± 2

^1^ Substrate conversion at the end of reaction (5 h); ^2^ Selectivity toward the main detected products; ^3^ TON = *n*(substrate transformed)/ *n*(**1a**); ^4^ TOF = *n*(substrate transformed)/ *n*(**1a**)/time, calculated at 50 min; * uncertainty in the syringe dispensing of liquid substrates may contribute to minor variations in the calculated TON and TOF values.

## Data Availability

The original contributions presented in this study are included in the article/Supplementary Material. Further inquiries can be directed to the corresponding authors.

## Supplementary Materials

The following supporting information can be downloaded at https://www.mdpi.com/article/10.3390/polym18212698/s1, Figure S1. Retention times: (a) at the beginning of the reaction 3.111—cyclooctene, 7.220—acetophenone; (b) at the end of the reaction 2.061—TBHP, 3.080—cyclooctene, 6.407—cyclooctene oxide, 7.210—acetophenone; Figure S2. Retention times for: (a) 3.601—cyclohexanone, 7.320—acetophenone; (b) 3.755—cyclohexanol, 7.318—acetophenone; (c) the beginning of the reaction 1.217—cyclohexane, 7.325—acetophenone; (d) the end of the reaction 1.215—cyclohexane, 1.405—TBHP, 3.546—cyclohexanone, 3.755—cyclohexanol; Figure S3. Retention times for: (a) 2.983—ethylbenzene, 7.355 and 7.455—pyranoid; (b) 2.980—ethylbenzene, 5.478 and 5.691—furanoid; (c) the beginning of the reaction 2.975—ethylbenzene, 6.108—linalool; (d) the end of the reaction 1.903—TBHP, 2.968—ethylbenzene, 5.476 and 5.692—furanoid, 6.072—linalool, 7.359 and 7.459—pyranoid; Figure S4: Powder X-ray diffraction pattern of the product of mechanochemical synthesis with *N*-methylglycine (blue) and the pattern simulated from the single-crystal structure of **1a** (red); Figure S5. Powder X-ray diffraction pattern of the product of mechanochemical syntheses with *N*-ethylglycine (shades of blue) and the pattern simulated from the single-crystal structure of **2b** (red). Experimental patterns (shades of blue) were measured on three independent synthesis trials, using the same procedure (as reported in the Experimental section of this paper). The quality of the pattern depends on the sample preparation time. Parts of the PXRD pattern calculated from the crystal structure, divided by the broken lines, are not on the same intensity scale; Figure S6: Powder X-ray diffraction pattern of the product of mechanochemical syntheses with *N*-ethylglycine measured immediately after synthesis and after certain periods of time (purple and shades of blue), and the pattern simulated from the single-crystal structure of **2b** (pink). Parts of the PXRD pattern divided by the broken lines are not on the same intensity scale. Yellow lines represent diffraction maxima attributed to **2b**, while blue and red lines show newly observed phases; Figure S7: Atom numbering scheme for **1a** and **2a**. DMF and water molecules were omitted for clarity; Figure S8. Intraframework hydrogen bonds (cyan dotted lines) in **1a** and **2a**; Figure S9: Atom numbering scheme for **1b** and **2b**; Figure S10: Intraframework hydrogen bonds (cyan dotted lines) and four-atom cluster of copper(II) atoms in **1b** and **2b**; Figure S11: IR spectrum of {[Cu(μ-MeGly)(NO_3_)(μ-3,3′-bpy)]·DMF·0.5H_2_O}*_n_* (**1a**); Figure S12: TGA (black) and DSC (blue) curves of {[Cu(μ-MeGly)(NO_3_)(μ-3,3′-bpy)]·DMF·0.5H_2_O}*_n_* (**1a**); Figure S13: Gram–Schmidt profile (a) and TGA-FTIR three-dimensional plot (b) of {[Cu(μ-MeGly)(NO_3_)(μ-3,3′-bpy)]·DMF·0.5H_2_O}*_n_* (**1a**); Figure S14. TGA curves of mechanochemically prepared {[Cu(μ-MeGly)(NO_3_)(μ-3,3′-bpy)]·DMF·0.5H_2_O}*_n_* (**1a**). The blue line represents a moist sample, measured immediately after synthesis, while the red line represents a sample of the same synthesis dried at ambient conditions; Figure S15. Kinetic profiles for cyclooctene (C8), linalool (LIN), limonene (LIMO) and cyclohexane (C6) conversion with error bars. A full dot represents the reaction without the addition of acetonitrile, while the empty dot represents the reaction with the addition of acetonitrile; Figure S16. Kinetic profiles for: (a) cyclooctene (C8), (b) linalool (LIN), (c) limonene (LIMO), (d) cyclohexane (C6) conversion with error bars. A full dot represents the reaction without the addition of acetonitrile, while the empty dot represents the reaction with the addition of acetonitrile; Figure S17. Product selectivity in the oxidation of cyclooctene (blue), linalool (green), limonene (purple), and cyclohexane (red) using TBHP, with and without acetonitrile. Full bars represent reactions without addition of acetonitrile and empty bars represent reactions with addition of acetonitrile; Figure S18. Kinetic profile for oxidation reaction of cyclohexane without the catalyst; Figure S19: Kinetic profile for oxidation of cyclohexane with starting compounds; Figure S18: Calibration curves for substrates and products; Figure S20. Calibration curves for (a) cyclohexane, (b) cyclohexanone, (c) cyclohexanol, (d) cyclooctene, (e) cyclooctene oxide, (f) limonene, (g) limonene oxide, (h) carvone, (i) carveol, (j) diol, (k) linalool, (l) furanoid, (m) pyranoid; Figure S21. SEM images and the EDS analysis of the mechanochemically prepared sample of **1a**; Table S1: Water, phosphorus(V) oxide, and various salts whose saturated solutions were used to achieve specific relative humidities of the atmosphere at 20 °C; Table S2: Crystallographic data for compounds **1a**, **1b**, **2a** and **2b**; Table S3: Distances ([Å]) and bond angles ([°]) within the polyhedra of copper coordination spheres, in the crystal structures of **1a**, **1b**, **2a** and **2b**; Table S4: Geometry of selected intra- and intermolecular hydrogen bonds ([Å],[°]) for compounds **1a, 1b, 2a** and **2b**; Table S5. Catalytic performance and product yields for the oxidation of different substrates using TBHP in the presence and absence of MeCN. Table S6: Conversion and product selectivity for the oxidation of cyclohexane with starting compounds. CCDC 2583579-2583582 contain the supplementary crystallographic data for this paper. These data can be obtained free of charge via https://www.ccdc.cam.ac.uk/structures/ (accessed on 17 September 2026) or from the CCDC, 12 Union Road, Cambridge CB2 1EZ, UK; Fax: +44 1223 336033; E-mail: deposit@ccdc.cam.ac.uk.
