# Supplementary material for: 3,3′-Bipyridine and N-Alkyl Glycinates as Building Blocks for Cu-Based Bio-Inspired MOFs
Source: Polymers (Basel). 2026 Oct 31;18(21):2698. doi: 10.3390/polym18212698 (PMC13765576; doi:10.3390/polym18212698)

checkCIF/PLATON report

Structure factors have been supplied for datablock(s) 1a

THIS REPORT IS FOR GUIDANCE ONLY. IF USED AS PART OF A REVIEW PROCEDURE FOR PUBLICATION, IT SHOULD NOT REPLACE THE EXPERTISE OF AN EXPERIENCED CRYSTALLOGRAPHIC REFEREE.

No syntax errors found. CIF dictionary Interpreting this report

Datablock: 1a

```
Bond precision:    C-C = 0.0069 A           Wavelength=1.54184

Cell:               a=20.3111(4)      b=23.5653(5)      c=8.3630(2)
                    alpha=90          beta=90           gamma=90
Temperature:        170 K
```

	Calculated	Reported
Volume	4002.84(15)	4002.84(15)
Space group	C c c 2	C c c 2
Hall group	C 2 -2c	C 2 -2c
Moiety formula	2(C13 H14 Cu N4 O5), 2(C3 H7 N O), H2 O	2(C13 H14 Cu N4 O5), 2(C3 H7 N O), H2 O
Sum formula	C32 H44 Cu2 N10 O13	C32 H44 Cu2 N10 O13
Mr	903.87	903.85
Dx,g cm-3	1.500	1.500
Z	4	4
Mu (mm-1)	1.951	1.951
F000	1872.0	1872.0
F000'	1861.95	
h,k,lmax	24,28,10	24,28,10
Nref	3609[1947]	3281
Tmin,Tmax	0.921,0.943	0.408,1.000
Tmin'	0.768	

```
Correction method= # Reported T Limits: Tmin=0.408 Tmax=1.000
AbsCorr = MULTI-SCAN
```

Data completeness= 1.69/0.91 Theta(max)= 67.480

R(reflections)= 0.0419(3137) wR2(reflections)= 0.1183(3281)

S = 1.078 Npar= 258

The following ALERTS were generated. Each ALERT has the format
test-name_ALERT_alert-type_alert-level.
Click on the hyperlinks for more details of the test.

Alert level B

PLAT420_ALERT_2_B D-H Bond Without Acceptor O1W --H1W1 . Please Check

Author Response: 'Water molecule is disordered, but it forms hydrogen bonds with neighbouring DMF molecules.'

Alert level C

PLAT090_ALERT_3_C Poor Data / Parameter Ratio (Zmax > 18) .... 7.49 Note
PLAT202_ALERT_3_C Isotropic non-H Atoms in Anion/Solvent .... 1 Check
O1W
PLAT213_ALERT_2_C Atom O14N has ADP max/min Ratio ..... 3.5 prolat
PLAT213_ALERT_2_C Atom O15N has ADP max/min Ratio ..... 3.5 prolat
PLAT220_ALERT_2_C NonSolvent Resd 1 O Ueq(max)/Ueq(min) Range 4.5 Ratio
PLAT242_ALERT_2_C Low MainResAtom Ueq as Compared to Neighbours N1N Check
PLAT244_ALERT_4_C Low MinorResAtom Ueq as Compared to Neighbours N1D Check
PLAT244_ALERT_4_C Low MinorResAtom Ueq as Compared to Neighbours C1D Check
PLAT250_ALERT_2_C Large U3/U1 Ratio for <U(i,j)> Tensor(Resd 1) 2.3 Note
PLAT260_ALERT_2_C Large Average Ueq of Residue Including O1D 0.173 Check
PLAT341_ALERT_3_C Low Bond Precision on C-C Bonds .... 0.00691 Ang.
PLAT911_ALERT_3_C Missing FCF Refl Between Thmin & STh/L= 0.599 13 Report
2 0 0, 4 0 0, 6 0 0, 8 0 0, 10 0 0, 12 0 0,
12 24 0, 10 0 2, 12 0 2, 2 2 2, 0 0 4, 0 24 4,
0 0 10,

Alert level G

PLAT002_ALERT_2_G Number of Distance or Angle Restraints on AtSite 2 Note
PLAT004_ALERT_5_G Polymeric Structure Found with Maximum Dimension 3 Info
PLAT007_ALERT_5_G Number of Unrefined Donor-H Atoms .... 1 Report
H1
PLAT160_ALERT_4_G max(sig)/min(sig) ratio x,y,z > 3 for CU1 8.87 Check
PLAT171_ALERT_4_G The CIF-Embedded .res File Contains EADP Records 2 Report
PLAT172_ALERT_4_G The CIF-Embedded .res File Contains DFIX Records 1 Report
PLAT232_ALERT_2_G Hirshfeld Test Diff (M-X) Cu1 --O12_e . 7.4 s.u.
PLAT301_ALERT_3_G Main Residue Disorder ....(Resd 1) 9% Note
PLAT380_ALERT_4_G Incorrectly? Oriented X(sp2)-Methyl Moiety ..... C2D Check
PLAT380_ALERT_4_G Incorrectly? Oriented X(sp2)-Methyl Moiety ..... C3D Check
PLAT720_ALERT_4_G Number of Unusual/Non-Standard Labels .... 7 Note
H2D1 H2D2 H2D3 H3D1 H3D2 H3D3 H1W1

```
PLAT860_ALERT_3_G Number of Least-Squares Restraints .............          2 Note
PLAT883_ALERT_1_G Absent Datum for _atom_sites_solution_primary ..     Please Do !
PLAT899_ALERT_4_G SHELXL2018 is Outdated   and Succeeded by SHELXL     2019/3 Note
PLAT909_ALERT_3_G Percentage of I>2sig(I) Data at Theta(Max) Still        87% Note
PLAT910_ALERT_3_G Missing FCF Reflection(s) Below Theta(Min)[Deg]=       3.75 Note
                1  1  0,
PLAT913_ALERT_3_G Missing # of Very Strong Reflections in FCF ....          1 Note
                2  0  0,
PLAT933_ALERT_2_G Number of HKL-OMIT Records in Embedded .res File          9 Note
                0 26 -2,   4 24 -3,   0 16 -8,   2  2 -2,  15 21 -4,  17 21 -2,
               13 21  5,   2  0  0,   7 23  5,
PLAT965_ALERT_2_G The SHELXL WEIGHT Optimisation has not Converged     Please Check
PLAT967_ALERT_5_G Note: Two-Theta Cutoff Value in Embedded .res ..      135.0 Degree
PLAT969_ALERT_5_G The 'Henn et al.' R-Factor-gap value ..........      2.808 Note
              Predicted wR2: Based on SigI**2  4.21 or SHELX Weight 10.98 Note
PLAT978_ALERT_2_G Number C-C Bonds with Positive Residual Density.          2 Info
```

```
   0 ALERT level A = Most likely a serious problem - resolve or explain
   1 ALERT level B = A potentially serious problem, consider carefully
  12 ALERT level C = Check. Ensure it is not caused by an omission or oversight
  22 ALERT level G = General information/check it is not something unexpected

   1 ALERT type 1 CIF construction/syntax error, inconsistent or missing data
  12 ALERT type 2 Indicator that the structure model may be wrong or deficient
   9 ALERT type 3 Indicator that the structure quality may be low
   9 ALERT type 4 Improvement, methodology, query or suggestion
   4 ALERT type 5 Informative message, check
```

It is advisable to attempt to resolve as many as possible of the alerts in all categories. Often the minor alerts point to easily fixed oversights, errors and omissions in your CIF or refinement strategy, so attention to these fine details can be worthwhile. It is up to the individual to critically assess their own results and, if necessary, seek expert advice.

PLATON version of 20/06/2026; check.def file version of 19/06/2026

---

duplicate check

No duplication found

Datablock 1a - ellipsoid plot

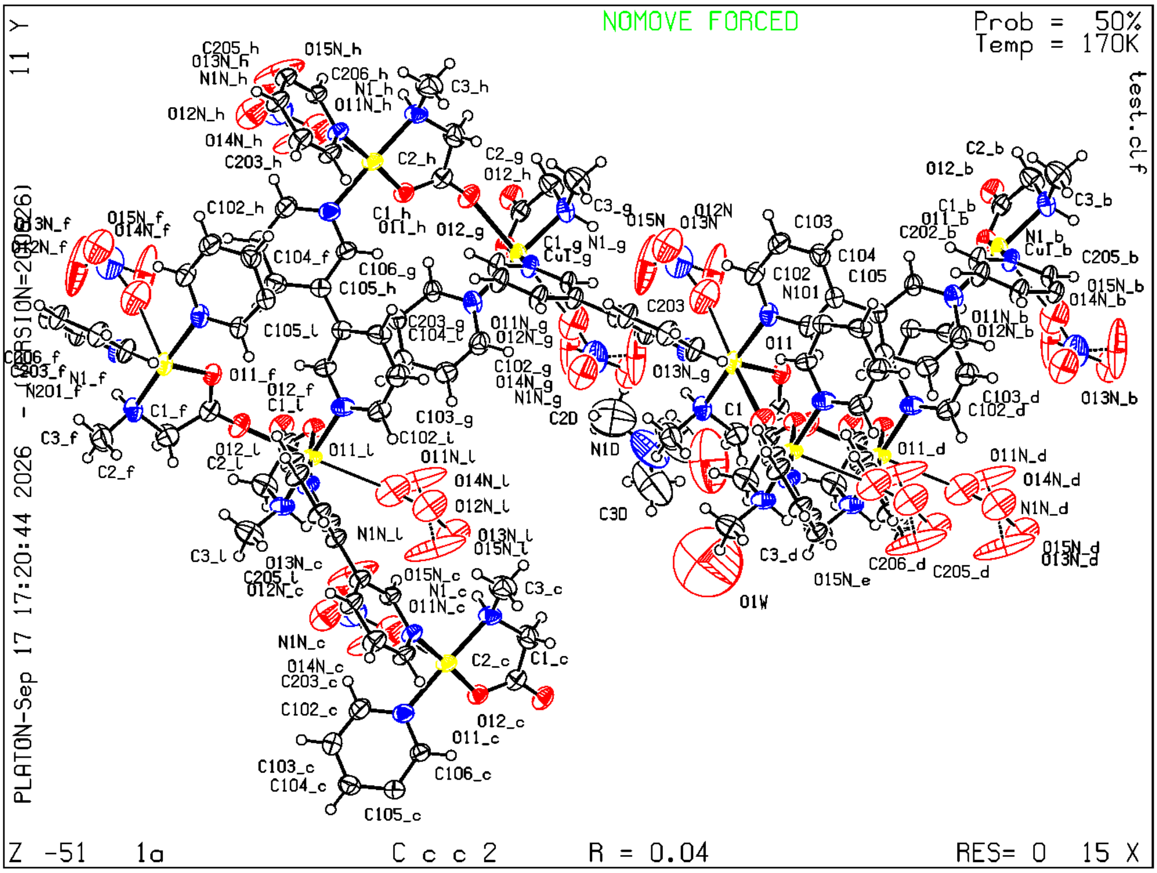

Supplement: Supplementary file 1 [file polymers-18-02698-s001.zip › 1a_checkcif.pdf]
