# Supplementary material for: 3,3′-Bipyridine and N-Alkyl Glycinates as Building Blocks for Cu-Based Bio-Inspired MOFs
Source: Polymers (Basel). 2026 Oct 31;18(21):2698. doi: 10.3390/polym18212698 (PMC13765576; doi:10.3390/polym18212698)

checkCIF/PLATON report

Structure factors have been supplied for datablock(s) 1b

THIS REPORT IS FOR GUIDANCE ONLY. IF USED AS PART OF A REVIEW PROCEDURE FOR PUBLICATION, IT SHOULD NOT REPLACE THE EXPERTISE OF AN EXPERIENCED CRYSTALLOGRAPHIC REFEREE.

No syntax errors found. CIF dictionary Interpreting this report

Datablock: 1b

Bond precision:	C-C = 0.0068 A		Wavelength=1.54184
Cell:	a=43.2960(4)	b=43.2960(4)	c=43.2960(4)
	alpha=90	beta=90	gamma=90
Temperature:	170 K		

	Calculated	Reported
Volume	81160(2)	81160(2)
Space group	I a -3 d	I a -3 d
Hall group	-I 4bd 2c 3	-I 4bd 2c 3
Moiety formula	C13 H14 Cu N4 O5 [+ solvent]	C13 H14 Cu N4 O5 [+ solvent]
Sum formula	C13 H14 Cu N4 O5 [+ solvent]	C13 H14 Cu N4 O5
Mr	369.83	369.82
Dx,g cm-3	0.726	0.726
Z	96	96
Mu (mm-1)	1.048	1.048
F000	18144.0	18144.0
F000’	18009.00	
h,k,lmax	54,54,54	52,53,46
Nref	6983	6794
Tmin,Tmax	0.802,0.811	0.256,1.000
Tmin’	0.802	

```
Correction method= # Reported T Limits: Tmin=0.256 Tmax=1.000
AbsCorr = MULTI-SCAN

Data completeness= 0.973          Theta(max)= 74.970

R(reflections)= 0.0664( 3187)                 wR2(reflections)=
                                              0.2716( 6794)
S = 1.066                  Npar= 208
```

The following ALERTS were generated. Each ALERT has the format
test-name_ALERT_alert-type_alert-level.
Click on the hyperlinks for more details of the test.

Alert level B

```
PLAT242_ALERT_2_B Low   MainResAtom  Ueq as Compared to Neighbours       O11N Check
```

Author Response: ’O11N is part of disordered nitrate ion.’

Alert level C

```
PLAT026_ALERT_3_C Ratio Observed / Unique Reflections (too) Low ..        47% Check
PLAT084_ALERT_3_C High wR2 Value (i.e. > 0.25) ..................       0.27 Report
PLAT220_ALERT_2_C NonSolvent   Resd 1  N   Ueq(max)/Ueq(min) Range        3.4 Ratio
PLAT220_ALERT_2_C NonSolvent   Resd 1  O   Ueq(max)/Ueq(min) Range        5.2 Ratio
PLAT241_ALERT_2_C High  MainResAtom  Ueq as Compared to Neighbours        N1N Check
PLAT241_ALERT_2_C High  MainResAtom  Ueq as Compared to Neighbours       C103 Check
PLAT242_ALERT_2_C Low   MainResAtom  Ueq as Compared to Neighbours        Cu1 Check
```

Author Response: ’O11N is part of disordered nitrate ion.’

```
PLAT260_ALERT_2_C Large Average Ueq of Residue Including       Cu1      0.145 Check
PLAT341_ALERT_3_C Low Bond Precision on  C-C Bonds ..............     0.0068 Ang.
PLAT905_ALERT_3_C Negative K value in the Analysis of Variance ...    -31.640 Report
PLAT905_ALERT_3_C Negative K value in the Analysis of Variance ...     -3.810 Report
PLAT905_ALERT_3_C Negative K value in the Analysis of Variance ...     -0.391 Report
PLAT911_ALERT_3_C Missing FCF Refl Between Thmin & STh/L=    0.600         35 Report
                0  0  4,   1  2  5,   3  4  5,   0  2  6,   5  5  6,   3  4  7,
                3  6  7,   2  2  8,   2  7  9,   3  8  9,   4  4 12,   2  3 21,
                0  0 28,   1  1 30,   0  2 30,   7 35 36,   9 35 36,   0 36 36,
                4 36 36,   8 36 36,   2 35 37,   4 35 37,   6 35 37,   8 35 37,
               10 35 37,   1 36 37,   3 36 37,   5 36 37,   8 34 38,   1 35 38,
                   (    5 More NOT listed: see .ckf listing file)
PLAT934_ALERT_3_C Number of (Iobs-Icalc)/Sigma(W) > 10 Outliers ..          1 Check
                0  0 12,
```

Alert level G

```
PLAT004_ALERT_5_G Polymeric Structure Found with Maximum Dimension          3 Info
```

```
PLAT007_ALERT_5_G Number of Unrefined Donor-H Atoms .............          1 Report
              H1
PLAT180_ALERT_4_G Check Cell Rounding: # of Values Ending with 0 =          3 Note
PLAT606_ALERT_4_G Solvent Accessible VOID(S) in  Crystal Structure          ! Info
PLAT794_ALERT_5_G Tentative Bond Valency for Cu1       (II)      .       2.18 Info
PLAT869_ALERT_4_G ALERTS Related to the Use of SQUEEZE  Suppressed          ! Info
PLAT883_ALERT_1_G Absent Datum for _atom_sites_solution_primary ..     Please Do !
PLAT899_ALERT_4_G SHELXL2018 is Outdated   and Succeeded by SHELXL     2019/3 Note
PLAT910_ALERT_3_G Missing FCF Reflection(s) Below Theta(Min)[Deg]=       2.89 Note
                1  1  2,
PLAT912_ALERT_4_G Missing # of FCF Reflections Above STh/L=  0.600        145 Note
PLAT913_ALERT_3_G Missing # of Very Strong Reflections in FCF ....          2 Note
                1  1  2,   0  0  4,
PLAT933_ALERT_2_G Number of HKL-OMIT Records in Embedded .res File         11 Note
                3  4  7,   2  3 21,   1  2  5,   3  6  7,   2  2  8,   2  7  9,
                5  5  6,   3  8  9,   0  2  6,   4  4 12,   3  4  5,
PLAT941_ALERT_3_G Average HKL Measurement Multiplicity ..........         4.9 Low
PLAT950_ALERT_5_G Calculated (ThMax) and CIF-Reported Hmax  Differ          2 Units
PLAT952_ALERT_5_G Calculated (ThMax) and CIF-Reported Lmax  Differ          8 Units
PLAT955_ALERT_1_G Reported (CIF) and Actual (FCF) Lmax Differ by .          7 Units
PLAT960_ALERT_3_G Number of Intensities with I < - 2*Sigma(I) ....         28 Check
PLAT965_ALERT_2_G The SHELXL WEIGHT Optimisation has not Converged     Please Check
PLAT967_ALERT_5_G Note: Two-Theta Cutoff Value in Embedded .res ..      150.0 Degree
PLAT969_ALERT_5_G The 'Henn et al.' R-Factor-gap value ..........      8.892 Note
              Predicted wR2: Based on SigI**2  3.05 or SHELX Weight 25.65 Note
PLAT978_ALERT_2_G Number C-C Bonds with Positive Residual Density.          0 Info
```

```
  0 ALERT level A = Most likely a serious problem - resolve or explain
  1 ALERT level B = A potentially serious problem, consider carefully
 14 ALERT level C = Check. Ensure it is not caused by an omission or oversight
 21 ALERT level G = General information/check it is not something unexpected

  2 ALERT type 1 CIF construction/syntax error, inconsistent or missing data
 10 ALERT type 2 Indicator that the structure model may be wrong or deficient
 12 ALERT type 3 Indicator that the structure quality may be low
  5 ALERT type 4 Improvement, methodology, query or suggestion
  7 ALERT type 5 Informative message, check
```

It is advisable to attempt to resolve as many as possible of the alerts in all categories. Often the minor alerts point to easily fixed oversights, errors and omissions in your CIF or refinement strategy, so attention to these fine details can be worthwhile. It is up to the individual to critically assess their own results and, if necessary, seek expert advice.

PLATON version of 20/06/2026; check.def file version of 19/06/2026

duplicate check

No duplication found

Datablock 1b - ellipsoid plot

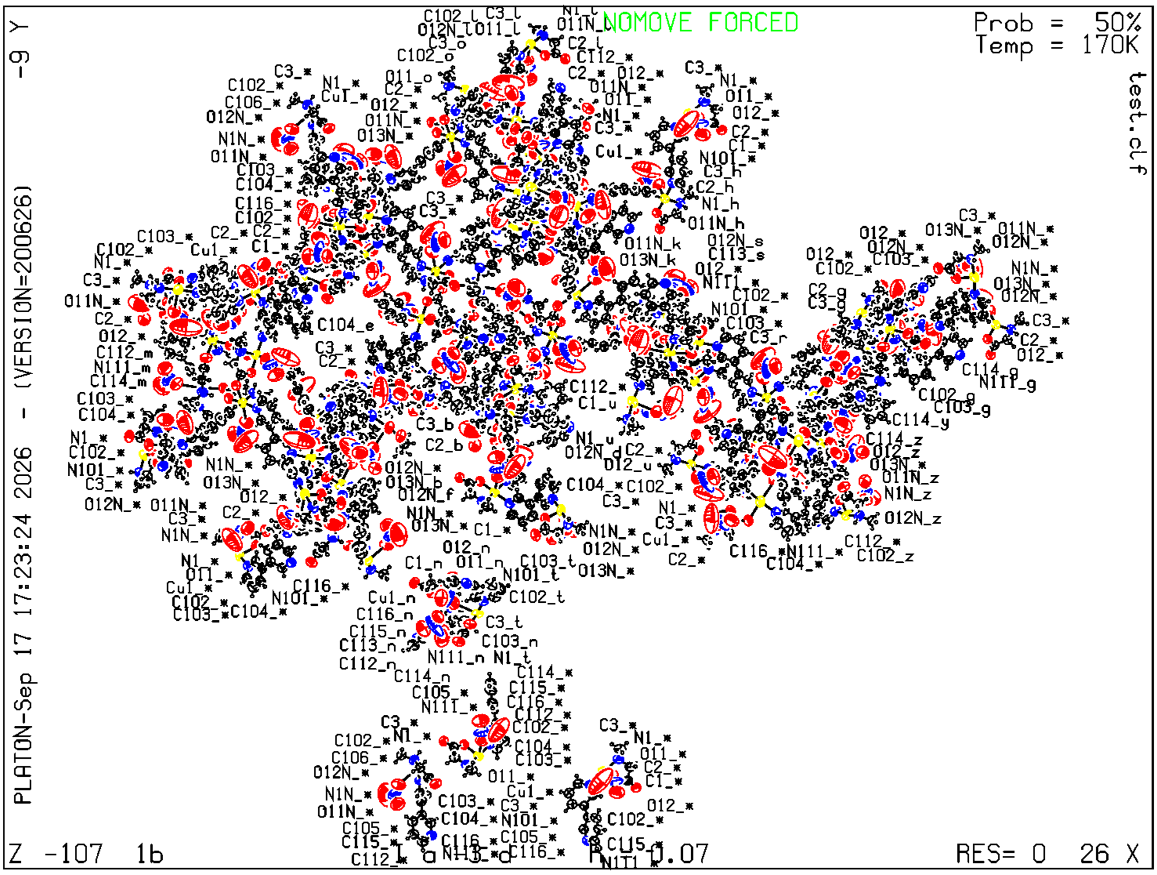

Supplement: Supplementary file 1 [file polymers-18-02698-s001.zip › 1b_checkcif.pdf]
