# Supplementary material for: 3,3′-Bipyridine and N-Alkyl Glycinates as Building Blocks for Cu-Based Bio-Inspired MOFs
Source: Polymers (Basel). 2026 Oct 31;18(21):2698. doi: 10.3390/polym18212698 (PMC13765576; doi:10.3390/polym18212698)

checkCIF/PLATON report

Structure factors have been supplied for datablock(s) 2a

THIS REPORT IS FOR GUIDANCE ONLY. IF USED AS PART OF A REVIEW PROCEDURE FOR PUBLICATION, IT SHOULD NOT REPLACE THE EXPERTISE OF AN EXPERIENCED CRYSTALLOGRAPHIC REFEREE.

No syntax errors found.   CIF dictionary   Interpreting this report

Datablock: 2a

Bond precision:	C-C = 0.0138 A		Wavelength=1.54184
Cell:	a=21.0807(7)	b=23.3609(7)	c=8.3945(2)
	alpha=90	beta=90	gamma=90
Temperature:	170 K		

	Calculated	Reported
Volume	4134.0(2)	4134.0(2)
Space group	C c c 2	C c c 2
Hall group	C 2 -2c	C 2 -2c
Moiety formula	2(C14 H16 Cu N4 O5), 2(C3 H7 N O), H2 O	2(C14 H16 Cu N4 O5), 2(C3 H7 N O), H2 O
Sum formula	C34 H48 Cu2 N10 O13	C34 H48 Cu2 N10 O13
Mr	931.92	931.90
Dx,g cm-3	1.497	1.497
Z	4	4
Mu (mm-1)	1.907	1.907
F000	1936.0	1936.0
F000'	1926.09	
h,k,lmax	25,27,10	26,29,10
Nref	3726[2007]	3712
Tmin,Tmax	0.852,0.875	0.450,1.000
Tmin'	0.523	

Correction method= # Reported T Limits: Tmin=0.450 Tmax=1.000
AbsCorr = MULTI-SCAN

Data completeness= 1.85/1.00   Theta(max)= 67.465

R(reflections)= 0.0565(3634)   wR2(reflections)= 0.1615(3712)

S = 1.078   Npar= 224

The following ALERTS were generated. Each ALERT has the format
test-name_ALERT_alert-type_alert-level.
Click on the hyperlinks for more details of the test.

Alert level B

```
PLAT412_ALERT_2_B Short Intra XH3 .. XHn     H12A     ..H13A     .       1.75 Ang.
                                                       x,y,z  =      1_555 Check
```

Author Response: 'Methyl group hydrogen atoms in disordered DMF molecule.'

```
PLAT420_ALERT_2_B D-H Bond Without Acceptor  O1W      --H1W      .     Please Check
```

Author Response: 'Water molecule is disordered, but it forms hydrogen bonds with neighbouring DMF molecules.'

Alert level C

```
PLAT160_ALERT_4_C max(sig)/min(sig) ratio x,y,z > 3 for CU1          22.76 Check
PLAT202_ALERT_3_C Isotropic non-H Atoms in Anion/Solvent .........       6 Check
              O1D     N11D    C11D    C12D    C13D    O1W
PLAT220_ALERT_2_C NonSolvent   Resd 1  O   Ueq(max)/Ueq(min) Range       3.1 Ratio
PLAT241_ALERT_2_C High  MainResAtom  Ueq as Compared to Neighbours      O11N Check
PLAT241_ALERT_2_C High  MainResAtom  Ueq as Compared to Neighbours        C2 Check
PLAT242_ALERT_2_C Low   MainResAtom  Ueq as Compared to Neighbours        N1 Check
PLAT242_ALERT_2_C Low   MainResAtom  Ueq as Compared to Neighbours       N1N Check
PLAT243_ALERT_4_C High MinorResAtom  Ueq as Compared to Neighbours      C11D Check
PLAT244_ALERT_4_C Low  MinorResAtom  Ueq as Compared to Neighbours      N11D Check
PLAT250_ALERT_2_C Large U3/U1 Ratio for <U(i,j)> Tensor(Resd    1)       2.2 Note
PLAT309_ALERT_2_C Single Bonded Oxygen (C-O > 1.3 Ang) ..........       O1D Check
PLAT341_ALERT_3_C Low Bond Precision on  C-C Bonds ..............    0.01383 Ang.
PLAT413_ALERT_2_C Short Inter XH3 .. XHn     H12A     ..H13B     .       2.09 Ang.
                                                 -x,y,-1/2+z  =      4_554 Check
PLAT911_ALERT_3_C Missing FCF Refl Between Thmin & STh/L=    0.599          7 Report
                2  0  0,   4  0  0,   0  2  0,   2  2  0,   1  7  0,   3  3  1,
                1  5  2,
PLAT975_ALERT_2_C Check Calcd Resid. Dens.  0.81Ang From O1D     .       0.44 eA-3
```

Alert level G

```
PLAT002_ALERT_2_G Number of Distance or Angle Restraints on AtSite          2 Note
PLAT004_ALERT_5_G Polymeric Structure Found with Maximum Dimension          3 Info
PLAT007_ALERT_5_G Number of Unrefined Donor-H Atoms .............          2 Report
              H1      H1W
```

```
PLAT083_ALERT_2_G SHELXL Second Parameter in WGHT  Unusually Large       5.02 Why ?
PLAT160_ALERT_4_G max(sig)/min(sig) ratio x,y,z > 3 for C205             3.59 Check
PLAT160_ALERT_4_G max(sig)/min(sig) ratio x,y,z > 3 for N101             3.59 Check
PLAT160_ALERT_4_G max(sig)/min(sig) ratio x,y,z > 3 for N201             3.59 Check
PLAT160_ALERT_4_G max(sig)/min(sig) ratio x,y,z > 3 for O11              4.02 Check
PLAT160_ALERT_4_G max(sig)/min(sig) ratio x,y,z > 3 for O12              3.98 Check
PLAT172_ALERT_4_G The CIF-Embedded .res File Contains DFIX Records          1 Report
PLAT232_ALERT_2_G Hirshfeld Test Diff (M-X)  Cu1      --O12_e    .        6.5 s.u.
PLAT380_ALERT_4_G Incorrectly? Oriented X(sp2)-Methyl Moiety .....       C12D Check
PLAT380_ALERT_4_G Incorrectly? Oriented X(sp2)-Methyl Moiety .....       C13D Check
PLAT794_ALERT_5_G Tentative Bond Valency for Cu1       (II)      .       2.11 Info
PLAT883_ALERT_1_G Absent Datum for _atom_sites_solution_primary ..     Please Do !
PLAT899_ALERT_4_G SHELXL2018 is Outdated   and Succeeded by SHELXL     2019/3 Note
PLAT909_ALERT_3_G Percentage of I>2sig(I) Data at Theta(Max) Still        92% Note
PLAT913_ALERT_3_G Missing # of Very Strong Reflections in FCF ....          1 Note
                2  0  0,
PLAT933_ALERT_2_G Number of HKL-OMIT Records in Embedded .res File         11 Note
               20 10 -6,   1  5  2,   1  5 -2,   1  7  0,   3  3  1,   0  2  0,
                2  2  0,   3  3 -1,   4  0  0,   2  0  0,   4  2 -1,
PLAT941_ALERT_3_G Average HKL Measurement Multiplicity ..........         1.9 Low
PLAT951_ALERT_5_G Calculated (ThMax) and CIF-Reported Kmax  Differ         -2 Units
PLAT953_ALERT_1_G Reported (CIF) and Actual (FCF) Hmax Differ by .          1 Units
PLAT954_ALERT_1_G Reported (CIF) and Actual (FCF) Kmax Differ by .          2 Units
PLAT965_ALERT_2_G The SHELXL WEIGHT Optimisation has not Converged     Please Check
PLAT967_ALERT_5_G Note: Two-Theta Cutoff Value in Embedded .res ..      135.0 Degree
PLAT969_ALERT_5_G The 'Henn et al.' R-Factor-gap value ..........      7.496 Note
              Predicted wR2: Based on SigI**2  2.15 or SHELX Weight 14.98 Note
PLAT978_ALERT_2_G Number C-C Bonds with Positive Residual Density.          2 Info
```

---

0 **ALERT level A** = Most likely a serious problem - resolve or explain
2 **ALERT level B** = A potentially serious problem, consider carefully
15 **ALERT level C** = Check. Ensure it is not caused by an omission or oversight
27 **ALERT level G** = General information/check it is not something unexpected

3 ALERT type 1 CIF construction/syntax error, inconsistent or missing data
17 ALERT type 2 Indicator that the structure model may be wrong or deficient
6 ALERT type 3 Indicator that the structure quality may be low
12 ALERT type 4 Improvement, methodology, query or suggestion
6 ALERT type 5 Informative message, check

It is advisable to attempt to resolve as many as possible of the alerts in all categories. Often the minor alerts point to easily fixed oversights, errors and omissions in your CIF or refinement strategy, so attention to these fine details can be worthwhile. It is up to the individual to critically assess their own results and, if necessary, seek expert advice.

PLATON version of 20/06/2026; check.def file version of 19/06/2026

duplicate check

No duplication found

Datablock 2a - ellipsoid plot

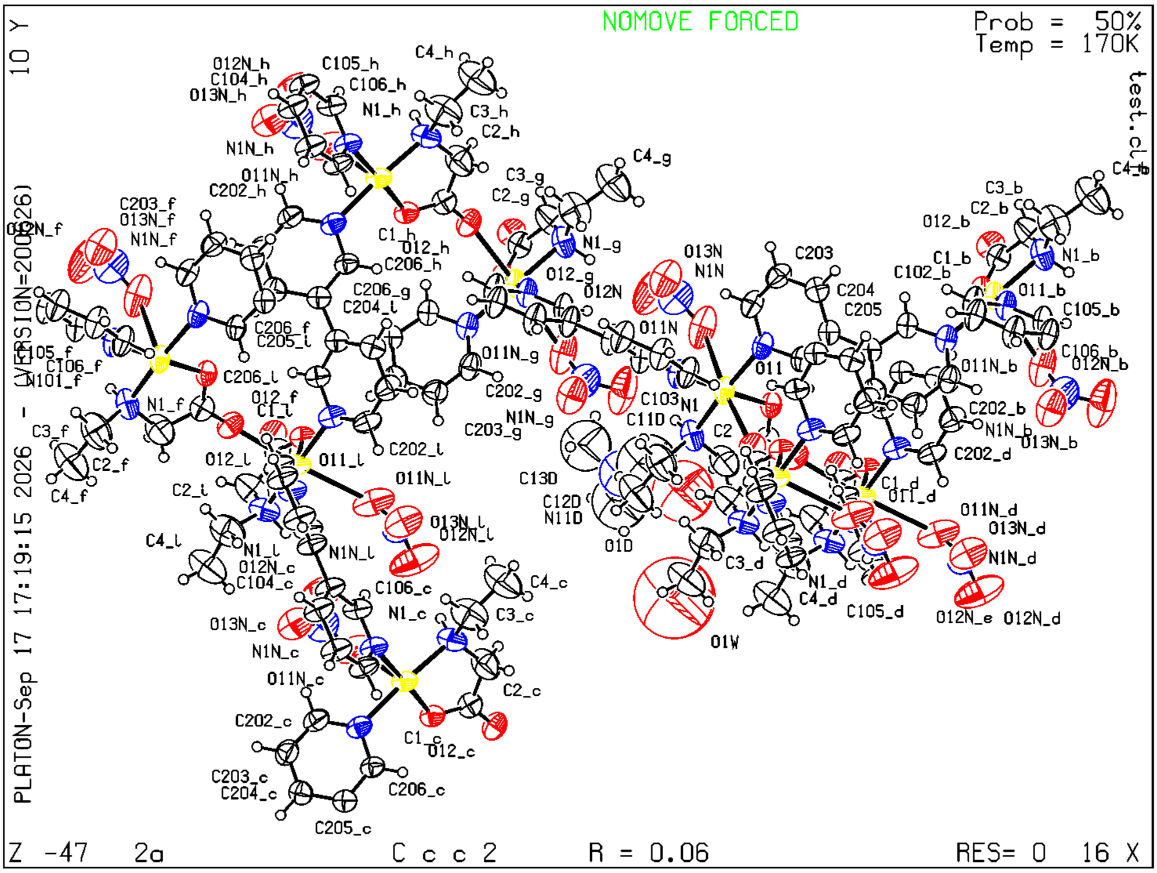

Supplement: Supplementary file 1 [file polymers-18-02698-s001.zip › 2a_checkcif.pdf]
