# Supplementary material for: 3,3′-Bipyridine and N-Alkyl Glycinates as Building Blocks for Cu-Based Bio-Inspired MOFs
Source: Polymers (Basel). 2026 Oct 31;18(21):2698. doi: 10.3390/polym18212698 (PMC13765576; doi:10.3390/polym18212698)

checkCIF/PLATON report

Structure factors have been supplied for datablock(s) 2b

THIS REPORT IS FOR GUIDANCE ONLY. IF USED AS PART OF A REVIEW PROCEDURE FOR PUBLICATION, IT SHOULD NOT REPLACE THE EXPERTISE OF AN EXPERIENCED CRYSTALLOGRAPHIC REFEREE.

No syntax errors found.   CIF dictionary   Interpreting this report

Datablock: 2b

Bond precision:	C-C = 0.0035 A		Wavelength=1.54184
Cell:	a=43.6280(1)	b=43.6280(1)	c=43.6280(1)
	alpha=90	beta=90	gamma=90
Temperature:	170 K		

	Calculated	Reported
Volume	83041.6(6)	83041.6(6)
Space group	I a -3 d	I a -3 d
Hall group	-I 4bd 2c 3	-I 4bd 2c 3
Moiety formula	C14 H16 Cu N4 O5 [+ solvent]	C14 H16 Cu N4 O5 [+ solvent]
Sum formula	C14 H16 Cu N4 O5 [+ solvent]	C14 H16 Cu N4 O5
Mr	383.86	383.85
Dx,g cm-3	0.737	0.737
Z	96	96
Mu (mm-1)	1.035	1.035
F000	18912.0	18912.0
F000’	18778.64	
h,k,lmax	53,53,53	51,45,48
Nref	6581	6366
Tmin,Tmax	0.723,0.752	0.711,1.000
Tmin’	0.656	

Correction method= # Reported T Limits: Tmin=0.711 Tmax=1.000
AbsCorr = MULTI-SCAN

Data completeness= 0.967   Theta(max)= 69.999

R(reflections)= 0.0475(5636)   wR2(reflections)= 0.1644(6366)

S = 1.065   Npar= 217

The following ALERTS were generated. Each ALERT has the format
test-name_ALERT_alert-type_alert-level.
Click on the hyperlinks for more details of the test.

Alert level C

```
PLAT241_ALERT_2_C High   MainResAtom  Ueq as Compared to Neighbours       O11N Check
PLAT242_ALERT_2_C Low    MainResAtom  Ueq as Compared to Neighbours        Cu1 Check
PLAT242_ALERT_2_C Low    MainResAtom  Ueq as Compared to Neighbours        N1N Check
PLAT905_ALERT_3_C Negative K value in the Analysis of Variance ...      -0.508 Report
PLAT911_ALERT_3_C Missing FCF Refl Between Thmin & STh/L=    0.600          79 Report
                1  2  5,   1  4  7,   2  6  8,   1  3 10,   3  7 10,   7  7 10,
                4  7 11,   2  6 12,   3 12 13,   1  1 14,   1  5 14,   7 10 15,
               14 14 16,   4  8 20,   4 18 20,   1 12 23,   2 22 30,  29 30 31,
               26 31 33,  25 32 33,  27 29 34,  26 30 34,  20 34 34,  27 28 35,
               20 33 35,  21 31 36,  20 32 36,   0 36 36,  20 31 37,  21 29 38,
                   (   49 More NOT listed: see .ckf listing file)
PLAT918_ALERT_3_C Reflection(s) with I(obs) much Smaller I(calc) .          28 Check
                2  3  3,   2  4  6,   1  5  6,   3  4  7,   2  2  8,   3  6  9,
                4  7  9,   5  6 11,   5  8 11,   4  9 11,   5 10 11,   6 11 11,
                4  4 12,   1  5 12,   1  7 12,   6  8 12,   3 11 12,   4  9 13,
                1  3 14,   3  8 15,   2  9 15,   0  0 16,   4  4 16,   2  6 18,
                   (    4 More NOT listed: see .ckf listing file)
```

Alert level G

```
PLAT004_ALERT_5_G Polymeric Structure Found with Maximum Dimension           3 Info
PLAT007_ALERT_5_G Number of Unrefined Donor-H Atoms ..............           1 Report
              H1
PLAT083_ALERT_2_G SHELXL Second Parameter in WGHT  Unusually Large      120.67 Why ?
PLAT606_ALERT_4_G Solvent Accessible VOID(S) in  Crystal Structure           ! Info
PLAT794_ALERT_5_G Tentative Bond Valency for Cu1       (II)      .        2.11 Info
PLAT869_ALERT_4_G ALERTS Related to the Use of SQUEEZE  Suppressed           ! Info
PLAT883_ALERT_1_G Absent Datum for _atom_sites_solution_primary ..      Please Do !
PLAT899_ALERT_4_G SHELXL2018 is Outdated   and Succeeded by SHELXL      2019/3 Note
PLAT912_ALERT_4_G Missing # of FCF Reflections Above STh/L=  0.600         105 Note
PLAT933_ALERT_2_G Number of HKL-OMIT Records in Embedded .res File          17 Note
                1  2  5,   4 18 20,   1  5 14,   1  4  7,   3  7 10,   2  6 12,
                1  3 10,   1 12 23,   7 10 15,   3 12 13,   1  1 14,  14 14 16,
                4  7 11,   2 22 30,   4  8 20,   2  6  8,   7  7 10,
PLAT950_ALERT_5_G Calculated (ThMax) and CIF-Reported Hmax  Differer          2 Units
PLAT951_ALERT_5_G Calculated (ThMax) and CIF-Reported Kmax  Differ           8 Units
PLAT952_ALERT_5_G Calculated (ThMax) and CIF-Reported Lmax  Differ           5 Units
PLAT955_ALERT_1_G Reported (CIF) and Actual (FCF) Lmax Differ by .           3 Units
```

```
PLAT958_ALERT_1_G Calculated (ThMax) and Actual (FCF) Lmax  Differ          2 Units
PLAT960_ALERT_3_G Number of Intensities with I < - 2*Sigma(I) ....         53 Check
PLAT965_ALERT_2_G The SHELXL WEIGHT Optimisation has not Converged     Please Check
PLAT967_ALERT_5_G Note: Two-Theta Cutoff Value in Embedded .res ..      140.0 Degree
PLAT969_ALERT_5_G The 'Henn et al.' R-Factor-gap value ..........     21.886 Note
              Predicted wR2: Based on SigI**2  0.75 or SHELX Weight 15.73 Note
PLAT978_ALERT_2_G Number C-C Bonds with Positive Residual Density.          3 Info
```

```
   0 ALERT level A = Most likely a serious problem - resolve or explain
   0 ALERT level B = A potentially serious problem, consider carefully
   6 ALERT level C = Check. Ensure it is not caused by an omission or oversight
  20 ALERT level G = General information/check it is not something unexpected

   3 ALERT type 1 CIF construction/syntax error, inconsistent or missing data
   7 ALERT type 2 Indicator that the structure model may be wrong or deficient
   4 ALERT type 3 Indicator that the structure quality may be low
   4 ALERT type 4 Improvement, methodology, query or suggestion
   8 ALERT type 5 Informative message, check
```

It is advisable to attempt to resolve as many as possible of the alerts in all categories. Often the minor alerts point to easily fixed oversights, errors and omissions in your CIF or refinement strategy, so attention to these fine details can be worthwhile. It is up to the individual to critically assess their own results and, if necessary, seek expert advice.

PLATON version of 20/06/2026; check.def file version of 19/06/2026

---

duplicate check

No duplication found

Datablock 2b - ellipsoid plot

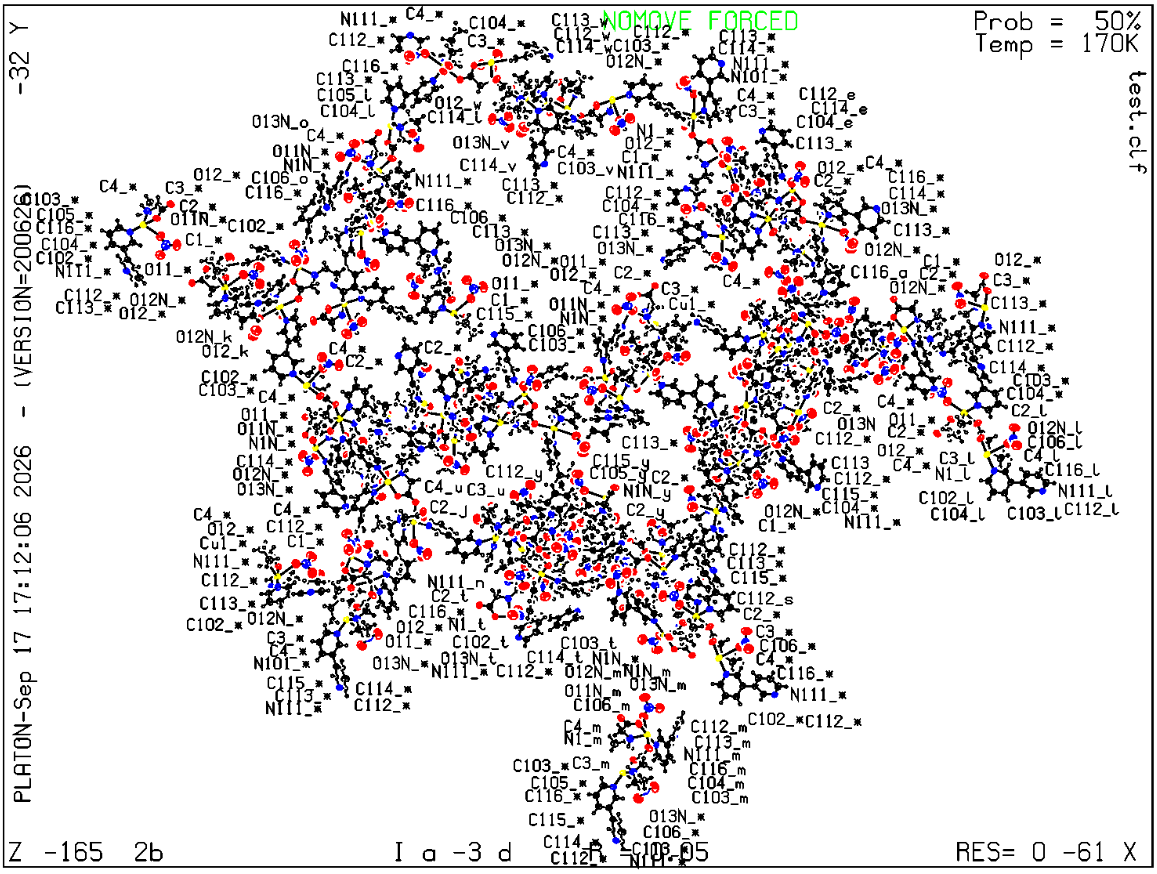

Supplement: Supplementary file 1 [file polymers-18-02698-s001.zip › 2b_checkcif.pdf]
