# Supplementary material for: 3,3′-Bipyridine and N-Alkyl Glycinates as Building Blocks for Cu-Based Bio-Inspired MOFs
Source: Polymers (Basel). 2026 Oct 31;18(21):2698. doi: 10.3390/polym18212698 (PMC13765576; doi:10.3390/polym18212698)
Supplement: Supplementary file 1 [file polymers-18-02698-s001.zip › polymers-4603531-supplementary-english.pdf]

ESI

3,3′-Bipyridine and *N*-Alkyl Glycinates as Building Blocks for Cu-Based Bio-Inspired MOFs

Darko Vušak [1], Mia Jurković [1,2], Katarina Ležaić [1,3], David Kučera-Čavara [4], Dijana Žilić [4], Željka Soldin [1], Dominique Agustin [5,6], Josipa Sarjanović [1], Jana Pisk [1] and Biserka Prugovečki [1,*]

1 University of Zagreb Faculty of Science, Department of Chemistry, Horvatovac 102a, 10000 Zagreb, Croatia; darko.vusak@chem.pmf.unizg.hr (D.V.); m.jurkovic@utwente.nl (M.J.); k.lezaic@uu.nl (K.L.); zeljka.soldin@chem.pmf.unizg.hr (Ž.S.); josipa.sarjanovic@chem.pmf.unizg.hr (J.S.); jana.pisk@chem.pmf.unizg.hr (J.P.)

2 Department of Membrane Science and Technology, MESA+ Institute of Nanotechnology, Faculty of Science and Technology, University of Twente, P.O. Box 217, 7500 AE Enschede, The Netherlands

3 Institute for Sustainable and Circular Chemistry, Faculty of Science, Utrecht University, Universiteitsweg 99, 3584CG Utrecht, The Netherlands

4 Laboratory for Magnetic Resonances, Division of Physical Chemistry, Ruđer Bošković Institute,
Bijeničkacesta 54, 10000 Zagreb, Croatia; dakuceracavara@irb.hr (D.K.-Č.); dzilic@irb.hr (D.Ž.)

5 LCC-CNRS (Laboratoire de Chimie de Coordination), 205 Route de Narbonne, BP44099, CEDEX 4,
31077 Toulouse, France; dominique.agustin@iut-tlse3.fr

6 Department of Chemistry, IUT Toulouse, University of Toulouse, 5 Allée du Martinet, 81100 Castres, France

* Correspondence: biserka.prugovecki@chem.pmf.unizg.hr

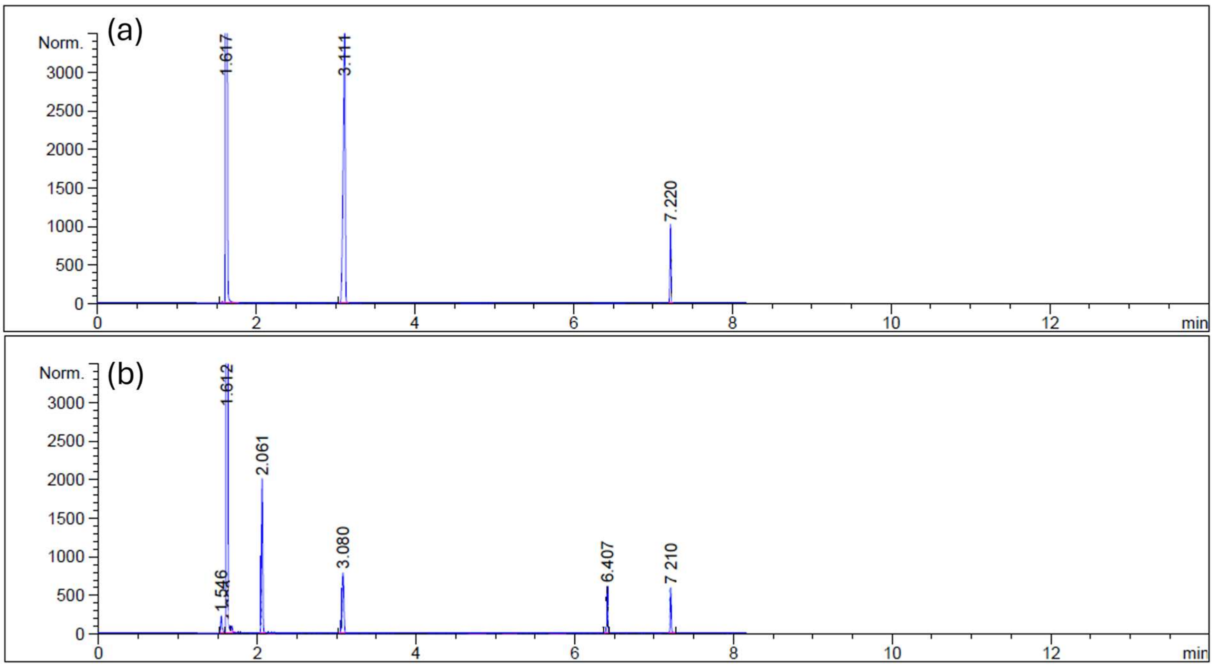

Figure S1. Retention times: (a) at the beginning of the reaction 3.111 – cyclooctene, 7.220 – acetophenone; (b) at the end of the reaction 2.061 – TBHP, 3.080 – cyclooctene, 6.407 – cyclooctene oxide, 7.210 – acetophenone.

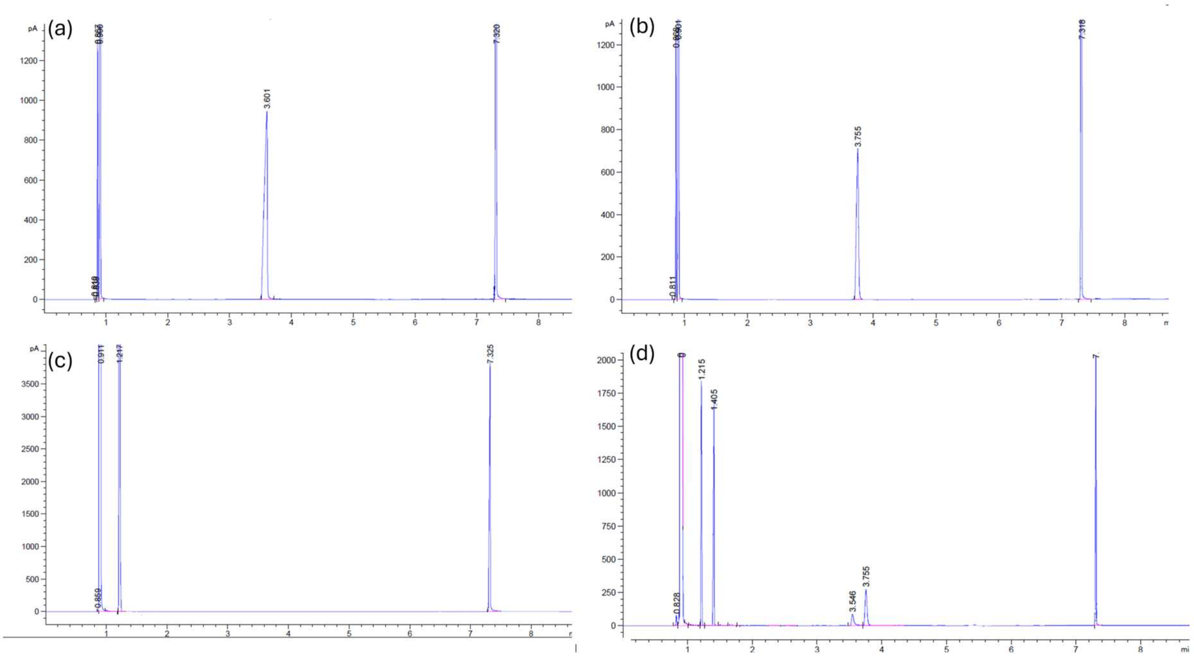

Figure S2. Retention times for: (a) 3.601 – cyclohexanone, 7.320 – acetophenone; (b) 3.755 – cyclohexanol, 7.318 – acetophenone; (c) the beginning of the reaction 1.217 – cyclohexane, 7.325 – acetophenone; (d) the end of the reaction 1.215 – cyclohexane, 1.405 – TBHP, 3.546 – cyclohexanone, 3.755 – cyclohexanol.

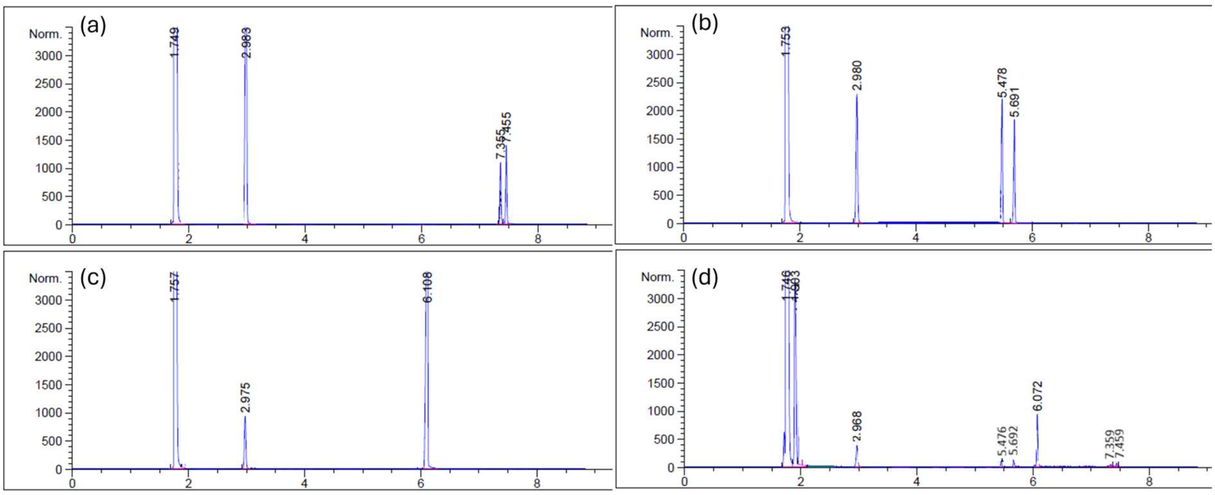

Figure S3. Retention times for: (a) 2.983 – ethylbenzene, 7.355 and 7.455 – pyranoid; (b) 2.980 – ethylbenzene, 5.478 and 5.691 – furanoid; (c) the beginning of the reaction 2.975 – ethylbenzene, 6.108 – linalool; (d) the end of the reaction 1.903 – TBHP, 2.968 – ethylbenzene, 5.476 and 5.692 – furanoid, 6.072 – linalool, 7.359 and 7.459 – pyranoid.

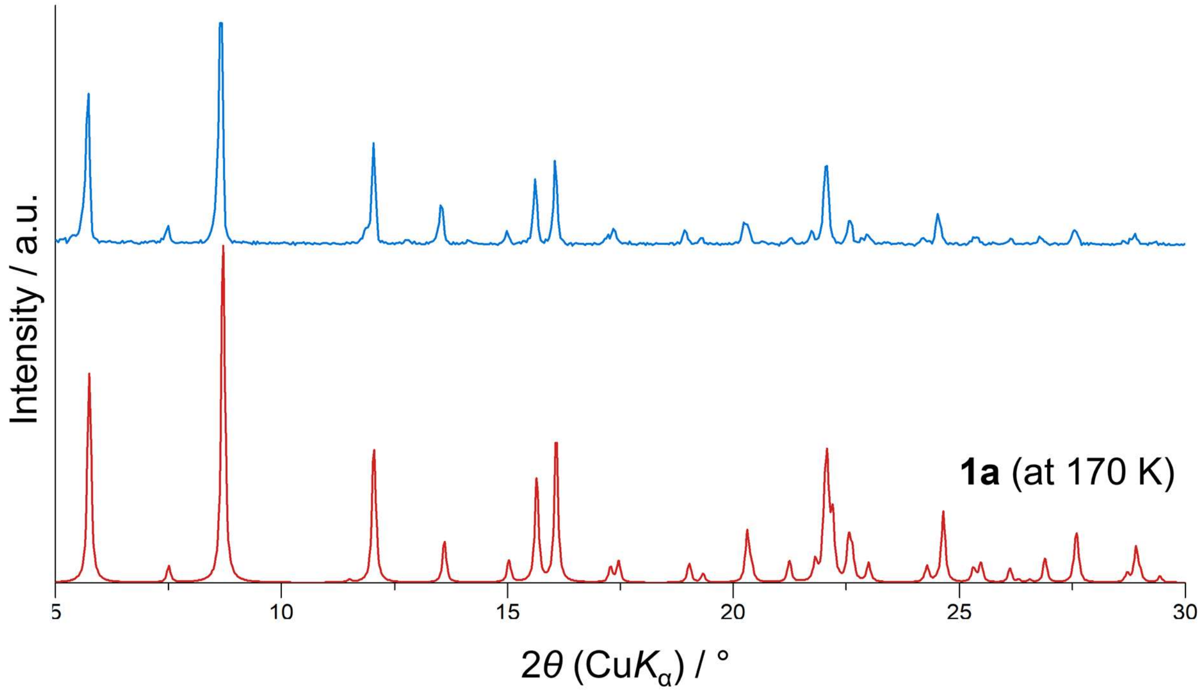

Figure S4. Powder X-ray diffraction pattern of the product of mechanochemical synthesis with *N*-methylglycine (blue) and the pattern simulated from the single-crystal structure of **1a** (red).

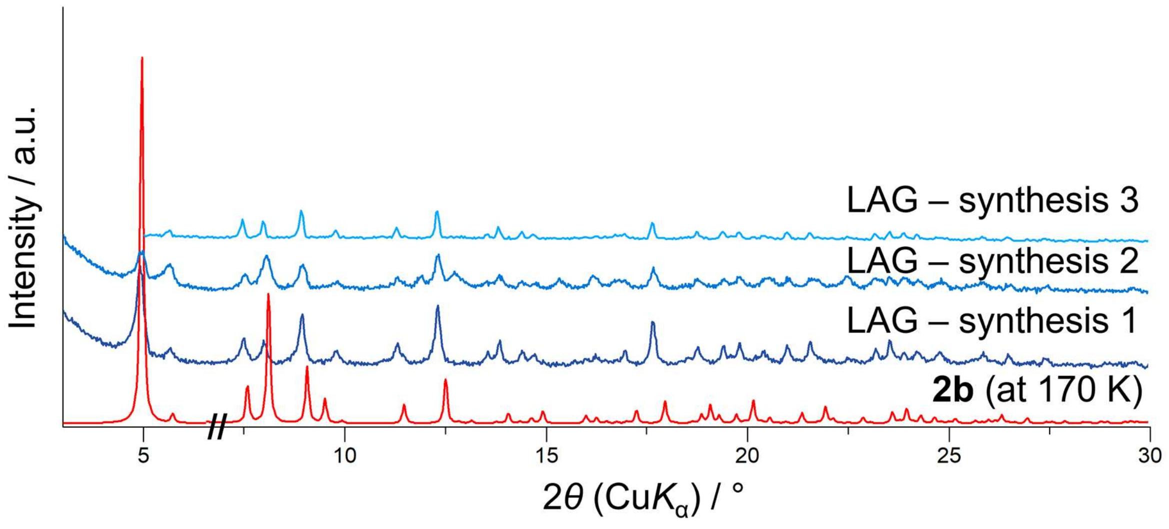

Figure S5. Powder X-ray diffraction pattern of the product of mechanochemical syntheses with *N*-ethylglycine (shades of blue) and the pattern simulated from the single-crystal structure of **2b** (red). Experimental patterns (shades of blue) were measured on three independent synthesis trials, using the same procedure (as reported in the Experimental section of this paper). The quality of the pattern depends on the sample preparation time. Parts of the PXRD pattern calculated from the crystal structure, divided by the broken lines, are not on the same intensity scale.

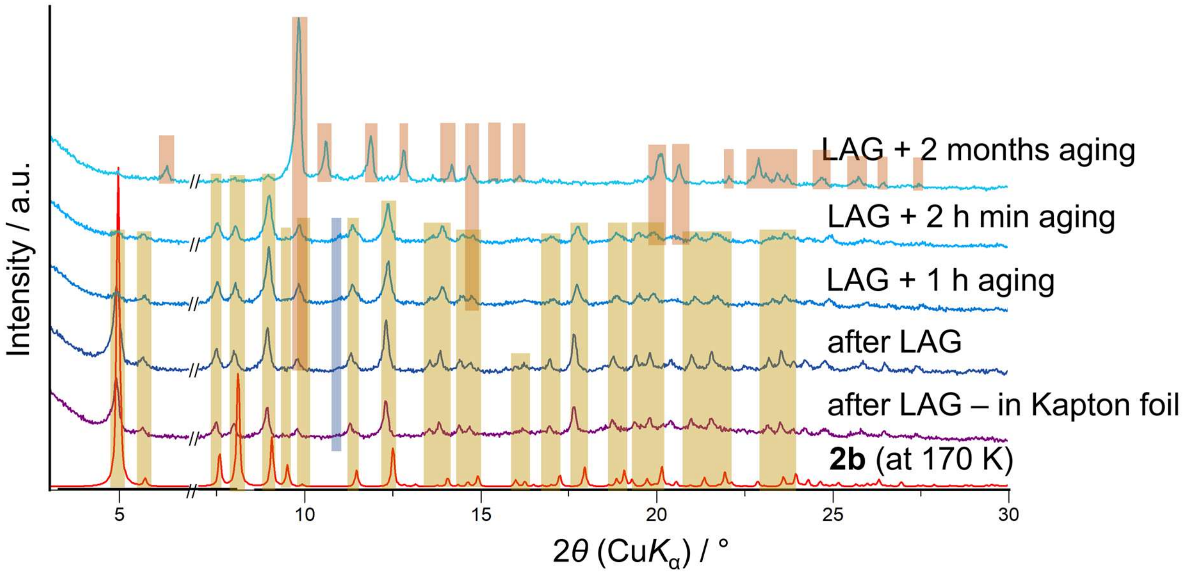

Figure S6. Powder X-ray diffraction pattern of the product of mechanochemical syntheses with *N*-ethylglycine measured immediately after synthesis and after certain periods of time (purple and shades of blue), and the pattern simulated from the single-crystal structure of **2b** (pink). Parts of the PXRD pattern divided by the broken lines are not on the same intensity scale. Yellow lines represent diffraction maxima attributed to **2b**, while blue and red lines show newly observed phases.

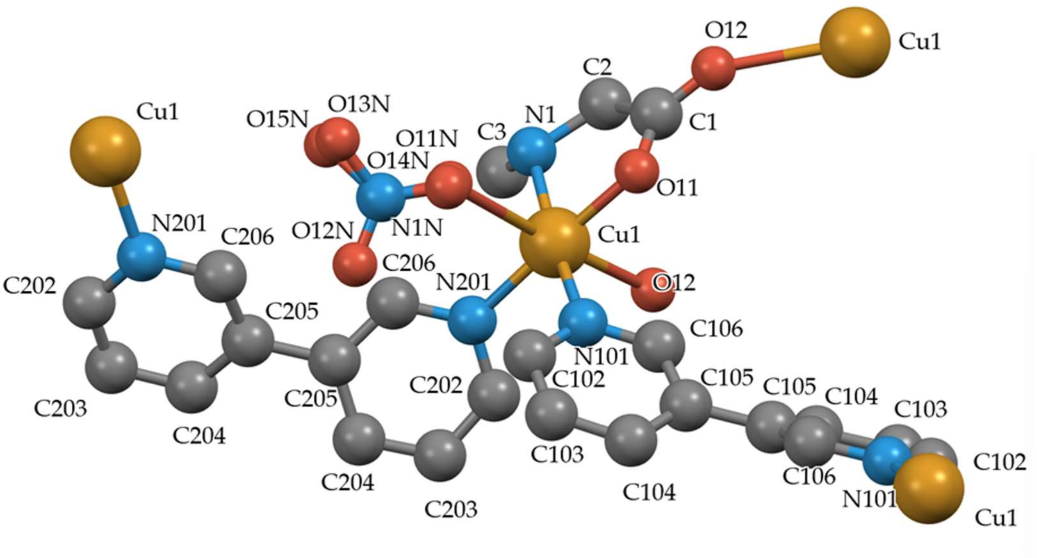

1a

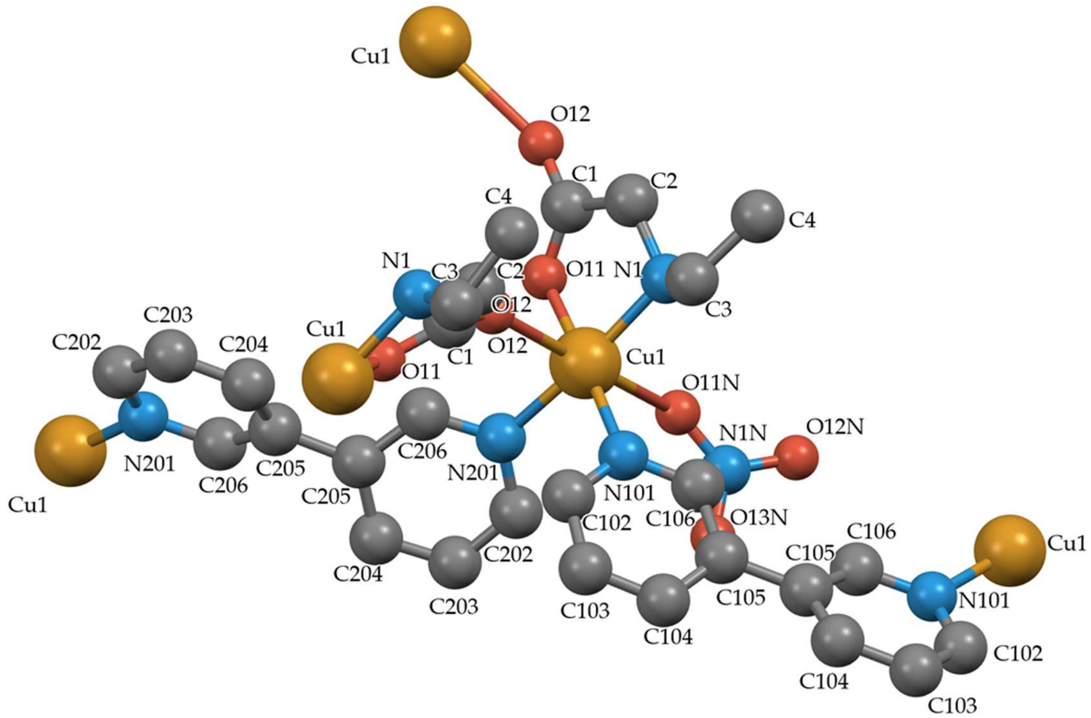

2a

Figure S7. Atom numbering scheme for **1a** and **2a**. DMF and water molecules were omitted for clarity.

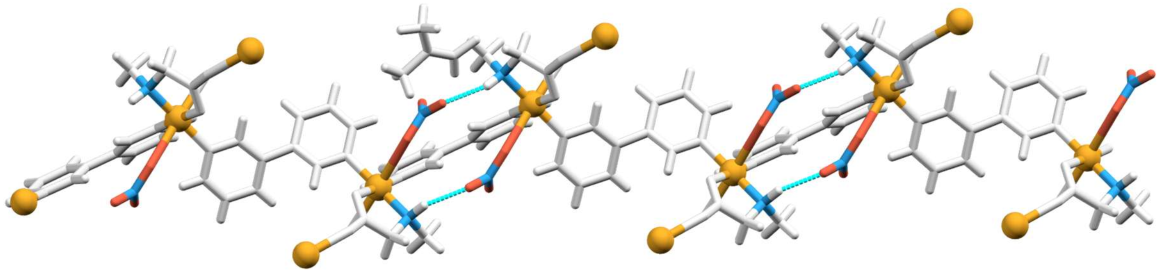

1a

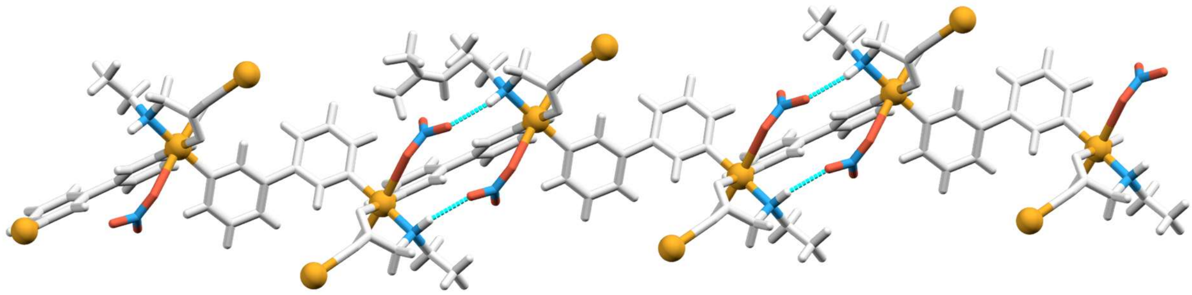

2a

Figure S8. Intraframework hydrogen bonds (cyan dotted lines) in **1a** and **2a**.

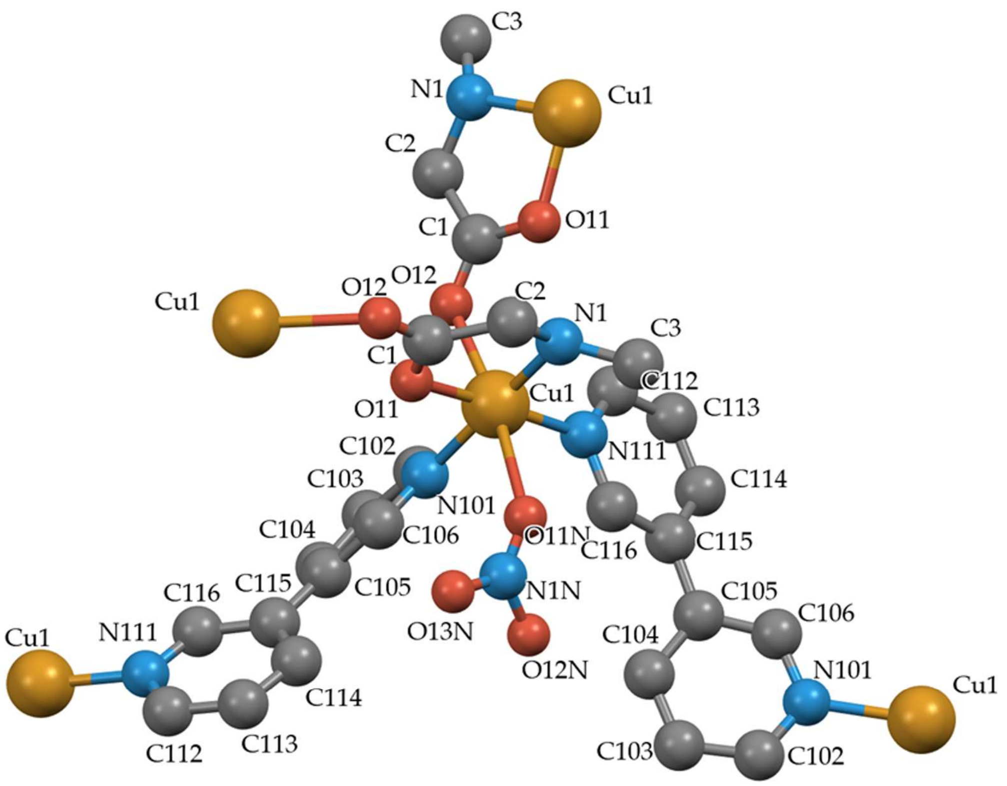

1b

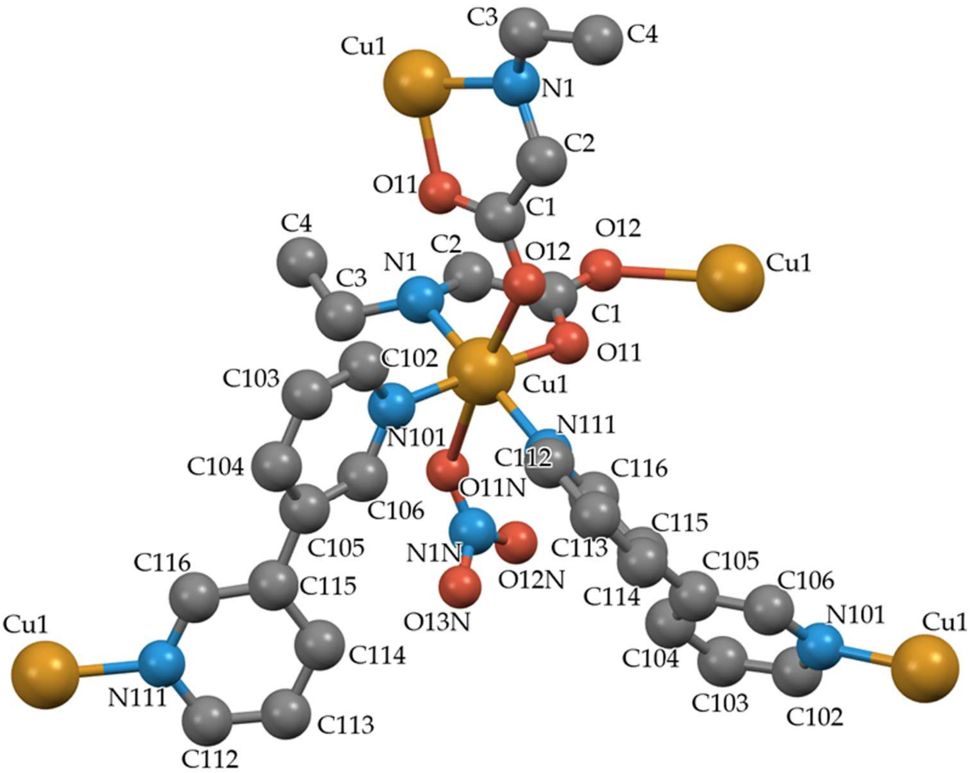

2b

Figure S9. Atom numbering scheme for **1b** and **2b**.

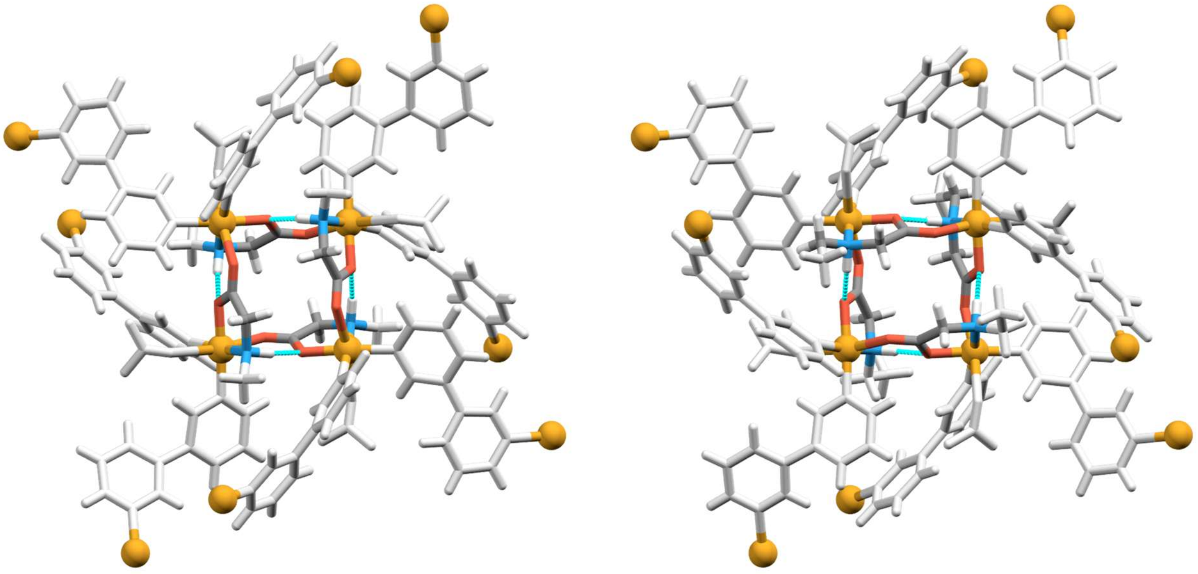

1b **2b**

Figure S10. Intraframework hydrogen bonds (cyan dotted lines) and four-atom cluster of copper(II) atoms in **1b** and **2b**.

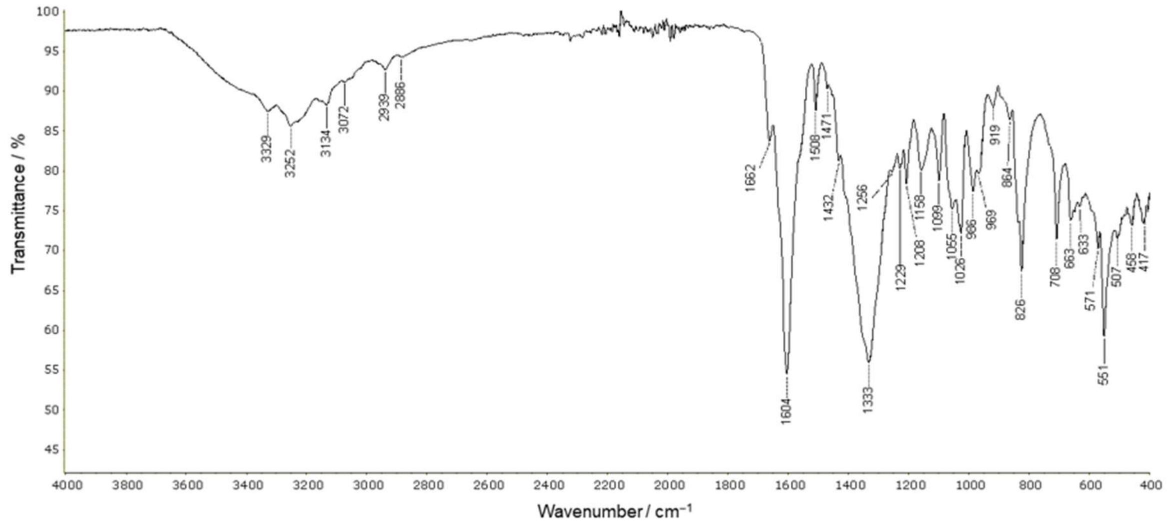

Figure S11. IR spectrum of {[Cu(μ-MeGly)(NO_3)(μ-3,3′-bpy)]·DMF·0.5H_2O}$_n$ (**1a**).

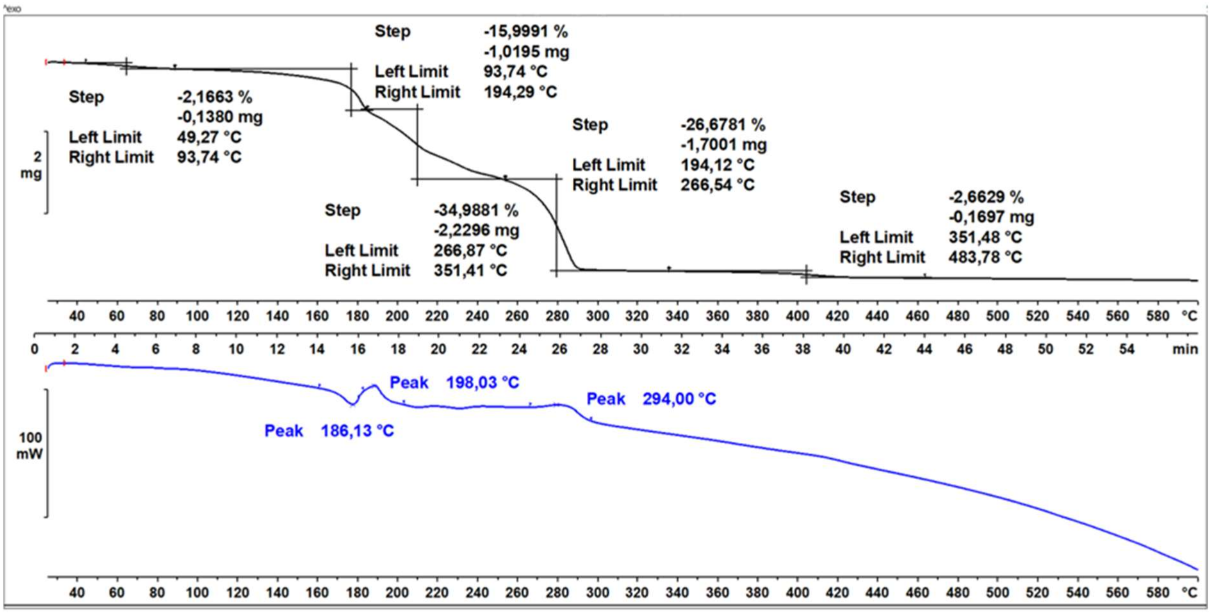

Figure S12. TGA (black) and DSC (blue) curves of {[Cu(μ-MeGly)(NO_3)(μ-3,3′-bpy)]·DMF·0.5H_2O}$_n$ (**1a**).

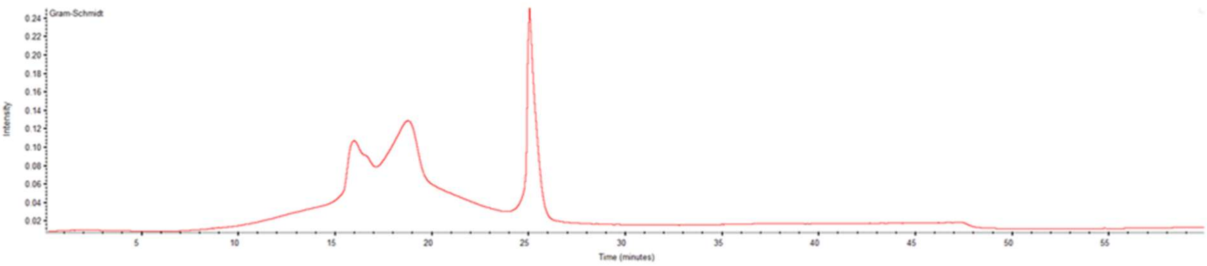

a)

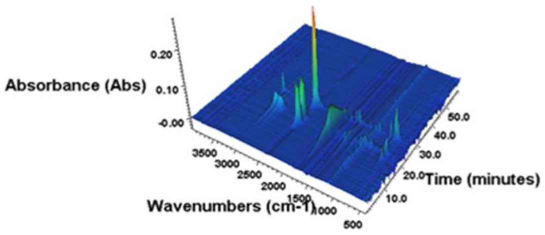

b)

Figure S13. Gram–Schmidt profile (a) and TGA-FTIR three-dimensional plot (b) of {[Cu(μ-MeGly)(NO_3)(μ-3,3′-bpy)]·DMF·0.5H_2O}$_n$ (**1a**).

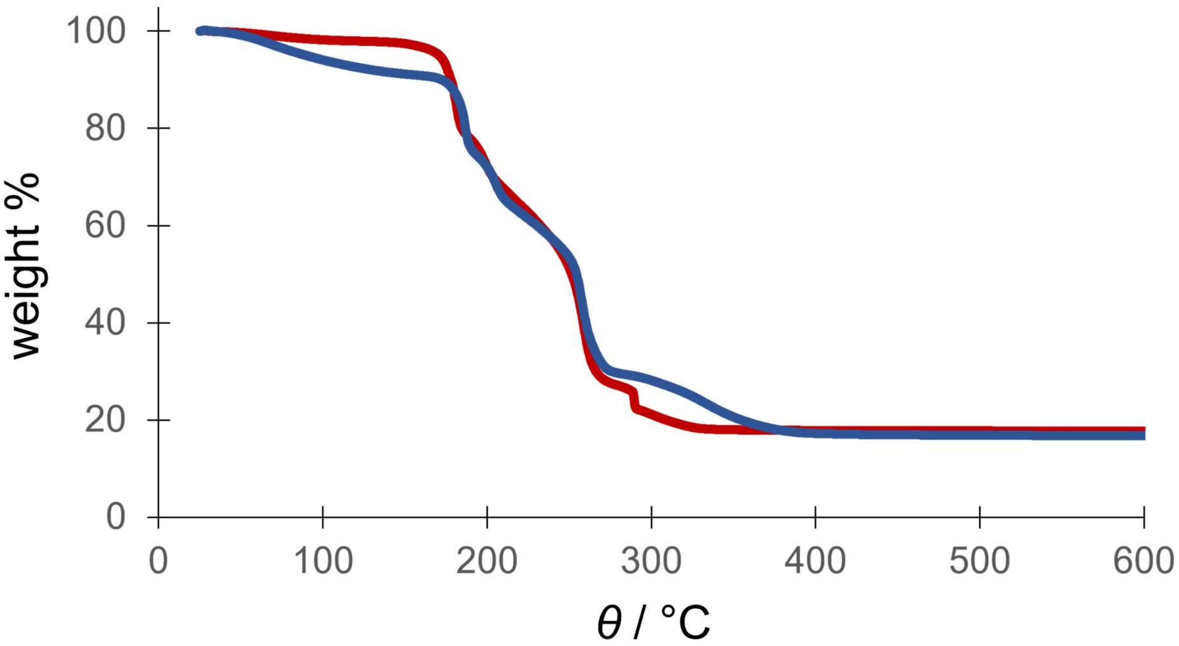

Figure S14. TGA curves of mechanochemically prepared {[Cu(μ-MeGly)(NO_3)(μ-3,3′-bpy)]·DMF·0.5H_2O}$_n$ (**1a**). The blue line represents a moist sample, measured immediately after synthesis, while the red line represents a sample of the same synthesis dried at ambient conditions.

Table S1. Water, phosphorus(V) oxide, and various salts whose saturated solutions were used to achieve specific relative humidities of the atmosphere at 20 °C.[1]

***RH* / %**	**Salt**
100	H_2O
95	KNO_3
85	KCl
79	NH_4Cl
75	NaCl
65	$CoCl_2$
54	$Mg(NO_3)_2$
43	K_2CO_3
33	$MgCl_2$
23	CH_3COOK
9	NaOH
0	P_4O_{10}

[1] L. Greenspan, J. Res. Natl. Bur. Stand. (U.S.), 81A (1977) 89–96.

Table S2. Crystallographic data for compounds **1a**, **1b**, **2a** and **2b**.

	**1a**	**1b**	**2a**	**2b**
Chemical formula	$C_{32}H_{44}N_{10}O_{13}Cu_2$	$C_{13}H_{14}N_4O_5Cu$ [+ solvent]	$C_{34}H_{48}N_{10}O_{13}Cu_2$	$C_{14}H_{16}N_4O_5Cu$ [+ solvent]
Molecular weight [g mol^{-1}]	903.85	369.82[+ solvent]	931.90	383.85[+ solvent]
Radiation [Å]	Cu*K*α (1.54184)	Cu*K*α (1.54184)	Cu*K*α (1.54184)	Cu*K*α (1.54184)
T [K]	170	100(2)	170	170
Crystal colour and shape	blue needle	blue needle	blue needle	dark blue needle
crystal size [mm^3]	0.03x0.04x0.14	0.21x0.10x0.08	0.07x0.07x0.34	028x0.34x0.36
Crystal system	orthorhombic	cubic	orthorhombic	cubic
Space group	*Ccc*2	*Ia*-3*d*	*Ccc*2	*Ia*-3*d*
a [Å]	20.3111(4)	43.2960(4)	21.0807(7)	43.6280(1)
b [Å]	23.5653(5)	43.2960(4)	23.3609(7)	43.6280(1)
c [Å]	8.3630(2)	43.2960(4)	8.3945(2)	43.6280(1)
α [°]	90	90	90	90
β [°]	90	90	90	90
γ [°]	90	90	90	90
V [Å^3]	4002.84(15)	81160(2)	4134.0(2)	83041.6(6)
Z	4	96	4	96
ρ [g cm^{-3}]	1.500	0.726[+solvent]	1.497	0.737[+solvent]
μ [mm^{-1}]	1.951	1.048	1.907	1.035
θ range [°]	3.8–67.5	2.9–75.0	2.8–67.5	2.5–70.0
Independent reflections (R_{int})	3281(0.054)	6794(0.059)	3713 (0.111)	6366 (0.041)
Observed refl. ($I > 2\ \sigma(I)$)	3137	3187	3634	5636
Number of parameters	250	208	224	217
R_1 (observed reflections)	0.0419	0.0664	0.0565	0.0475
wR_2 (all data)	0.1180	0.2716	0.1615	0.1644
S	1.09	1.07	1.08	1.07
max/min residual electron density [Å^{-3}]	0.43/-0.35	0.31/-0.32	0.58/-0.32	0.90/-0.48
CCDC no.	2583580	2583581	2583579	2583582

Table S3. Distances ([Å]) and bond angles ([°]) within the polyhedra of copper coordination spheres, in the crystal structures of **1a**, **1b**, **2a** and **2b**.

Bond lengths[Å]	**1a**	**1b**	**2a**	**2b**
Cu1-O11	1.947(6)	1.931(3)	1.945(19)	1.950(2)
Cu1-O11N	2.752(14)	2.708(5)	2.695(13)	2.676(3)
Cu1-N1	2.015(5)	2.000(4)	2.004(10)	2.030(2)
Cu1-N101	2.022(4)	2.007(4)	2.01(2)	2.016(2)
Cu1-$N_{3,3'\text{-bpy}}$	2.003(6) (N201)	2.004(4) (N111)	2.016(8) (N201)	2.029(2) (N111)
Cu1-O12[a]	2.403(4)	2.353(3)	2.416(10)	2.279(2)

Bond angles [°]	**1a**	**1b**	**2a**	**2b**
O11-Cu1-O11N	88.8(3)	83.94(15)	87.7(6)	84.29(8)
O11-Cu1-N1	84.2(2)	84.35(15)	84.0(7)	83.96(9)
O11-Cu1-N101	89.15(19)	89.47(14)	177.2(3)	176.68(9)
O11-Cu1-N201	177.06(17)	-	88.6(5)	-
O11-Cu1-O12[a]	87.46(17)	-	86.5(4)	-
O11N-Cu1-N1	92.0(3)	82.00(16)	93.4(5)	81.19(9)
O11N-Cu1-N101	84.1(3)	90.98(15)	92.1(4)	92.53(8)
O11N-Cu1-N201	91.1(3)	-	84.5(4)	-
O11N-Cu1-O12[a]	176.2(4)	-	173.6(6)	-
N1-Cu1-N101	172.4(3)	171.15(16)	98.8(5)	96.49(9)
N1-Cu1-N201	98.8(2)	-	172.4(10)	-
O12[a]-Cu1-N1	88.29(15)	-	88.5(3)	-
N101-Cu1-N201	87.9(2)	-	88.7(7)	-
O12[a]-Cu1-N101	95.12(16)	-	93.7(7)	-
O12[a]-Cu1-N201	92.6(2)	-	92.9(4)	-

[a]x,1-y,1/2+z

Table S4. Geometry of selected intra- and intermolecular hydrogen bonds ([Å],[°]) for compounds **1a, 1b, 2a** and **2b**.

	D−H…A	D−H [Å]	H…A [Å]	D…A [Å]	D−H…A [°]
**1a**	N1−H1…O13N[a]	1.00	2.23	3.185(15)	159
	N1−H1…O15N[a]	1.00	2.07	3.01(3)	155
**1b**	N1−H1...O11[b]	1.00	1.96	2.872(5)	150
**2a**	N1−H1...O12N[c]	1.00	2.18	3.148(13)	162
**2b**	N1−H1...O11[d]	1.00	2.03	2.936(3)	150

[a]1/2-x,3/2-y,z; [b]3/4-z,5/4-y,-3/4+x;[c]1/2-x,3/2-y,z ; [d]5/4-x,3/4-z,-1/4+y

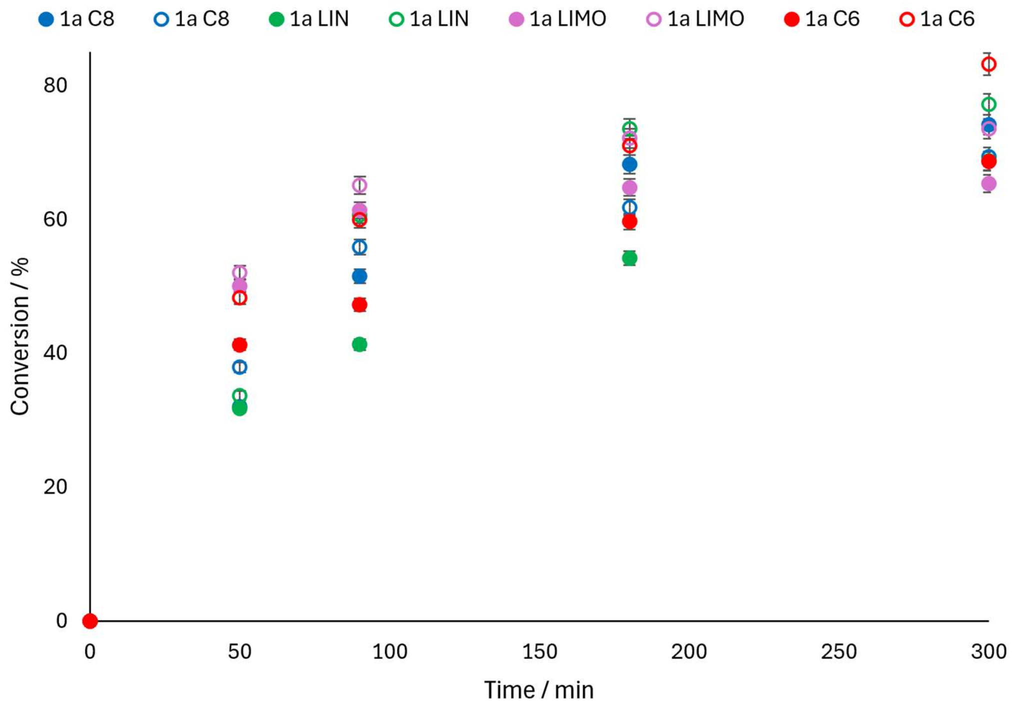

Figure S15. Kinetic profiles for cyclooctene (C8), linalool (LIN), limonene (LIMO) and cyclohexane (C6) conversion with error bars. A full dot represents the reaction without the addition of acetonitrile, while the empty dot represents the reaction with the addition of acetonitrile.

(a)

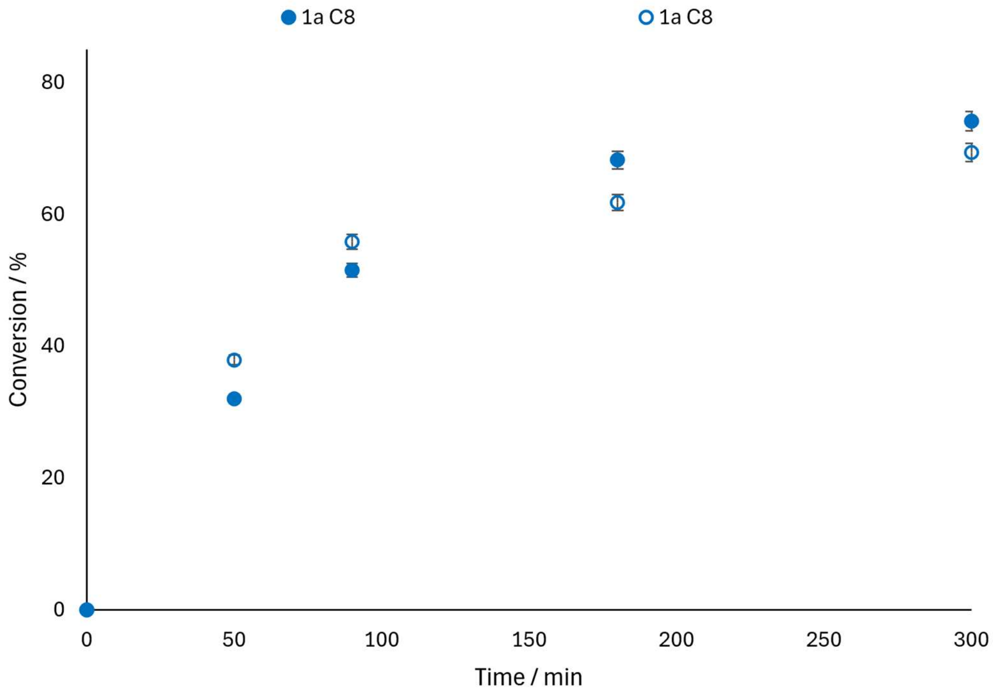
1a C8
1a C8
Conversion / %
80
60
40
20
0
0
50
100
150
200
250
300
Time / min

(b)

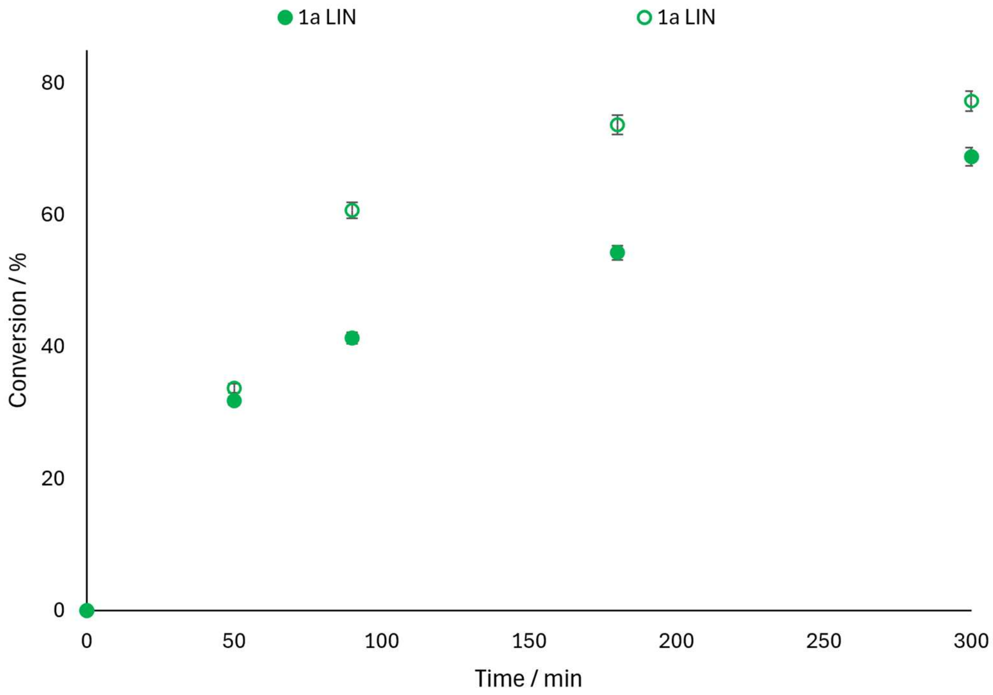
1a LIN
1a LIN
Conversion / %
80
60
40
20
0
0
50
100
150
200
250
300
Time / min

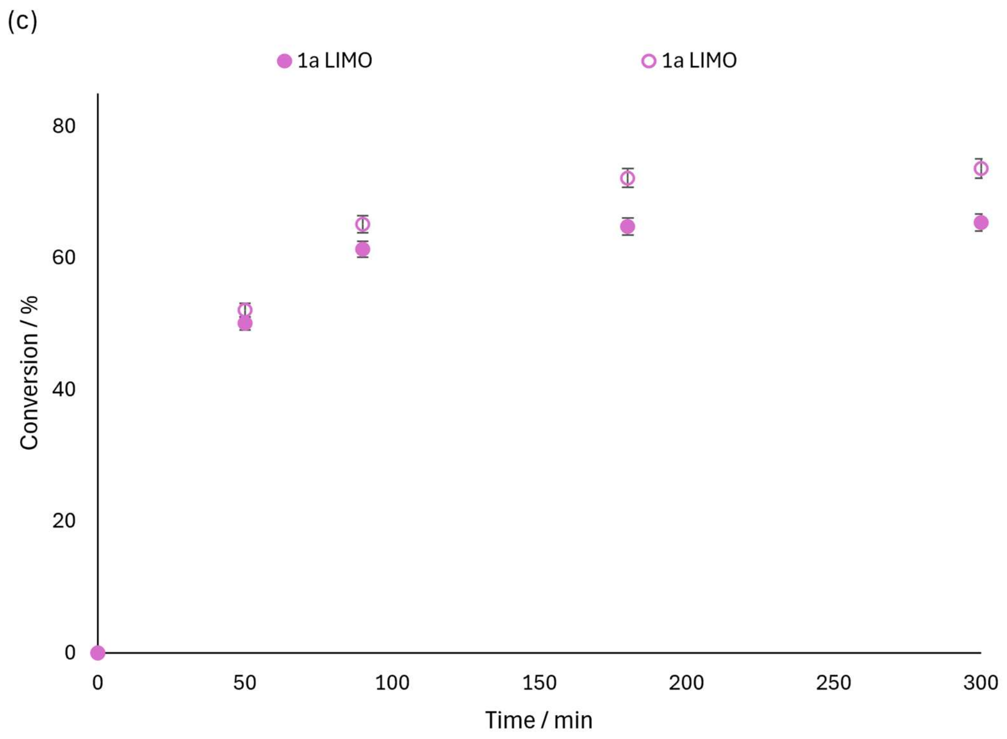

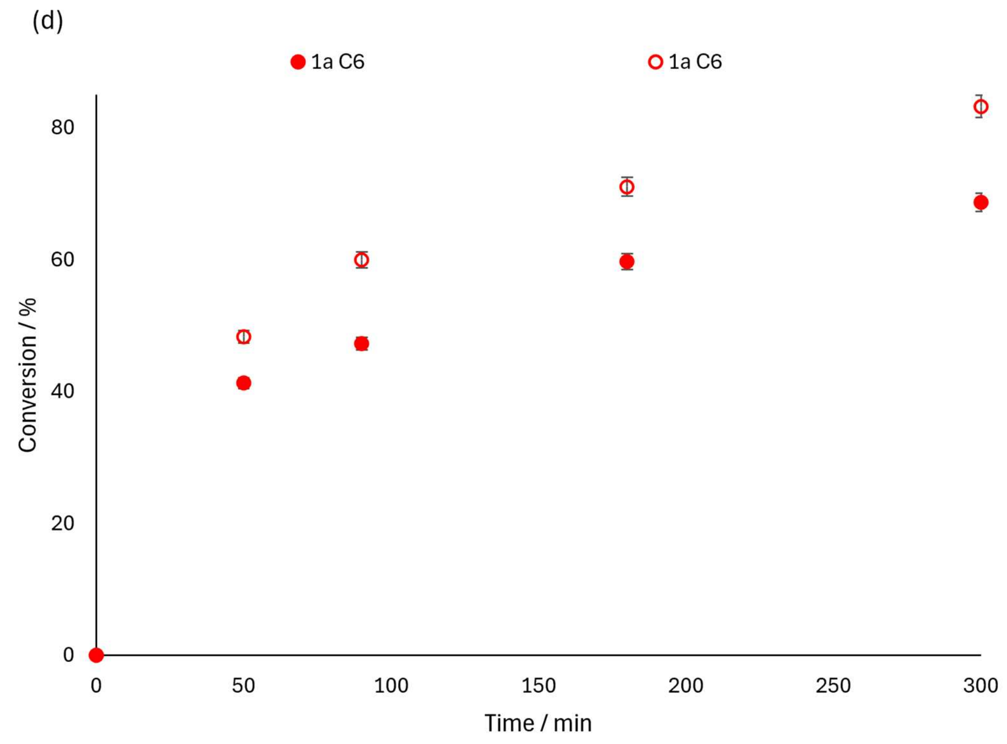

Figure S16. Kinetic profiles for: (a) cyclooctene (C8), (b) linalool (LIN), (c) limonene (LIMO), and (d) cyclohexane (C6) conversion with error bars. A full dot represents the reaction without the addition of acetonitrile, while the empty dot represents the reaction with the addition of acetonitrile.

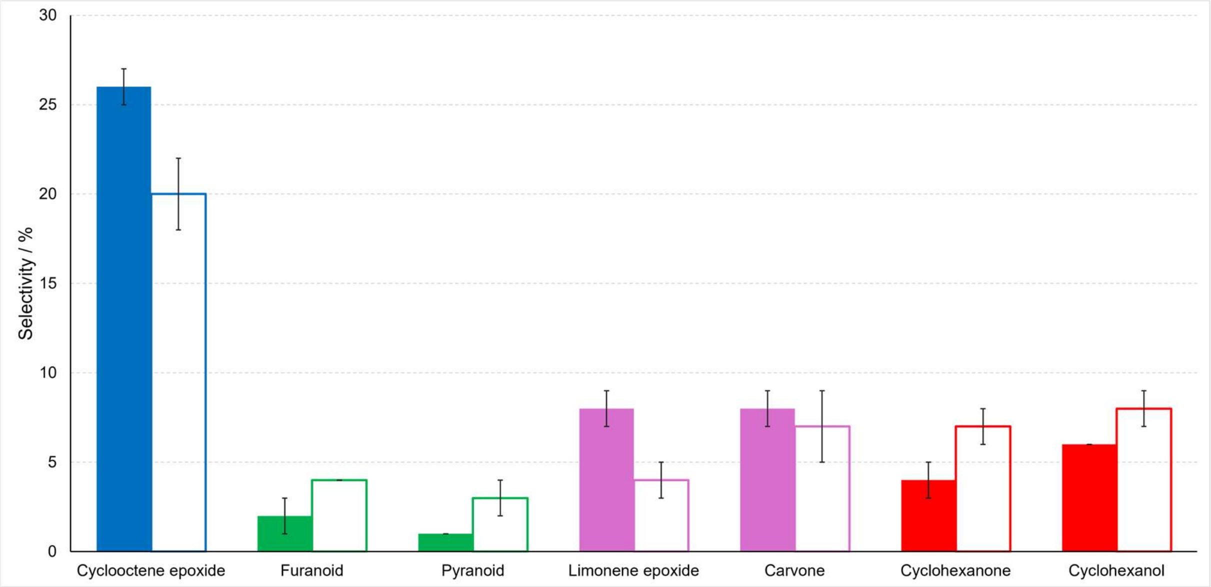

Figure S17. Product selectivity in the oxidation of cyclooctene (blue), linalool (green), limonene (purple), and cyclohexane (red) using TBHP, with and without acetonitrile. Full bars represent reactions without addition of acetonitrile and empty bars represent reactions with addition of acetonitrile.

Table S5. Catalytic performance and product yields for the oxidation of different substrates using TBHP in the presence and absence of MeCN.

**Substrate**	**Condition**	**Conversion (%)**	**Product**	**Selectivity (%)**	**Yield (%)**	**Mass balance* %**
**Cyclooctene**	TBHP	74	cyclooctene oxide	26	19.24	45
	TBHP+MeCN	69	cyclooctene oxide	20	13.80	45
**Linalool**	TBHP	69	Furanoid	2	1.38	33
			Pyranoid	1	0.69	
	TBHP+MeCN	77	Furanoid	4	3.08	28
			Pyranoid	3	2.31	
**Limonene**	TBHP	65	Epoxide	8	5.20	45
			Carvone	8	5.20	
	TBHP+MeCN	74	Epoxide	4	2.96	34
			Carvone	7	5.18	
**Cyclohexane**	TBHP	69	Cyclohexanone	4	2.76	39
			Cyclohexanol	6	4.14	
	TBHP+MeCN	83	Cyclohexanone	7	5.81	29
			Cyclohexanol	8	6.64	

*the mass balance was calculated according to the identified species in the organic phase.

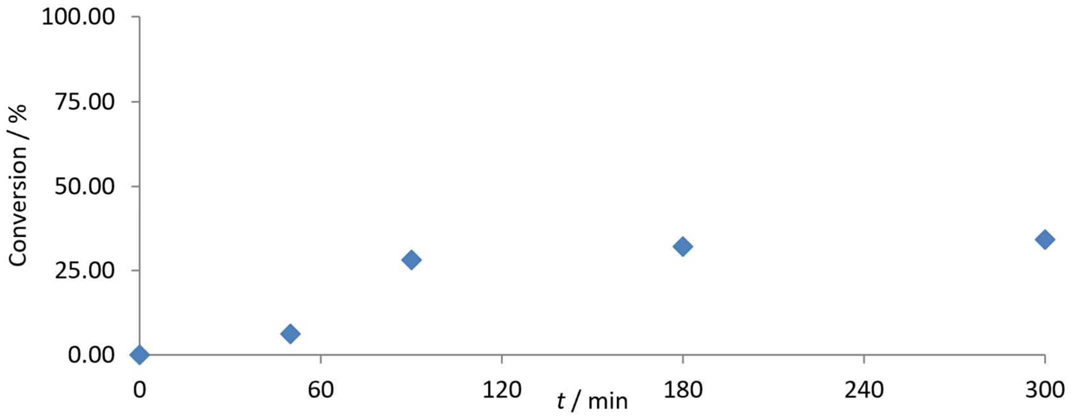

Figure S18. Kinetic profile for oxidation reaction of cyclohexane without the catalyst.

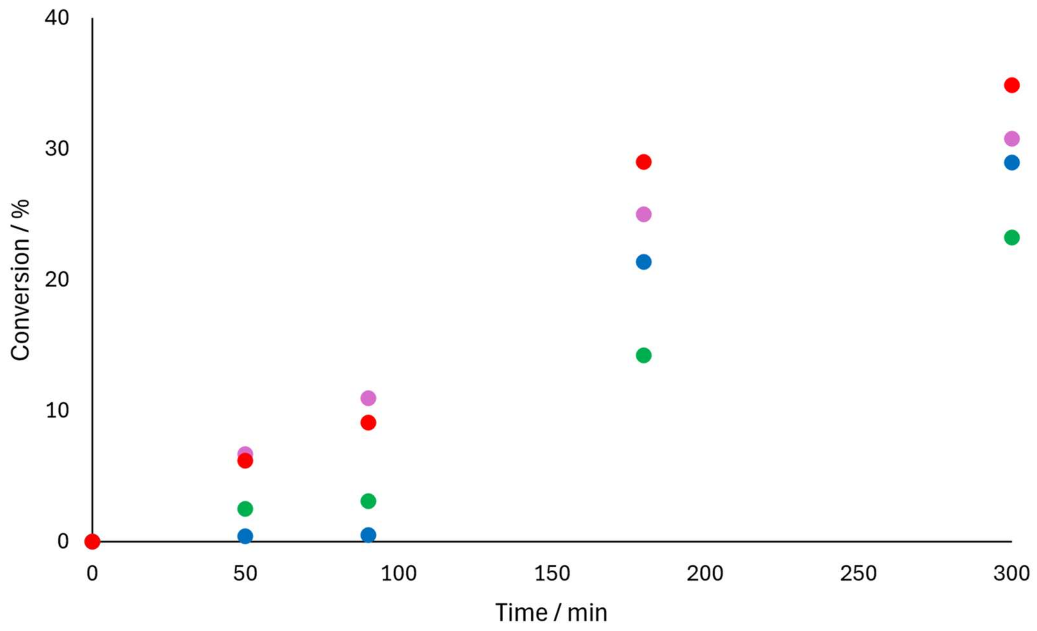

Figure S19. Kinetic profile for oxidation reaction of cyclohexane with $Cu(NO_3)_2$ (blue), methyl glycinate (green), 3,3′-bipyridine (purple), or a mixture of $Cu(NO_3)_2$, methyl glycinate, and 3,3′-bipyridine in the corresponding Cu:MeGly:bipy ratio (red).

Table S6. Conversion and product selectivity for the oxidation of cyclohexane with $Cu(NO_3)_2$, methyl glycinate, 3,3′-bipyridine, or a mixture of $Cu(NO_3)_2$, methyl glycinate, and 3,3′-bipyridine in the corresponding Cu:MeGly:bipy ratio. *n*((pre)catalyst)/*n*(substrate)/*n*(oxidant) = 0.1 mmol / 20 mmol / 40 mmol.

**Catalyst**	**Cyclohexane Conversion (%)**	**Selectivity towards Cyclohexanol and Cyclohexanone (%)**
$Cu(NO_3)_2$	29	0
methyl glycinate	23	0
3,3′-bipyridine	31	0
Cu:MeGly:bipy	35	0

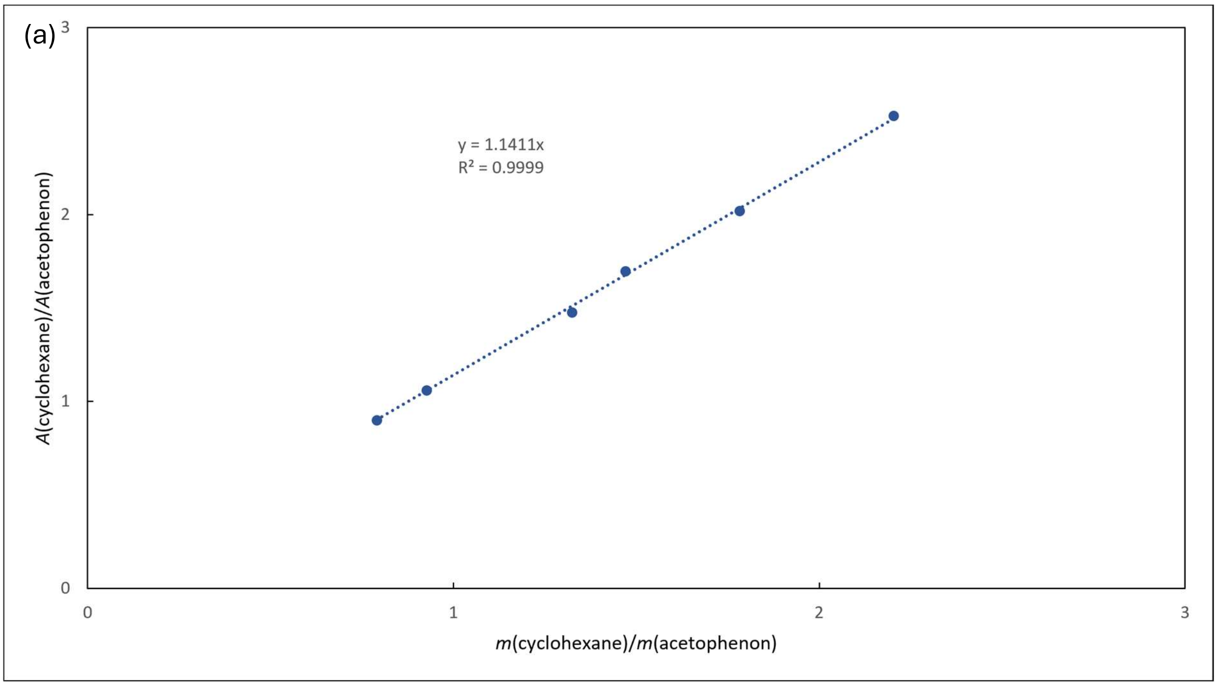

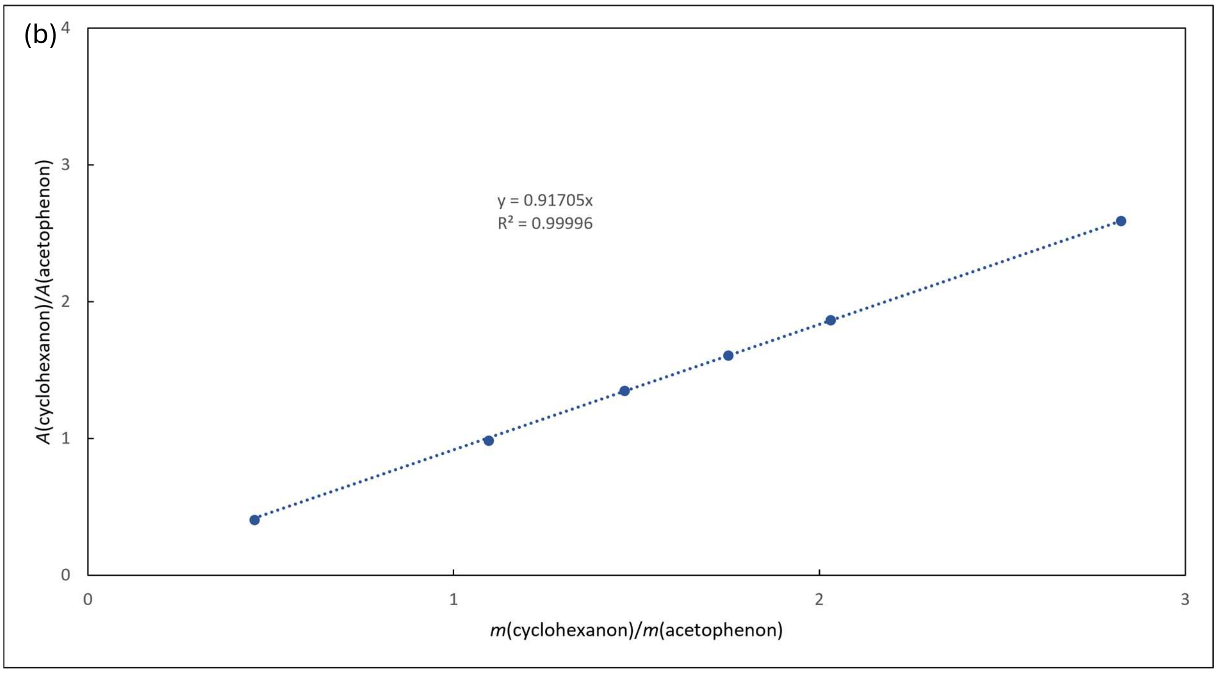
(b)
y = 0.91705x
R² = 0.99996
A(cyclohexanon)/A(acetophenon)
m(cyclohexanon)/m(acetophenon)
0
1
2
3
4

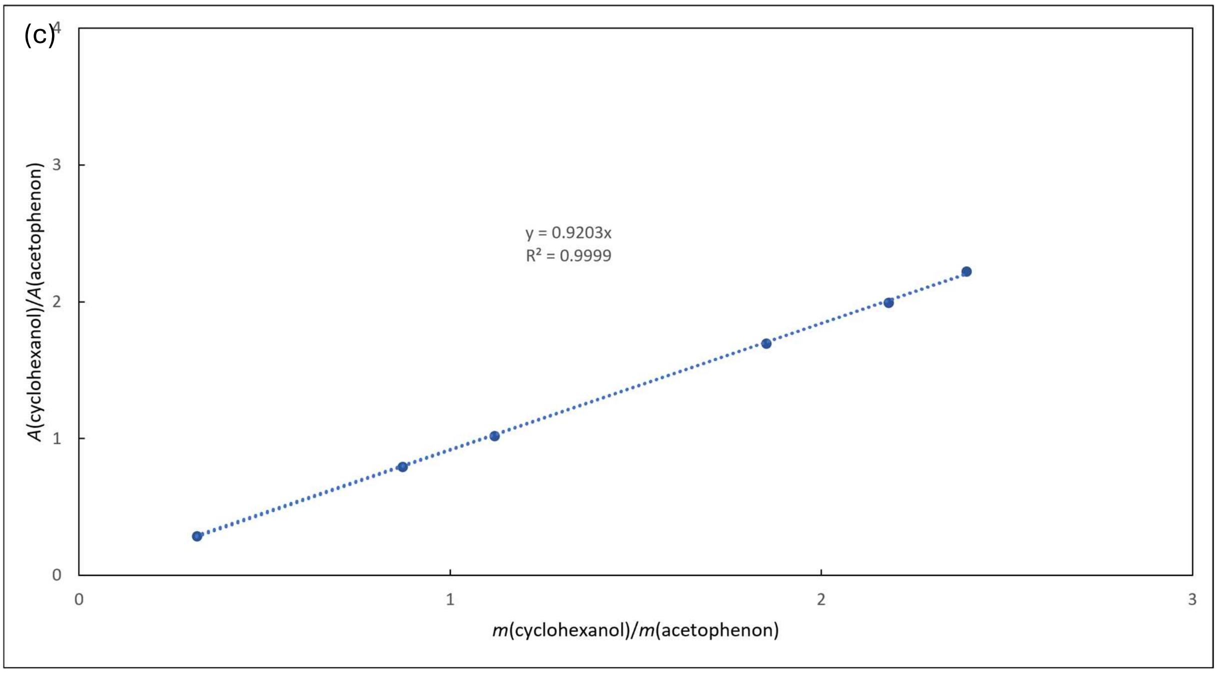
(c)
y = 0.9203x
R² = 0.9999
A(cyclohexanol)/A(acetophenon)
m(cyclohexanol)/m(acetophenon)
0
1
2
3
4

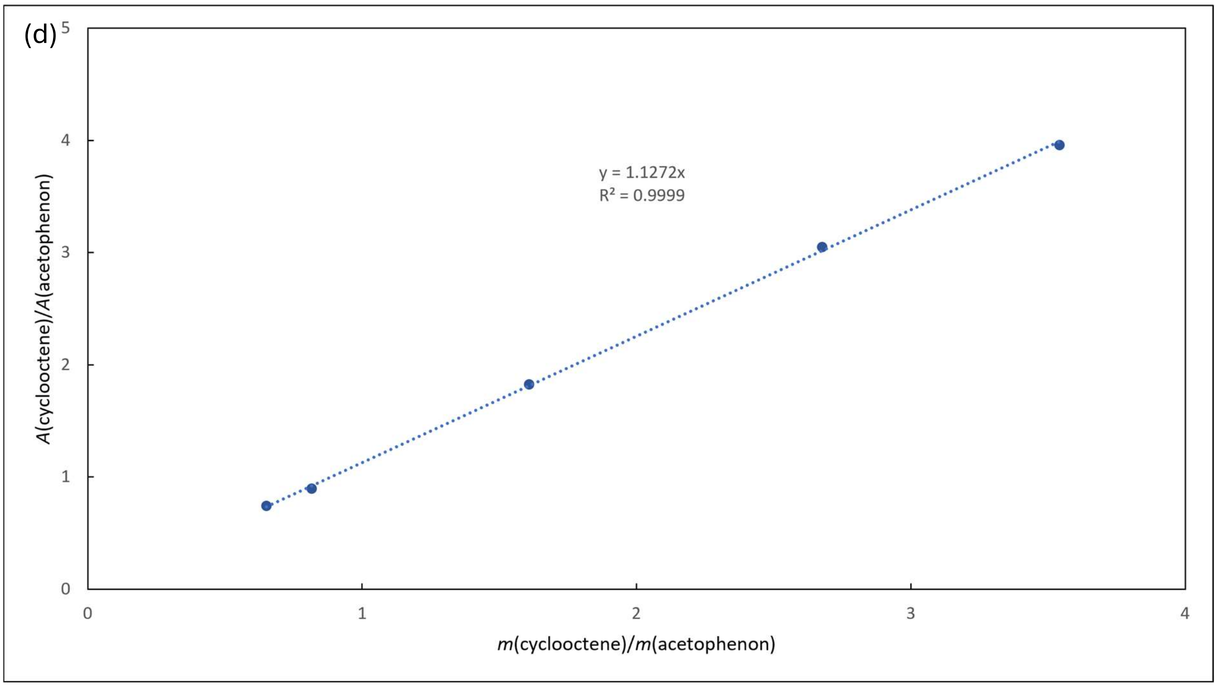
(d)
y = 1.1272x
R² = 0.9999
A(cyclooctene)/A(acetophenon)
m(cyclooctene)/m(acetophenon)
0
1
2
3
4
5

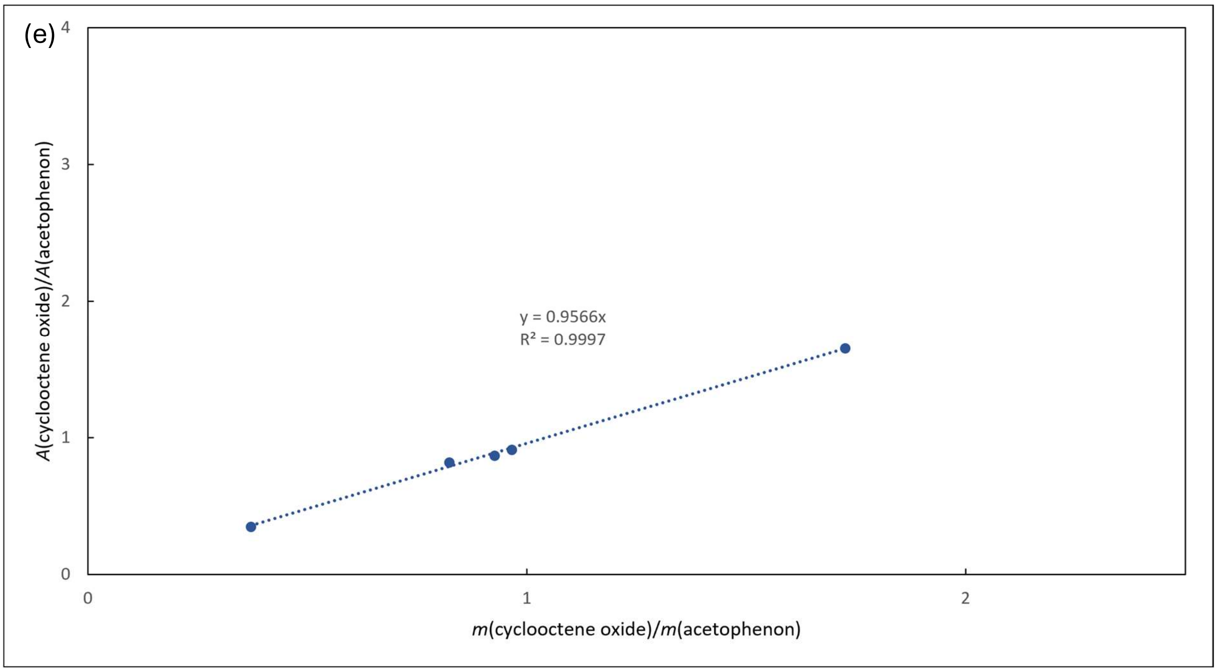
(e)
y = 0.9566x
R² = 0.9997
A(cyclooctene oxide)/A(acetophenon)
m(cyclooctene oxide)/m(acetophenon)
0
1
2
3
4

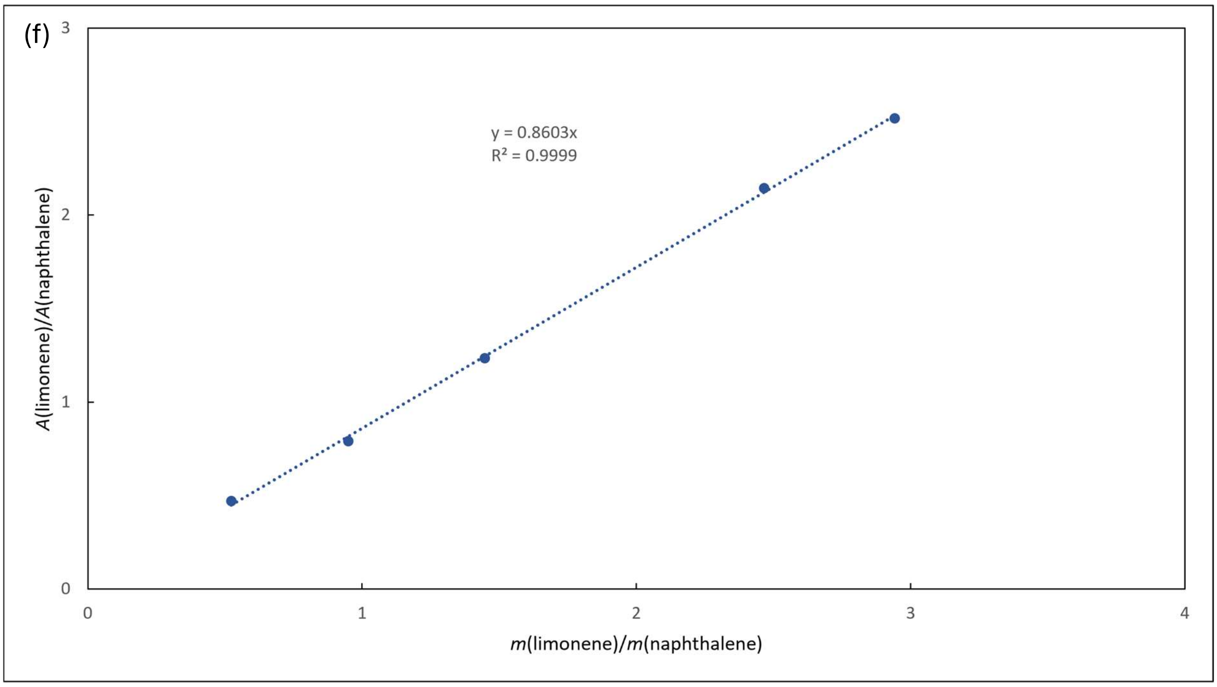
(f)
y = 0.8603x
R² = 0.9999
A(limonene)/A(naphthalene)
m(limonene)/m(naphthalene)
0
1
2
3
4

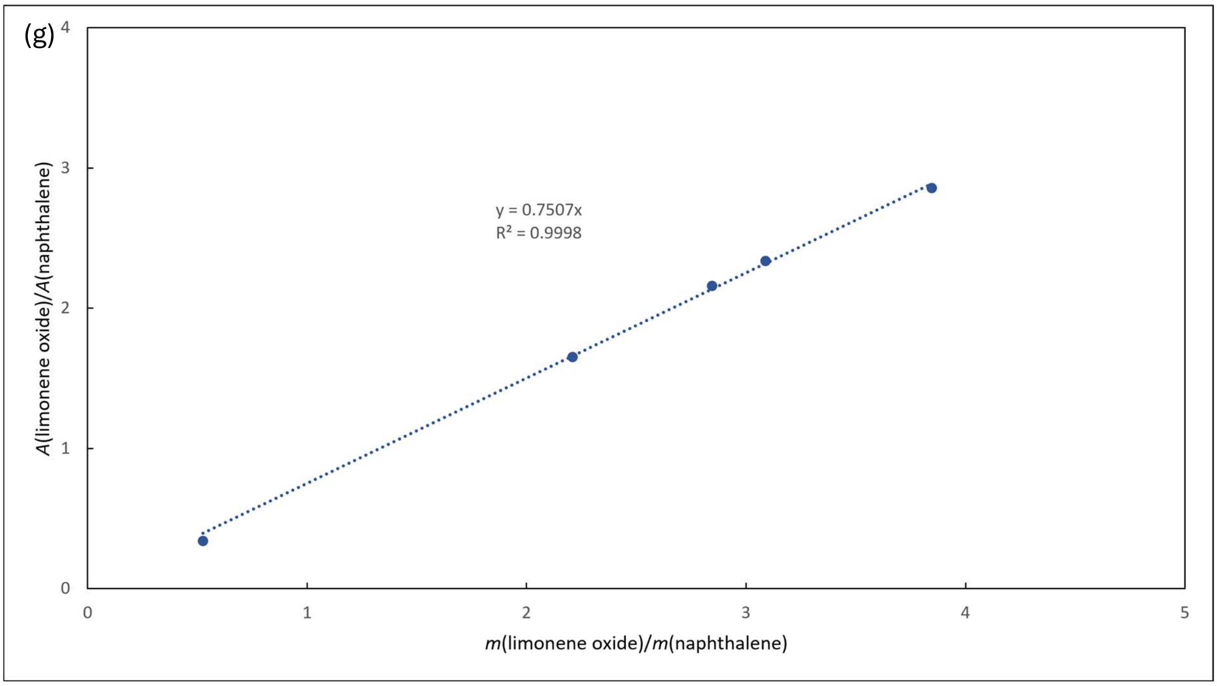
(g)
y = 0.7507x
R² = 0.9998
A(limonene oxide)/A(naphthalene)
m(limonene oxide)/m(naphthalene)
0
1
2
3
4
5

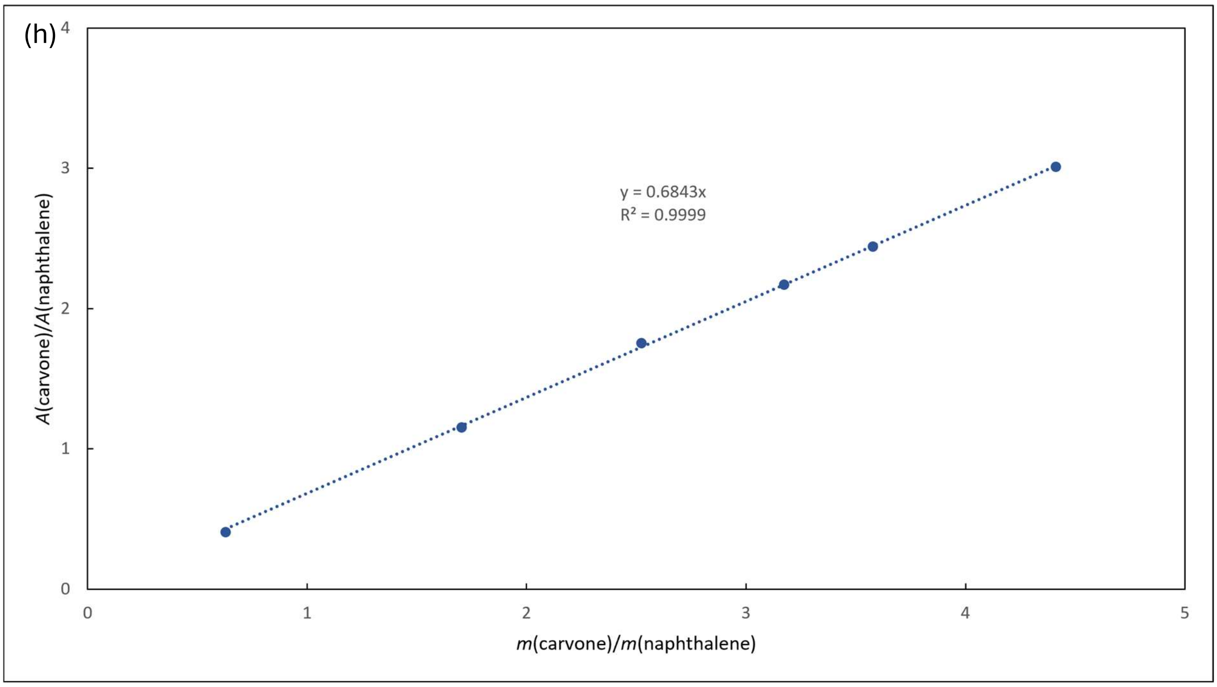
(h)
y = 0.6843x
R² = 0.9999
A(carvone)/A(naphthalene)
m(carvone)/m(naphthalene)
0
1
2
3
4
5

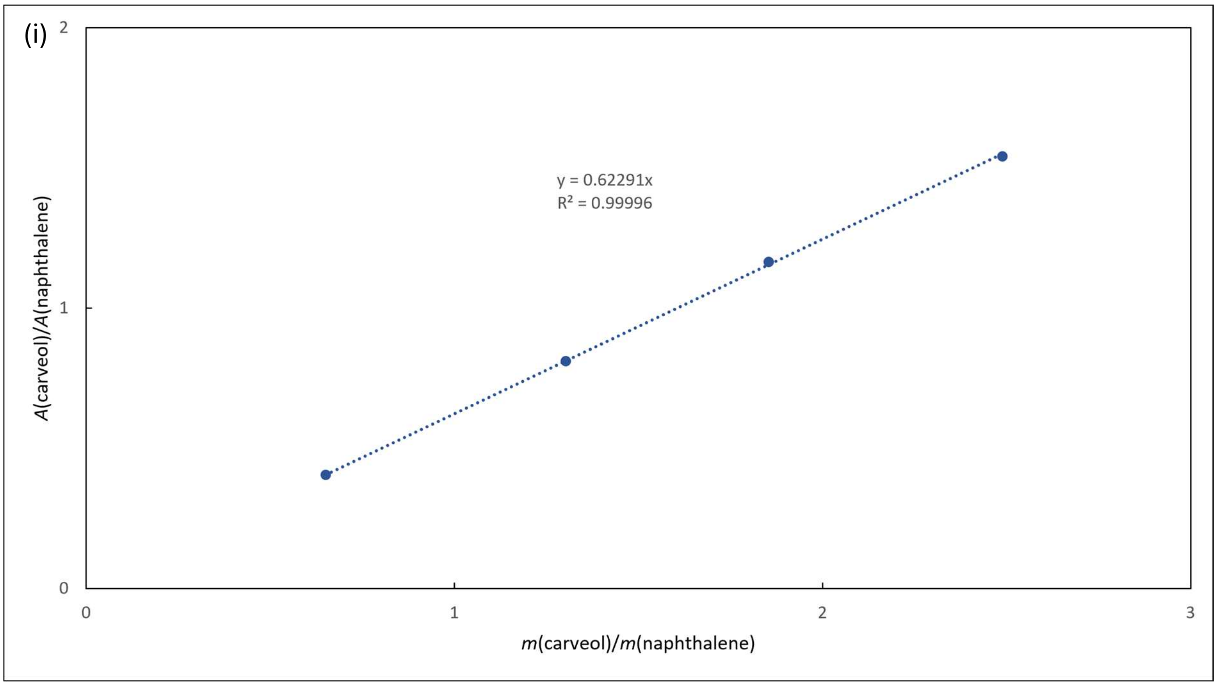
(i)
y = 0.62291x
R² = 0.99996
A(carveol)/A(naphthalene)
m(carveol)/m(naphthalene)
0
1
2
3

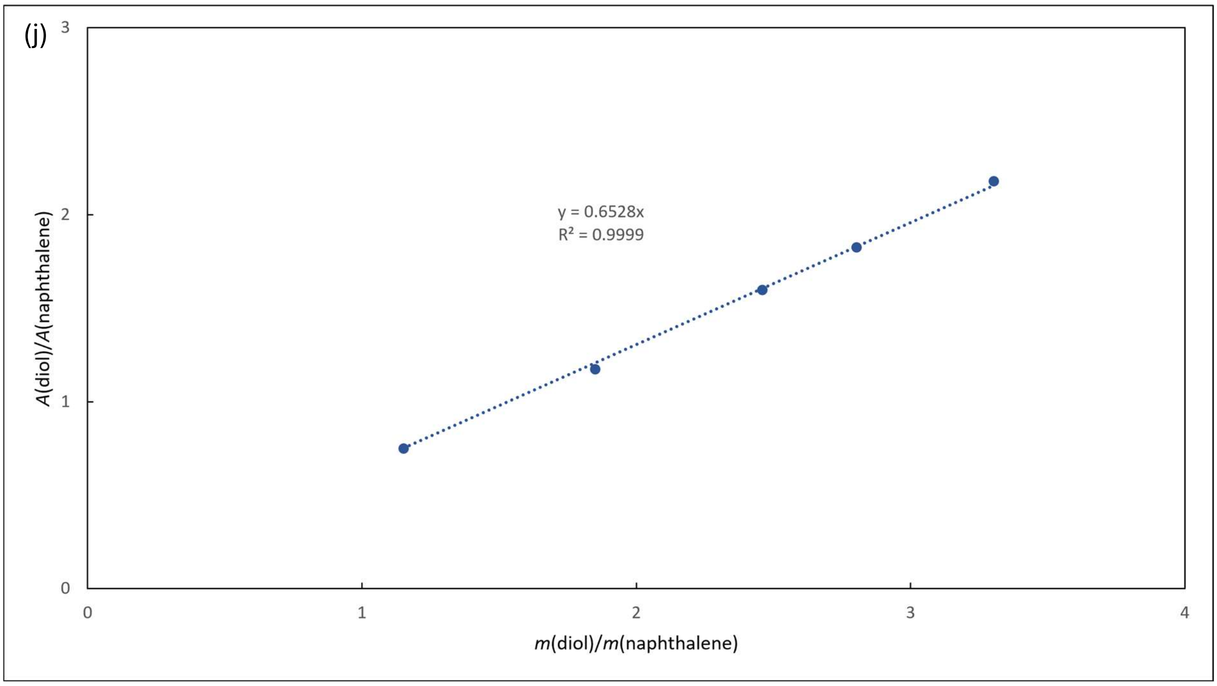
(j)
y = 0.6528x
R² = 0.9999
A(diol)/A(naphthalene)
m(diol)/m(naphthalene)
0
1
2
3
0
1
2
3
4

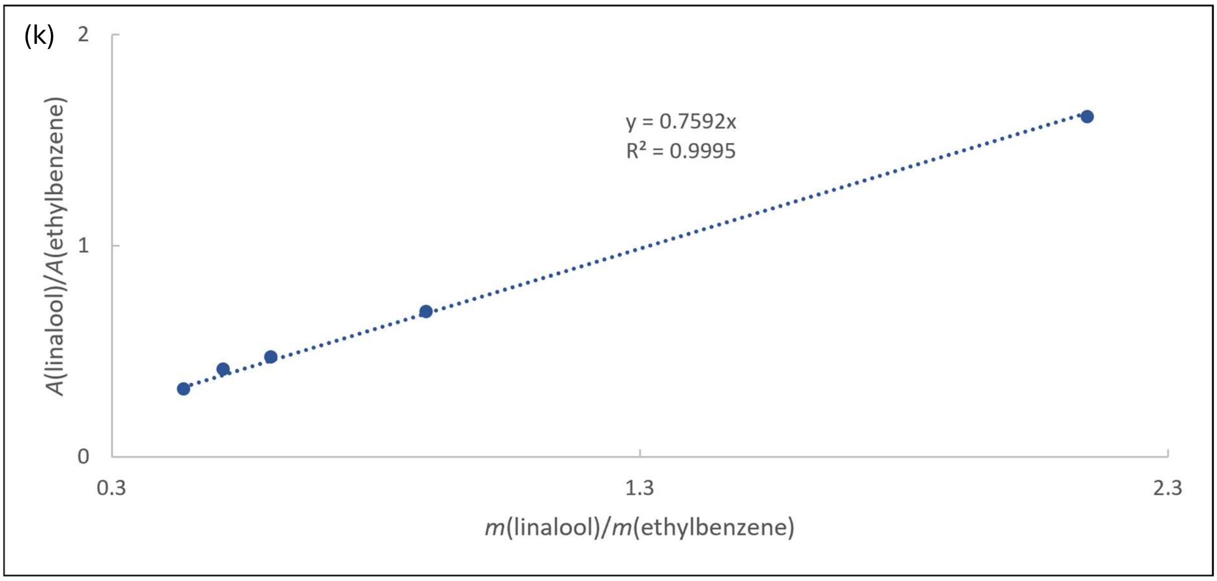
(k)
y = 0.7592x
R² = 0.9995
A(linalool)/A(ethylbenzene)
m(linalool)/m(ethylbenzene)
0
1
2
0.3
1.3
2.3

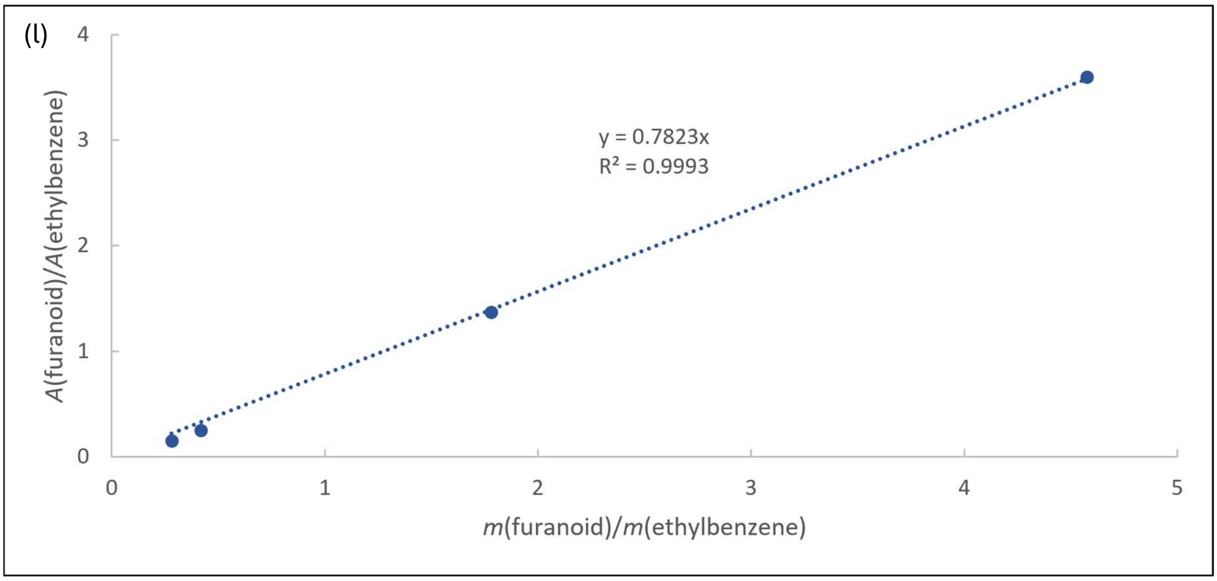

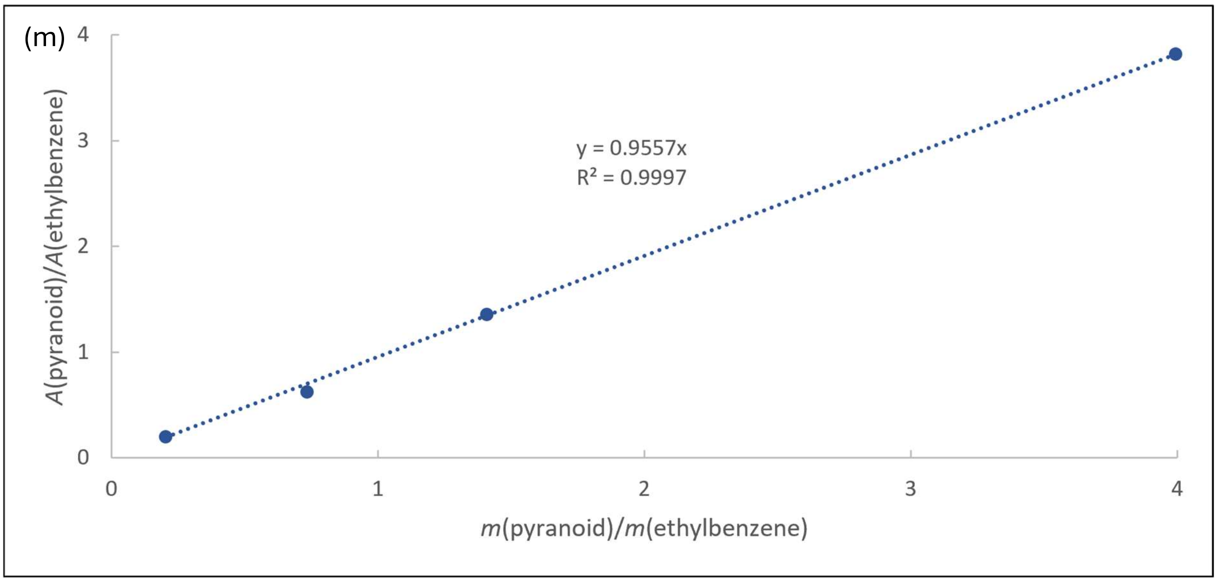

Figure S20. Calibration curves for (a) cyclohexane, (b) cyclohexanone, (c) cyclohexanol, (d) cyclooctene, (e) cyclooctene oxide, (f) limonene, (g) limonene oxide, (h) carvone, (i) carveol, (j) diol, (k) linalool, (l) furanoid, (m) pyranoid.

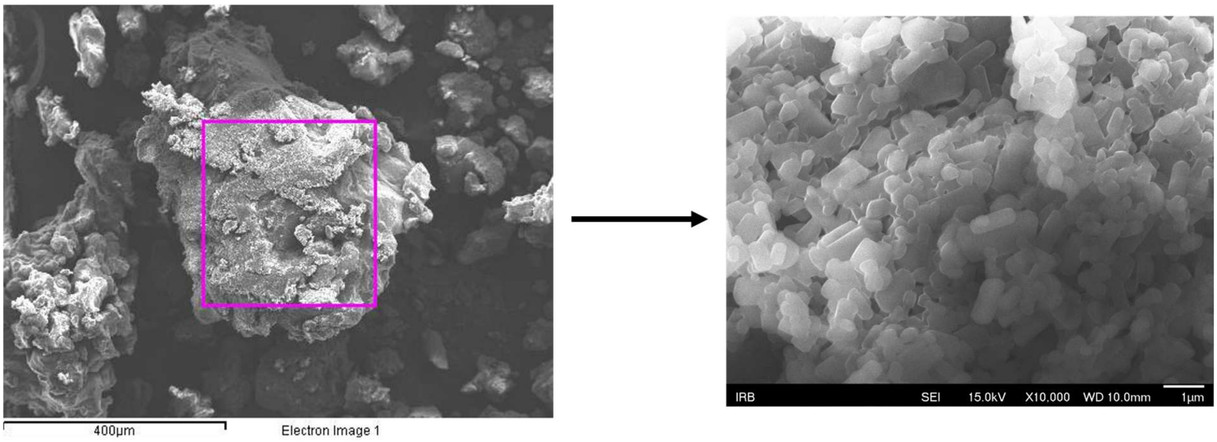

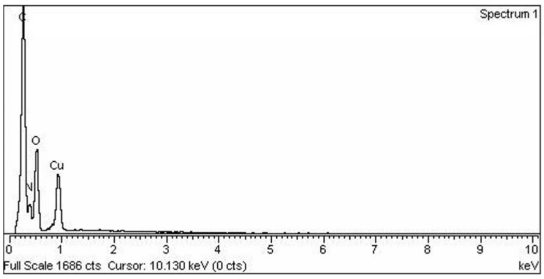

Element	Weight%	Atomic%
C (K)	41.25	53.18
N (K)	16.97	18.76
O (K)	24.70	23.90
Cu (L)	17.07	4.16
Totals	100.00	

Figure S21. SEM images and the EDS analysis of the mechanochemically prepared sample of **1a**.
